# Single‐Field Evolution Rule Governs the Dynamics of Representational Drift in Mouse Hippocampal Dorsal CA1 Region

**DOI:** 10.1002/advs.202509532

**Published:** 2025-11-28

**Authors:** Cong Chen, Shuyang Yao, Sihui Cheng, Jiayi Tian, Ang Li, Yusen Yan, Xiang Zhang, Yuanjing Liu, Yumeng Wang, Qichen Cao, Chenglin Miao

**Affiliations:** ^1^ State key laboratory of Membrane biology, School of Life Science Peking university Beijing 100871 China; ^2^ PKU‐IDG/McGovern Institute for Brain Research Peking university Beijing 100871 China; ^3^ Peking‐Tsinghua Center for Life Sciences Beijing 100871 China; ^4^ Department of Biology University of North Carolina at Chapel Hill Chapel Hill NC 27599 USA; ^5^ Chinese Institute for Brain Research (CIBR) Beijing 102206 China

**Keywords:** activity‐dependent dynamics, hippocampal dorsal CA1, place cell, representational drift, single‐field evolution

## Abstract

Hippocampal place codes change substantially across days, yet the mechanisms governing their temporal evolution remain incompletely understood. To quantitatively characterize this process, longitudinal one‐photon calcium imaging of dorsal CA1 neurons in mice is performed for up to 56 days across multiple goal‐oriented navigation tasks. Parallel to mice's improvements in maze learning and navigational performance, thousands of place fields exhibit complex evolutionary trajectories characterized by formation, disappearance, and retention. Leveraging statistical analyses and sequential learning models (e.g., recurrent neural networks and hidden Markov models), a position‐, decision‐making‐, and novelty‐irrelevant Single‐Field Evolution Rule (SFER) is identified: active states of a place field increase its probability of remaining active in the subsequent session, whereas inactive states reduce it. Simulations of a stochastic discrete dynamical system defined by SFER reveal that the novelty‐related stabilization of dCA1 place codes at the population level emerges as a collective outcome of the novelty‐irrelevant SFER. Among the ten tested models, SFER‐based models provide the best predictions of field evolutions, and an extended version incorporating inter‐field interactions and day‐to‐day fluctuations effectively captured coordinated multi‐field evolution. This framework offers a novel, efficient, and parsimonious approach that demonstrates the derivational relationship between activity‐dependent single‐field evolution and population‐level drift dynamics in the hippocampus.

## Graphical abstract

Long‐term hippocampal place‐code dynamics are investigated using calcium imaging across weeks of maze navigation. Analyses reveal a novelty‐irrelevant Single‐Field Evolution Rule (SFER), where active fields promote persistence and inactive fields decline. Simulations demonstrate that novelty‐related stabilization of population codes emerges from this rule. This framework provides a parsimonious explanation linking single‐field activity to collective drift in hippocampal representations.

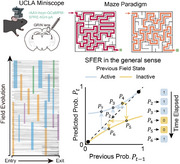

## 1 Introduction

Dynamism and stability, seemingly incompatible concepts, coexist within the neural network to balance reliable memory storage with the integration of new information. In the hippocampal dCA1 region, the exploration of the relationship between dynamic and stable aspects of neuronal representation has been ongoing since the discovery of place cells,^[^ ^1^ ^]^ which are pyramidal neurons that encode specific spatial locations known as place fields. Intuitively, the spatial representation of an unchanging environment by the dCA1 should remain stable, providing consistent space coding essential for establishing a cognitive map.^[^ ^2^ ^]^ With cases of super stable place fields being documented,^[^ ^3^, ^4^ ^]^ however, extensive research has revealed a pronounced dynamic nature of hippocampal spatial representation even in stable environmental conditions, as observed during both novel states and familiar states.^[^ ^4^, ^5^, ^6^, ^7^, ^8^, ^9^, ^10^ ^]^ This phenomenon, termed representational drift, involves substantial changes such as the formation, disappearance, and translocation of place fields and is accompanied by profound refinement of neuronal representation^[^ ^11^ ^]^ while still preserving the statistical structure of field properties.^[^ ^8^ ^]^

Hippocampal representational drift manifests across two distinct timescales: the behavioral timescale and the cross‐day timescale. At the behavioral timescale, place fields can emerge immediately during an animal's first traversal of a novel environment,^[^ ^12^ ^]^ likely reflecting pre‐strengthened synaptic connections.^[^ ^13^ ^]^ They can also arise on a trial‐to‐trial basis, driven in part by postsynaptic depolarization events—including dendritic plateau potentials,^[^ ^14^, ^15^, ^16^, ^17^, ^18^ ^]^ backpropagating action potentials, NMDA‐dependent spikes,^[^ ^19^, ^20^ ^]^ and transient dendritic disinhibition^[^ ^20^ ^]^—that rapidly reconfigure synaptic weights through behavioral timescale synaptic plasticity (BTSP).^[^ ^15^, ^17^, ^18^ ^]^ After formation, early studies reported that hippocampal place fields undergo experience‐ and NMDA receptor–dependent^[^ ^21^ ^]^ backward shifting within a session.^[^ ^22^, ^23^, ^24^, ^25^ ^]^ This phenomenon is theoretically attributed to the temporal asymmetry of plasticity rules,^[^ ^21^ ^]^ previously linked to spike timing–dependent plasticity (STDP) across CA3–CA1 connections.^[^ ^23^, ^26^, ^27^, ^28^ ^]^ More recently, however, it has been modeled by BTSP, which better accounts for a broader range of trial‐by‐trial place‐field dynamics beyond simple backward shifting, including forward shifting and novelty‐dependent features.^[^ ^17^ ^]^ Backward shifting reflects gradual, lap‐by‐lap representational dynamics, with novelty‐related differences between CA1 and its upstream CA3 region.^[^ ^24^, ^25^ ^]^ In addition to these smooth shifts, more pronounced translocations of place fields have been observed and are thought to be experience‐driven^[^ ^9^, ^29^ ^]^ and novelty‐ or learning‐related.^[^ ^30^ ^]^ For example, dCA1 spatial representations can rapidly reorganize via BTSP, recruiting additional place fields to encode reward‐associated positions.^[^ ^30^ ^]^ As environments become familiar, the intensity of plasticity‐inducing events—including the branch spike prevalence,^[^ ^20^ ^]^ place field backward shifting^[^ ^24^ ^]^, and BTSP‐like signatures^[^ ^31^ ^]^—diminishes, while neuronal stability^[^ ^11^, ^16^, ^31^, ^32^, ^33^ ^]^ and firing selectivity^[^ ^11^, ^32^ ^]^ increase. Despite this reduction in plasticity‐inducing events, substantial representational drift remains evident across days, with population codes progressively decorrelating from their initial configuration over weeks.^[^ ^6^, ^7^, ^8^, ^9^ ^]^ Consistent with anatomical evidence of week‐scale synaptic plasticity^[^ ^34^ ^]^ and neurogenesis,^[^ ^35^, ^36^, ^37^ ^]^ additional mechanisms must therefore underlie cross‐day representational drift even under conditions of behavioral familiarization.

Apart from the hippocampus, cross‐day representational drift has been observed across several brain regions,^[^ ^38^, ^39^, ^40^, ^41^, ^42^ ^]^ although recent studies reported limited drift in the motor cortex.^[^ ^43^, ^44^ ^]^ This observation has led to intuitive theories linking representational drift to synaptic plasticity.^[^ ^45^ ^]^ Some have conjectured that cross‐day representational drift may reflect noisy synaptic plasticity during learning,^[^ ^45^, ^46^ ^]^ such that high‐dimensional neuronal activity underlying certain behaviors remains invariant on a low‐dimensional manifold,^[^ ^10^, ^43^, ^47^, ^48^ ^]^ a framework plausibly supported by the representational drift with a constant rate observed in the piriform cortex.^[^ ^40^ ^]^ In the dCA1, however, cumulative studies have reported increasing cross‐day stability of spatial representation^[^ ^7^, ^9^, ^12^, ^16^, ^49^, ^50^ ^]^ and higher drift rate in novel environments,^[^ ^12^ ^]^ suggesting that drift rate is a function of experience. Beyond novelty‐related stabilization at the population level, single‐field persistence is influenced by neuron‐specific, activity‐dependent properties, including fos‐expression,^[^ ^51^ ^]^ branch spike prevalence,^[^ ^19^ ^]^ and intrinsic excitability.^[^ ^52^ ^]^ Inhibition of protein synthesis^[^ ^53^ ^]^ or blockade of NMDA receptors^[^ ^54^ ^]^ is sufficient to impair cross‐day place‐field stability. Moreover, optogenetic manipulations have demonstrated that the activity of a single field exerts feedback on its subsequent strength within the same session.^[^ ^55^ ^]^ Together, this evidence underscores the importance of activity‐dependent plasticity in shaping cross‐day representational drift. However, critical questions remain: 1) does the activity history of a single place field profoundly shape its future fate? 2) if so, what rules govern this shaping? and 3) can activity‐dependent plasticity at the single‐field level, by itself, collectively give rise to the empirical novelty‐related drift dynamics of hippocampal place codes?

A fundamental requirement for quantitatively exploring representational dynamics is the availability of a sufficient quantity of place fields. While an acceptable number of place fields can be identified in small‐scale or simple environments, having an excess of place fields is desirable. One technically feasible approach to expand field number is a focus on multi‐field place cells, which have been widely documented across various experimental paradigms, including large‐scale linear tracks,^[^ ^8^, ^56^, ^57^ ^]^ open field,^[^ ^58^, ^59^, ^60^ ^]^ and complex mazes,^[^ ^61^ ^]^ and are proposed to represent a generalized pattern for the dCA1 pyramidal neurons to encode space on a natural scale. Particularly, a more substantial percentage of active units (e.g., 83.4%) can be classified as place cells in large‐scale environments,^[^ ^56^, ^57^ ^]^ providing additional increments in the number of place fields.

While place cells in mice inherently tend to express multiple fields^[^ ^62^, ^63^ ^]^ in standard enclosures compared to rats, we designed two complex mazes, Maze A (MA) and Maze B (MB), and trained mice to navigate within them. Using month‐long one‐photon calcium imaging during training, we continuously tracked multi‐field place cells across sessions. Sequential learning models revealed a strong history dependence in the evolution of single place fields, which we formalized as the Single‐Field Evolution Rule (SFER): active states of a place field increase its probability of reappearing in the subsequent session, whereas inactive states reduce it. Importantly, this rule operates independently of environmental novelty. SFER conceptualizes place‐field evolution as a stochastic discrete dynamical system, and simulations demonstrate that novelty‐related stabilization of dCA1 population codes naturally emerges as a collective outcome of this novelty‐irrelevant rule. To further validate the predictive power of SFER, we compared it against models incorporating behavioral, temporal, and field‐specific factors, finding that many SFER‐based models consistently outperformed alternatives. By introducing field‐assignment mechanisms and day‐to‐day fluctuations, we further showed that SFER can incorporate inter‐field interactions into field‐evolution modeling. This framework delineates single‐field evolution while balancing simplicity, interpretability, extensibility, and efficiency, offering unique insights into the mechanisms underlying dCA1 representational drift.

## 2 Results

### 2.1 The Engagement of Maze‐Navigation Paradigm

**Figure 1:**
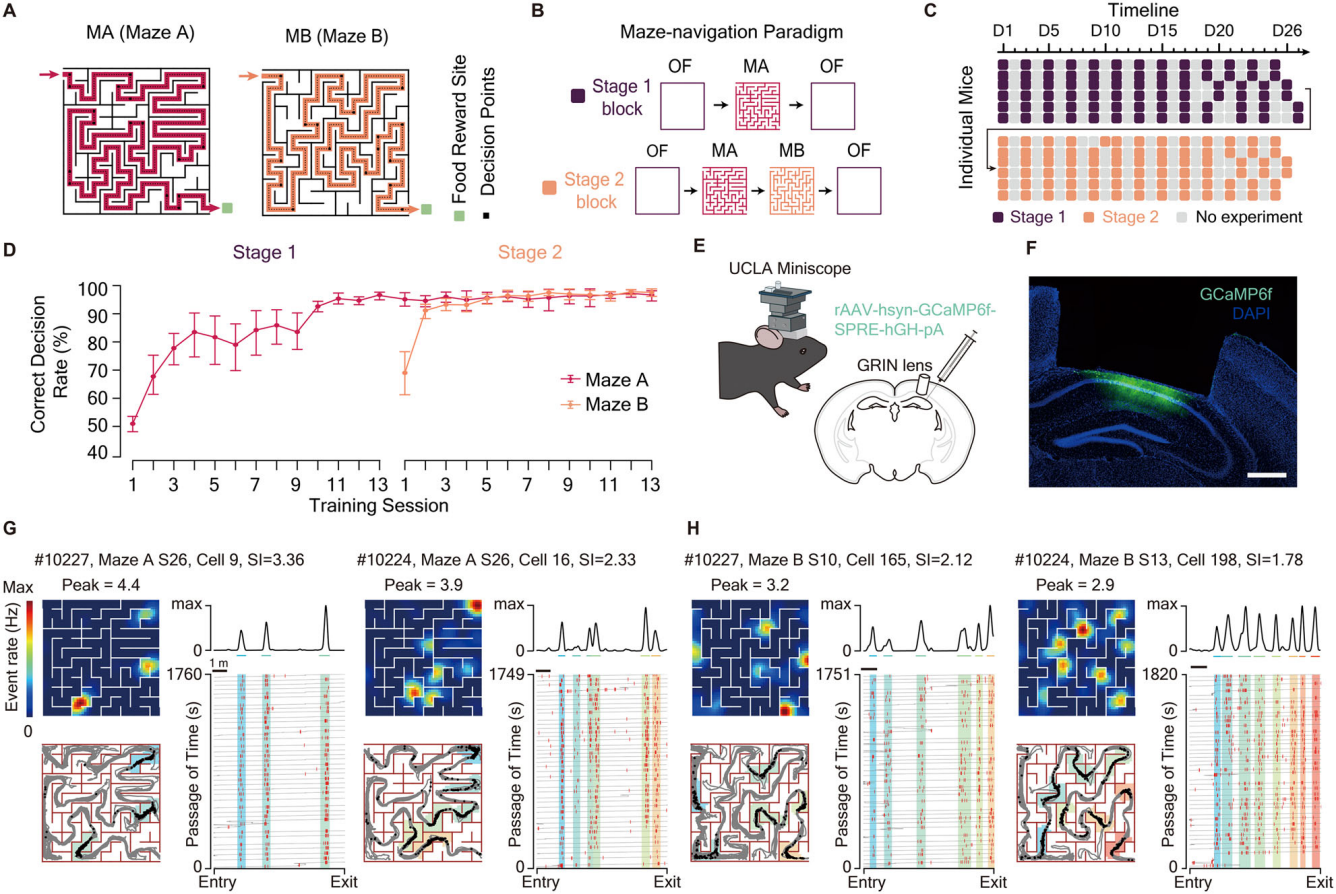
Long‐term one‐photon calcium imaging during training in the maze navigation paradigm. A) Configuration of Maze A (left) and Maze B (right). Red and orange lines display the linearized correct track for Maze A (8.88 m) and Maze B (8.08 m). B) Structure of training blocks in Stage 1 (top) and Stage 2 (bottom). MA: Maze A, MB: Maze B. OF: Open Field. C) Experimental timeline. Each horizontal line represents a mouse. 13 training blocks were conducted over 25–27 days in each stage. Colored squares denote training blocks; gray squares indicate days without experiments. D: Day. D) Behavioral performance measured as lap‐averaged correct‐decision rate, showing significant improvement across training. Two‐sided paired t‐test, n = 6 mice: Maze A Stage 1 S1 (50.8 ± 4.6%) versus Stage 2 S13 (97.2 ± 1.8%), P = 6 × 10^−6^; Maze B Stage 2 S1 (79.4 ± 8.4%) versus Stage 2 S13 (96.8 ± 2.2%), P=0.005. Error bars, 95% confidence intervals. E) Schematic of surgery and UCLA Miniscope setup. F) Coronal brain section showing AAV‐GCaMP6f expression in dorsal CA1. Scale bar: 500 µm. G,H) Example multi‐field place cells recorded in Maze A (G) and Maze B (H). Each cell is entitled with mouse ID, environment, recorded session (S), cell index, and spatial information (SI; unit: bits/spike). For each cell: **top left**, event rate map (Peak: peak event rate in Hz); **bottom left**, spatial distribution of calcium events (black dots) and mouse trajectories (gray lines); **top right**, linearized rate map along the correct track; **bottom right**, raster‐like plot with calcium events (red bars) aligned to linearized mouse trajectories (gray lines) along the correct track. Shaded areas of the same color mark the ranges of the same place fields. Calcium activities on incorrect tracks and backward movements are excluded.

Rodents instinctively excel in navigating within complex environments akin to the subterranean tunnels in where they naturally dwell. This ability inspired our design of the maze‐navigation paradigm (MNP) using two complex mazes, Maze A (MA) and Maze B (MB). Each maze incorporates 17 decision points along an 8‐ to 9‐meter‐long *correct path* connecting the entry and the exit (**Figure** **1**A). The MNP task was separated into two stages. Each stage lasted ≈26 days and comprised 13 blocks (Figure 1B), with several days of rest between the stages. A block in Stage 1 consisted of two 30‐min sessions in a familiar open field (OF) and one session in Maze A. In a block of Stage 2, an additional session in Maze B was introduced after the Maze‐A session (Figure 1C). Additionally, mice were trained to navigate back‐and‐forth in the Maze A (reversed‐maze paradigm, RMP) and a hairpin maze without decision points (hairpin‐maze paradigm, HMP) to guarantee that our findings are consistent across distinct movement directions and are irrelevant to decision‐making processes (Figure S1A, Supporting Information). Both paradigms were conducted over at least 7 days (Figure S1B, Supporting Information).

With only an average of 1983.2 ± 763.4 (mean ± std.) seconds (Figure S1C, Supporting Information) and an almost chance‐level correct‐decision rate of 43.0 ± 1.8% at decision points (Figure 1D. Figure S1G, Supporting Information) to locate the exit in the very first lap, mice displayed significant spatial learning both within the first session (Figure S1F,G, Supporting Information) and across the first 4 sessions (Maze A, Stage 1, n = 6 mice. Lap‐average navigation time: S1, 921.5 ± 418.3 s; S4, 91.7 ± 95.7 s. Two‐sided paired t‐test, P = 0.002. Figure S1C (Supporting Information). Lap‐average correct‐decision rate: S1, 50.8 ± 4.6 %; S4, 84.0 ± 10.9 %. P = 0.0007. Figure 1D. Behavioral progress measured by either indicator exceeds 70%, Figure S1D (Supporting Information). Upon familiarization with Maze A, mice underwent Stage 2 training in Maze B, exhibiting similar learning patterns (Figure 1D; Figure S1C,D, Supporting Information). Ultimately, mice not only mastered the maze exit route but also optimized their path (Shortest lap for each mice: Maze A, 6.31 ± 0.07 m; Maze B, 5.68 ± 0.04 m), achieving high accuracy in spatial decisions (Last session for each mice: Maze A, 97.2 ± 1.8%; Maze B: 96.8 ± 2.2%, Figure 1D) and mean speed (Fastest lap for each mice: Maze A, 43.3 ± 1.7 cm s^−1^; Maze B, 43.7 ± 1.5 cm s^−1^, Figure S1E, Supporting Information), showcasing robust and precise spatial learning.

### 2.2 One‐Photon Calcium Imaging of dCA1 Activity

To investigate the long‐term dynamics of hippocampal spatial representation, we employed the UCLA Miniscope (Figure 1E) to record neural activity in the dCA1 region of freely moving mice. We injected rAAV‐hsyn‐GCaMP6f‐SPRE‐hGH‐pA virus to express the calcium indicator GCaMP6f (n = 6 mice, Figure 1F), yielding 492 ± 161 (Open Field, n = 311 sessions), 439 ± 149 (Maze A, n = 155 sessions), and 438 ± 136 (Maze B, n = 78 sessions) regions of interest (ROIs) per session. The number of ROIs remains statistically invariant over recordings (One‐way ANOVA, P > 0.99 for all environments). Over 90% of active ROIs per session in both mazes (Maze A: 94.0 ± 8.9%, n = 154 sessions from 6 mice; Maze B: 93.6 ± 8.2%, n = 78 sessions from 6 mice; Figure S2A,B, Supporting Information) were identified as place cells (Figure 1G,H; Figure S3, Supporting Information), a significantly higher proportion than observed in the open field (78.8 ± 8.8%, n = 154 sessions. Two‐sided paired t‐test, OF versus MA: P = 1 × 10^−68^; OF versus MB: P = 2 × 10^−25^. Figure S2A,B, Supporting Information).

Moreover, the within‐session stability of these place cells showed marked enhancement in all environments throughout training (Figure S2C–E, Supporting Information), alongside a significant reduction of the decoding errors in both Maze A and B (Figure S2F–H, Supporting Information). In contrast, decoding errors remain unchanged in the open field (Figure S2I,J, Supporting Information). This suggests an improved spatial coding throughout maze learning.

### 2.3 Canonical Statistical Structures of Multi‐Scale and Multi‐Field Spatial Maps in Complex Mazes

During maze‐navigation paradigm (MNP) training in Maze A, most place cells in these mazes exhibited multiple fields, averaging 6.7 ± 3.7 fields per cell in Maze A and 6.1 ± 3.3 in Maze B. The reversed‐maze paradigm (RMP) revealed that place cells in complex mazes are highly directional, with only 19.6 ± 5.6% of bi‐directional place fields (chance level: 14.8 ± 4.9%, Paired t‐test, P = 1.3 × 10^−13^), suggesting that the spatial maps encoding each direction were nearly distinct. Place fields in the hairpin maze (HP) also displayed high directionality (17.0 ± 5.2% of bi‐directional fields; chance level: 12.8 ± 4.4%, Paired t‐test, P = 3.9 × 10^−11^). All spatial maps displayed extensive multi‐field coding, with exceeding 90% place cells exhibiting multiple fields in all paradigms (MA forward: 93.6 ± 3.5%; MA backward: 94.0 ± 3.1%; HP forward: 95.3 ± 2.7%; HP backward: 94.1 ± 3.6%) and an average field number well above one (MA forward: 6.4 ± 1.7; MA backward: 7.3 ± 1.8; Figure S4A–E, Supporting Information; HP forward: 8.6 ± 2.6, HP backward: 8.0 ± 2.2; Figure S4F–K, Supporting Information). For clarity, place cells’ populational activities detected during training in Mazes A and B in the maze‐navigation paradigm, as well as during forward and backward movements in the reversed‐maze paradigm and hairpin‐maze paradigm, are collectively referred to as six spatial maps: MA, MB, MAf, MAb, HPf, and HPb.

To ensure that our multi‐field spatial maps were not artifacts produced by flawed field identification criteria, we corroborated that these maps conformed to canonical statistical structures for field sizes^[^ ^57^ ^]^ and field numbers.^[^ ^8^, ^56^ ^]^ Specifically, field sizes across six spatial maps adhered to skewed distributions^[^ ^57^ ^]^ (Figure S5, Supporting Information). Moreover, the distribution of field counts per neuron throughout all goal‐directed navigation paradigms was precisely captured by a negative binomial distribution (Figure S6, Supporting Information), dovetailing with the Gamma‐Poisson hypothesis.^[^ ^8^, ^56^ ^]^ Collectively, these results suggest that the multi‐scale and multi‐field representations observed in all spatial maps display canonical statistical structures mirroring those identified in the long linear tracks.

### 2.4 Tracking Place Fields across 7 to 26 Sessions

The prevalence of multi‐field place cells in our dataset yielded an average of several thousand place fields per session (mean ± std.; MA: 2761 ± 1015; MB: 2144 ± 1014; MAf: 2188 ± 759; MAb: 2306 ± 672; HPf: 2629 ± 690; HPb: 2381 ± 750 place fields per session), benefiting the identification statistical rules underlying their temporal evolution. Initially, active ROIs within a given FOV were tracked and registered across sessions using CellReg.^[^ ^64^ ^]^ To further increase the number of continuously tracked neuron pieces while maintaining registration accuracy comparable to CellReg, we developed a re‐matching strategy (Figure S7, Supporting Information). Functional consistency of the registered neurons was confirmed via cross‐session decoding with a Naïve Bayes classifier: decoding loss decreased with training progression and increased with session interval (Figure S8, Supporting Information), in agreement with previous decoding studies^[^ ^8^ ^]^ and cross‐session spatial map correlation^[^ ^9^, ^10^, ^65^ ^]^ results and suggesting that our data support empirical stabilizing dynamics of hippocampal place codes. Also, to further validate long‐term registration stability, we examined spatial footprint (SFP) displacement vector fields of CellReg‐based re‐matching strategy registered cells (Figure S9A, Supporting Information), showing no obvious non‐rigid transformation, with stable relative neuron positions. Inter‐session image correlation matrix also confirmed stable recording across training days (Figure S9B,C, Supporting Information). We performed fixed baseplate and manual adjustments referred to possible landmarks (e.g., blood vessels) to minimize possible *z*‐axis drift. Histogram‐based intensity correlation matrix further confirmed stable imaging plane (Figure S9D and E, Supporting Information). Quantitatively, registered neuron pairs exhibited significantly higher spatial footprint correlations (Figure S9F and G, Supporting Information) and smaller centroid distances (Figure S9H,I, Supporting Information) compared to nearest neighbors (excluding matched pairs), supporting robust CellReg‐based registration combined with manual verification. The results of cell tracking and place‐field registration are summarized in Table S1 (Supporting Information).

**Figure 2:**
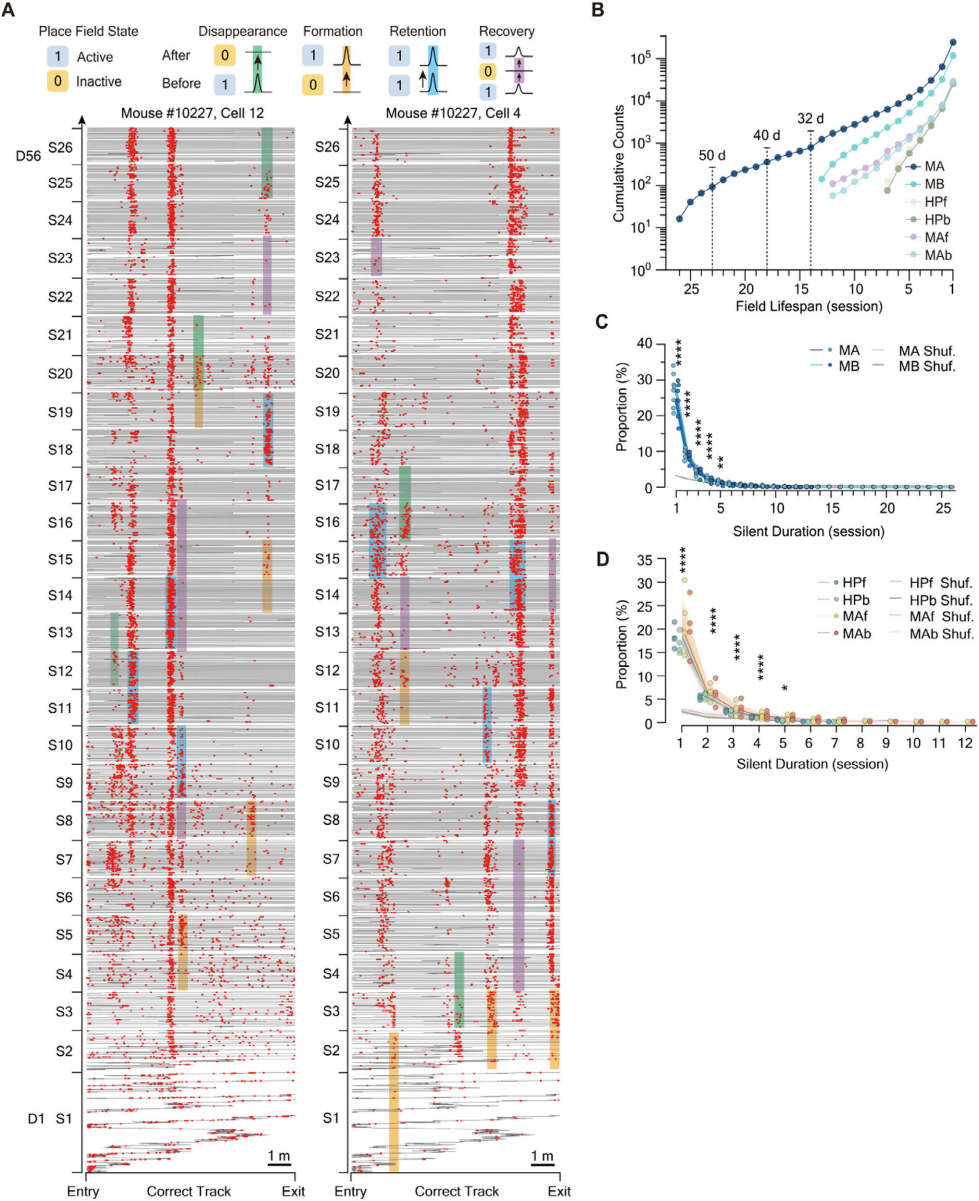
Multi‐field place cells undergo complex long‐term dynamics characterized by three elementary evolutionary events. A) Two example place cells tracked over 26 sessions (56 days). Three elementary evolutionary events—disappearance, formation, and retention—are highlighted with green, yellow, and blue shading, respectively. We only selectively marked several evolutionary events. Purple shading indicates continuous dormancy periods of fields that later recovered. Only a proportion of representative events are marked. Red bars, calcium events; gray lines, mouse trajectories on the correct track. Sessions are labeled S1–S26 (Stage 2 S13). Calcium activity on incorrect tracks and during backward movements was excluded. Additional examples from other mice and environments are shown in Figure S10 (Supporting Information). B) Lifespans of all tracked place fields, defined as the longest period of continuous activity, pooled across six mice. Lifespans of 14, 18, and 23 sessions correspond to ≈32, 40, and 50 days, respectively. C,D) Distribution of silent durations for all field‐recovery events in spatial maps MA, MB (C), MAf, MAb, HPf, and HPb (D). Shuffled levels were determined separately for each spatial map by randomly registering fields across random cell pairs, reflecting recovery levels introduced by the field registration algorithm. Color bands of shuffled lines: 95% Confidence Interval. P values were obtained using two‐sided paired t‐tests (C: n = 14 spatial maps from 6 mice; D: n = 16 spatial maps from 4 mice). MA: Maze A, MNP Task; MB: Maze B MNP Task; MAf: Maze A forward, RMP Task; MAb: Maze A backward, RMP Task; HPf: Hairpin Maze forward, HMP Task; HPb: Hairpin Maze backward, HMP Task. Significance levels: ^***^ *P* < 0.001; ^****^ *P* < 0.0001.

Several representative long‐term tracked neurons from different spatial maps are shown (**Figure** **2**A; Figure S10, Supporting Information), each exhibiting complex evolutionary dynamics involving multiple place fields. Given the prevalence of multi‐field place cells in our dataset, we further registered individual place fields of each neuron across sessions based on their cross‐session overlap (Figure S11, Supporting Information). Because different spatial maps from the same mouse comprise distinct sets of place codes, their coding activities were tracked separately. Among all registered place fields, a noteworthy subset exhibited remarkable longevity (Figure 2A; Figure S10, Supporting Information), with 352 fields continuously remaining active for at least 40 days (18 sessions; n = 4 mice) and 91 fields continuously persisting for a minimum of 50 days (23 sessions; n = 4 mice; Figure 2B) in Maze A. These long‐lasting fields can coexist with other place fields with comparatively shorter lifespans, reflecting a heterogeneity in the evolutionary trajectories of place fields within the same neuron (Figure 2A; Figure S10, Supporting Information). With this observed asynchronization, the evolution of multi‐field place cells is complex (Figure 2A; Figure S10, Supporting Information). To this end, we simplified this complexity in three aspects: 1) We initially explored the evolutionary trajectory of neural activity independently for each place field, a process termed as *single‐field evolution*, while temporarily setting aside potential inter‐field relationships; 2) We binarized the *observable states* of a registered place field at each session as either active (denoted as 1) or inactive (denoted as 0), its observable states across *t* sessions can thus be expressed as a binary sequences, hereafter referred to as the *field‐state sequence*. Note that all field‐state sequences begin with 1, corresponding to the session in which the field was formed (Figure S11A, Supporting Information). 3) We defined three basic evolutionary events on a single‐field basis across consecutive sessions: *disappearance*, *formation*, and *retention* of place fields, corresponding to field‐state pairs [10] ([active‐inactive]), [01] ([inactive‐active]), and [11] ([active‐active]), respectively (Figure 2A).

To validate the accuracy of our extracted evolutionary events, we analyzed the event rate changes within the place fields undergoing these three evolutionary events across consecutive sessions, observing significant decreases, increases, and mild fluctuations, respectively (Figure S12A,B, Supporting Information). These events were not systematically and consistently associated with changes in neuronal peak calcium traces (peak ΔF/F; Figure S12C,F, Supporting Information), cellular noise levels (Figure S12D, Supporting Information), or signal‐to‐noise ratios (Figure S12E, Supporting Information).

Combinations of these evolutionary events result in complicated evolutionary trajectories of each field, while one of these combinations—the recovery of place fields (i.e., field‐state sequence [.10…01.])—is of particular interest. Notably, 15–45% weakened or even disappeared place fields have a significantly greater than chance probability of recovery within the next five sessions (cumulative probability subtracted with chance level: MA: 27.3 ± 4.0%; MB:24.9 ± 4.0%; MAf: 29.4 ± 9.2%; MAb: 28.3 ± 9.4%; HPf: 25.7 ± 2.6%; HPb: 25.2 ± 2.0%; Figure 2C,D). This ubiquity of recovery events highlights one of the critical components of single‐field evolution.

### 2.5 Statistical Analysis Revealed History Dependence of Single‐Field Evolution

**Figure 3:**
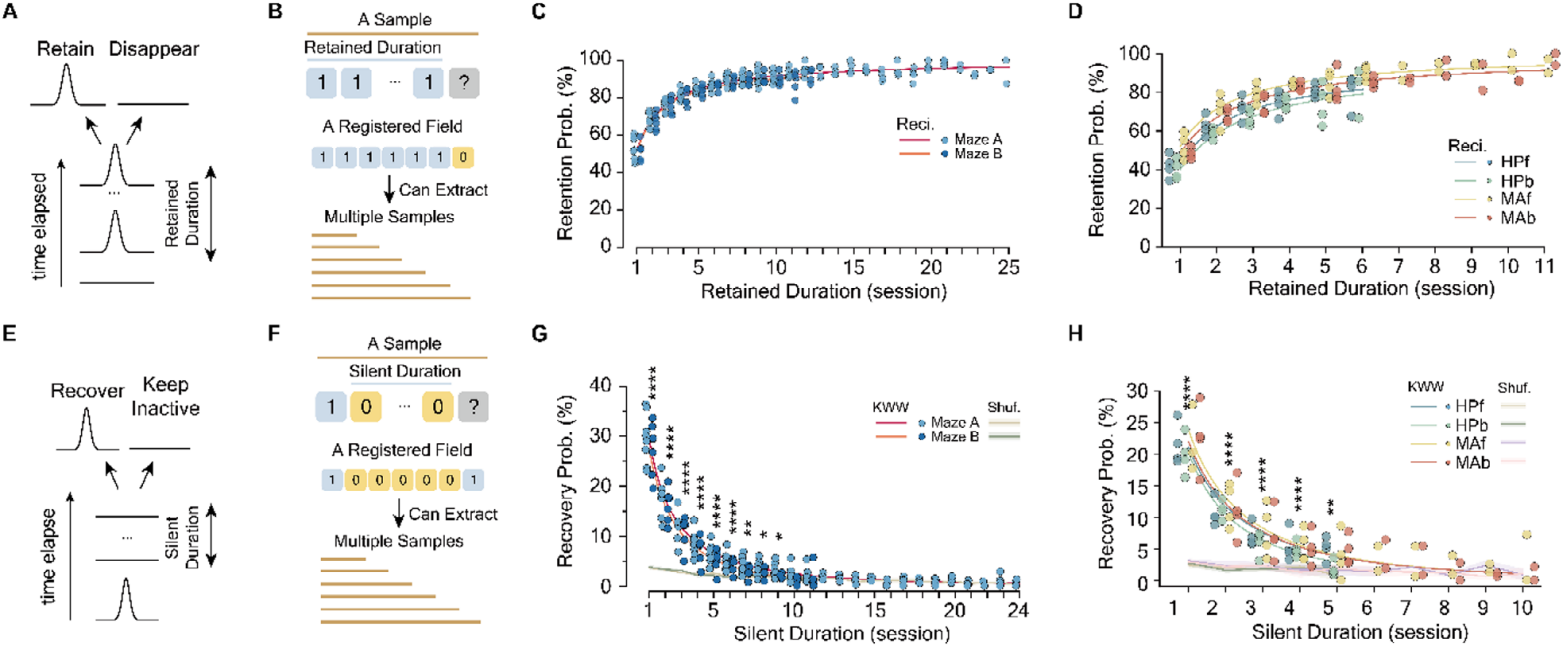
Statistical analysis revealed a robust history dependency of single‐field evolution. A) Schematic of field‐retention probability. B) Preparation of field samples for field‐retention probability. A field‐state sequence such as [1,1,1,1,1,0] can contribute multiple samples: a field retained for 5 consecutive sessions has also effectively been retained for 4, 3, 2, and 1 sessions at earlier points. C,D) Field‐retention probability of spatial maps MA and MB (C), MAf, MAb, HPf, and HPb (D). Field retention probability increases as the duration of retention extends, a trend captured by reciprocal functions (lines). E) Schematic of field‐recovery probability. F) Preparation of field samples for field‐recovery probability. A field‐state sequence such as [1,0,0,0,0,0,1], may also be included as a sample multiple times; a field that has been inactive for 5 sessions has also been inactive for 4, 3, 2, and 1 sessions at earlier points. G,H) Field‐retention probability of spatial maps MA and MB (G), MAf, MAb, HPf, and HPb (H). Field‐recovery probability decreases as the silent duration extends, a trend captured by Kohlrausch‐Williams‐Watts (KWW) functions (lines). Data points with fewer than 5 samples were excluded. Shuffled levels were determined separately for each spatial map by randomly registering fields across random cell pairs, reflecting recovery levels introduced by the field registration algorithm. Color bands of shuffled lines: 95% Confidence Interval. P values were obtained using two‐sided paired t‐tests (C: n = 14 spatial maps from 6 mice; D: n = 16 spatial maps from 4 mice). Significance levels: ^*^ *P* < 0.05; ^**^ *P* < 0.01, ^***^ *P* < 0.001; ^****^ *P* < 0.0001.

The disappearance and recovery of place fields fundamentally reflect that these fields can transition between active and inactive states, prompting a central inquiry: what factors govern the field‐state transition? To investigate this question, we employed classical probabilistic analysis. We first examined the conditional *field‐retention probability*, which is computed as the likelihood that a place field stays active in the next session given a specific *retention duration*, which is the duration for which a place field has remained continuously active (**Figure** **3**A,B). Our analysis revealed a clear, temporally increasing trend in field‐retention probability. Specifically, we observed a significant increase in this probability as the retained duration extended, a phenomenon consistent across all six spatial maps (from R_t_ = 1 to 8, MA: 52.5 ± 5.9% to 89.8 ± 2.8%, two‐sided paired t test, P = 5 × 10^−7^, df = 7; MB: 51.6 ± 4.4% to 89.2 ± 2.1%, P = 1 × 10^−5^, df = 5; Figure 3C. From R_t_ = 1 to 6, MAf: 55.1 ± 4.9% to 90.8 ± 2.7%, P = 0.002; MAb: 52.4 ± 4.3% to 89.4 ± 4.2%, P = 0.003; HPf: 46.8 ± 5.3% to 82.7 ± 8.7%, P = 0.012; HPb: 45.0 ± 5.0% to 77.3 ± 3.3%, P = 0.003; df = 3; Figure 3D), indicating a property that is context‐ and decision‐making‐irrelevant (Maze A&B vs hairpin maze). This increasing field‐retention probability can be aptly and empirically described by the reciprocal function (Figure 3C,D). Moreover, place fields located in different subregions along the maze tracks exhibited analogous trends and probability levels, suggesting a position‐irrelevant characteristic (Figure S13, Supporting Information). To ascertain whether the retention probability is related to novelty, we separately computed it (Figure S14A–F, Supporting Information) for place fields that formed in different sessions. Most comparisons yielded no significant differences (Figure S14F, Supporting Information), indicating that the retention probability represents novelty‐irrelevant characteristics of single‐field evolution. To test whether neurons with different signal‐to‐noise ratios (SNR) disproportionately contribute to retention probability, we computed retention probability for registered neurons stratified by average SNR levels (Figure S15A,B, Supporting Information). The results indicate that retention probability is largely independent of neuronal SNR (Figure S15C,D, Supporting Information). Furthermore, it is worth noting that for place fields that were established during Stage 1 in Maze A, the field‐retention probability continued to accrue during Stage 2, remaining unaffected by the introduction of Maze B (Figure S14A, Supporting Information). This result suggested that evolutions of distinct spatial maps are mostly independent.

Subsequent analysis of *field‐recovery probability*—the likelihood that an inactive place field regains activity—revealed a similar dependence on the continuous inactive duration, hereafter referred to as the *silent duration* (Figure 3E,F). Remarkably, this probability exhibited a significant decline with prolonged silent duration (from S_t_ = 1 to 8, MA: 29.7 ± 4.7% to 3.5 ± 1.4%, two‐sided paired t test, P = 9 × 10^−7^, df = 7; MB: 27.5 ± 5.1% to 2.8 ± 1.0%, P = 7 × 10^−5^, df = 5; Figure 3G). Despite this decline, recovery probabilities remained significantly above chance levels for up to eight sessions. Similar patterns were observed across all other spatial maps (from S_t_ = 1 to 5, MAf: 26.3 ± 5.2% to 4.6 ± 2.7%, P = 0.002; MAb: 25.4 ± 6.1% to 4.0 ± 1.6%, P = 0.007; HPf: 23.8 ± 3.2% to 5.6 ± 1.1%, P = 0.002; MAb: 22.4 ± 2.1% to 3.1 ± 0.7%, P = 0.0004; df = 3; Figure 3H), suggesting a similar context‐ and decision‐making‐irrelevant property. The observed declining trend in recovery probability is aptly and empirically described by the Kohlrausch‐Williams‐Watts (KWW) function, an exponential function with a lower decaying rate (Figure 3G,H). While recovery probability was largely unaffected across a broad range of neuronal SNR levels (5–20), registered neurons with extremely high average SNR (≥ 20) exhibited significantly lower recovery probability (Figure S15E,F, Supporting Information), suggesting potentially higher tracking accuracy. Nevertheless, the recovery probability of these high‐SNR neurons still showed a similar dependence on silent duration, supporting the generality of this phenomenon across neuronal ensembles (Figure S15E,F, Supporting Information).

For clarity, we have designated these two probabilities as the *single‐field evolution rule (SFER) in the preliminary sense*: Place fields with longer durations of activity are more likely to persist in the next session, while those with longer periods of inactivity are less likely to recover.

### 2.6 Sequential Learning Models Revealed General Temporal Patterns in Determining Single‐Field Evolution

**Figure 4:**
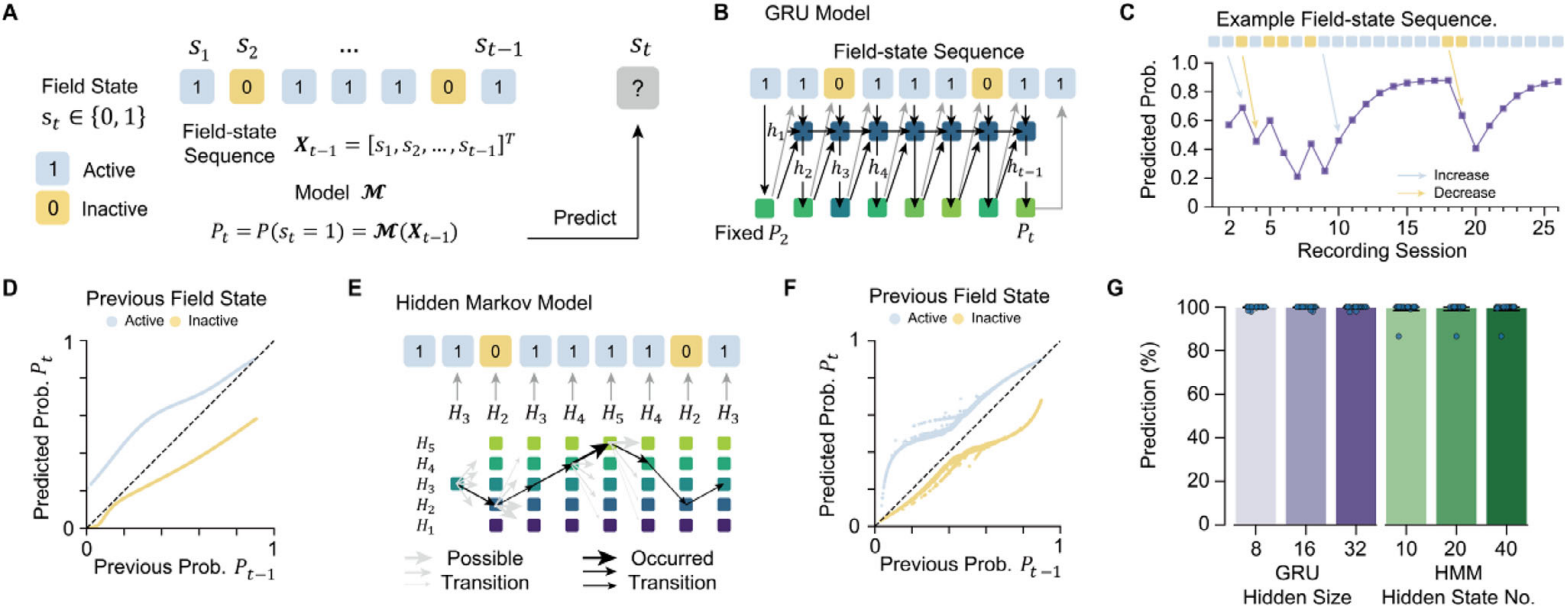
Sequential learning models revealed opposite roles of active and inactive states. A) A general paradigm for modeling single‐field evolution. B) Architecture of the Gated Recurrent Unit (GRU) model (Methods, Model V). Dark blue squares represent the hidden layer, green squares denote the output linear layer, black arrows indicate the inputs, and grey arrows represent the output probability that determines the expression of the place field in the subsequent session. C) The GRU model trained with the data spatial map MA from mouse #10227. The predicted *P_t_* from an example field‐state sequence demonstrates variation closely linked to the previous field state. D) The same model as in (C) reveals the opposing roles of active and inactive states in the update of *P_t_*: a previous active state enhances *P*_*t* − 1_ (blue line), while a previous inactive state weakens *P*_*t* − 1_ (yellow line). E) Illustrative schematic of a hidden Markov model (HMM) with 5 hidden states. The thickness of the arrows represents the probability of transition. F) HMM trained with spatial map MA from mouse #10227, shows a similar separated pattern depending on the previous field state. G) Results from 4 mice across all six spatial maps demonstrate that most predictions follow the previous field state‐dependent update of *P_t_*.

While the SFER in the preliminary sense is notably robust, one might contend that it may arise from a more overarching pattern that fundamentally governs single‐field evolution. Therefore, an in‐depth exploration of the broader patterns underlying single‐field evolution is imperative. We next sought to identify a temporal pattern that predicts the probability that a place field with an age of *t* − 1 sessions will become active in the subsequent session *t* (denoted as *P_t_*), based on its entire activity history since formation. Recalling that we can represent history activity of a place field as a binary *history field‐state sequence* with a length of *t* − 1. The central task of our analysis was therefore to select an appropriate model capable of capturing the temporal structure of history field‐state sequences, thereby enabling accurate prediction of *P_t_* (**Figure** **4**A).

To effectively capture temporal dependencies within history field‐state sequences, models with sequential learning capacities, such as the recurrent neural networks (RNN) and hidden Markov models (HMM) are suitable. RNNs are a class of neural networks with hidden layers that function as the internal memory, allowing past information to be retained and used to model temporal structure beyond solely current *observable states* (i.e., active or inactive state). We first customized a specific type of RNN model, the gated recurrent unit (GRU) network (Figure 4B), which posits that, in addition to observable states, individual place fields possess *hidden states* corresponding to distinct reappearance probability *P_t_*. The hidden layer of the GRU updates these hidden states based on input from the observable field state at session *t* − 1, subsequently outputting the predicted *P_t_* to generate the current observable field state at session *t* (Figure 4B). A heuristic example field‐state sequence illustrates the GRU‐predicted *P_t_*, revealing strong and intuitive links with the observable states: a previous active state of a place field enhances its *P_t_* ​, making it more likely to be active in the subsequent session, while a previous inactive state diminishes it (Figure 4C). More specifically, the iterative relationship between the model‐predicted *P*_*t* − 1_ and *P_t_* is governed by two monotonic functions—one that recursively increases *P_t_* (with the curve consistently above the diagonal) and another that reduces *P_t_* (below the diagonal) (Figure 4D).

We cross‐validate this finding on HMMs that are based on different assumptions. Briefly, the HMM also assumes the presence of stability‐related hidden states of place fields but differs with a GRU model in two aspects: 1) these hidden states correspond to explicitly determined levels of field's stability, whereas in a GRU model the hidden states are fitted by data and may have complex mapping relationships with field's stability. 2) The number of hidden states is finite for a HMM, whereas the hidden state in a GRU model is much closer to a continuous variable, if there's an adequate amount of hidden unit. 3) The transitions of hidden states are stochastic (Figure 4E) rather than one‐to‐one mapping in the GRU (Figure 4D); a hidden state with the greatest stability has the likelihood, albeit typically low, to directly switch to the one with the lowest stability (Figure 4E). Although it is more randomly displayed due to the stochastic process HMM involves, the mapping from the model‐predicted *P*_*t* − 1_ to *P_t_* is also separated by the diagonal based on the previous observable field states (Figure 4F). In fact, most changes in *P_t_* in either model are reliably governed by these opposite driving forces (GRU and HMM > 99.3% of 152 0154 times of prediction. n = 4 mice × 6 spatial maps = 24. Figure 4G), suggesting it is a necessary feature for single‐field evolution. For clarity, we refer to the opposing roles of observable field states as the *SFER in the general sense*, which can be recursively defined as:

(1) $$P_{t}=s_{t-1}f\left(P_{t-1}\right)+\left(1-s_{t-1}\right)g\left(P_{t-1}\right)$$

where *s*_*t* − 1_ represents the observable field state in the previous session, and *f* and *g* are functions that incrementally or decrementally update *P*_*t* − 1_, respectively. We empirically selected linear (Figure S16A, Supporting Information), logistic (Figure S16B, Supporting Information), quadratic (Figure S16C, Supporting Information), and cubic (Figure S16D, Supporting Information) to fit *f* and *g* (see Experimental Section).

### 2.7 Stochastic Discrete Dynamical Systems featured by SFER Necessarily Display Dual‐Fate Dynamic

While population‐level stabilization of hippocampal place codes has long been considered novelty‐dependent,^[^ ^12^, ^31^ ^]^ we found that SFER is novelty‐irrelevant: place fields formed in different sessions exhibited similar initial retention probabilities (*P*_2_) (Figure S14, Supporting Information). This raises the question of how novelty‐irrelevant single‐field evolution can give rise to novelty‐relevant population‐level stabilization. To bridge this gap, we conducted numerical simulations by establishing a *stochastic discrete dynamical system* defined by SFER. This framework has three key components: the dynamical system, discreteness, and stochasticity. A *dynamical system* is a mathematical framework that describes how the state of something—in this case, whether a place field is active or inactive—changes over time according to specific rules. The *discrete* property means that these changes occur in steps rather than continuously; here, each step corresponds to one recording session. Finally, *stochasticity* means that the governing rule introduces randomness, so the system's evolution at each step is probabilistic rather than fully deterministic.

**Figure 5:**
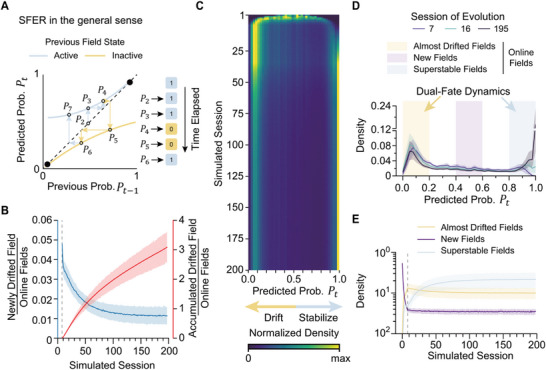
Stochastic discrete dynamical systems defined by SFER capture empirical population‐level drift dynamics. A) Illustration of the stochastic discrete dynamic system defined by the single‐field evolution rule. Quadratic functions are fitted to approximate the *f* and *g* in formula (1), governing the system's behavior: incremental update (blue) and decremental update (yellow). Filled dots: the fixed points of each function, referred to as attractor‐like structures. Hollow dots: *P_t_* at each step. The functions and fixed points are only plotted for visualization here. B) Dynamics of drift rate defined by the proportion of newly drifted fields (blue line) and accumulated drifted fields (red line). n = 6 mice × 6 spatial maps × 5 simulations = 180. Dashed line: the session with gate activation. C) Simulation of 10 000 fields over 200 sessions. The probability density function (40 bins within the interval of (0, 1]) for all online fields in each simulated session is visualized as a heatmap. Probability density is normalized for each session for clarity. D) Probability density function of *P_t_* in three selected simulated sessions (session 7, 16, and 195). Background colored shadows: the three peaks of the *P_t_* distribution, suggesting dual‐fate dynamics. (E) Peak density of the three field clusters observed in (C) and (D) evolves throughout the simulation. n = 6 mice × 6 spatial maps = 36 for (C–E). The dashed line: the session with gate activation. Error bands: 95% confidence interval.

Specifically, each place field has an observable state *s_t_* and a hidden state *P_t_*. For any newly formed place field, the initial conditions are equally set as *s*_1_ = 1 and *P*_2_. While *P*_*t* − 1_ represents the probability that *s*_*t* − 1_ = 1, SFER specifies how *s*_*t* − 1_ updates *P_t_*, thereby allowing the evolution of a place field to be simulated iteratively (Equation (1), **Figure** **5**A). By simulating 10 000 place fields over at least 200 sessions, we could examine the collective behavior of a population of place fields when each evolves independently according to SFER. We defined “drift” as the action of reducing *P_t_*, whereas “stabilize” increases it. Given our prior observations that fields remaining silent for 5 to 8 sessions have only a chance‐level likelihood of recovery (Figures 2C,D and 4G,H), we introduced a gate mechanism—a threshold of 8 sessions—to permanently withdraw inactive fields from simulation. Fields designated as permanently silent were labeled as *drifted fields*, while the remaining fields were termed *online fields*.

A significant number of drifted fields emerged during single‐field evolution (**Figure** **5**B), highlighting notable drift at the single‐field level. However, the accumulation rate of drifted fields decreases over time, ultimately plateauing at ≈1% of all online fields per session (Figure 5B), indicating a balance condition. The distribution of *P_t_* among online fields reveals three major peaks: 1) one centered around the initial probability, representing new fields; 2) A concentration in the low *P_t_* region, corresponding to *almost drifted fields*; and 3) a substantial proportion with high *P_t_*, indicating *super stable fields* (Figure 5C,D; Figure S17, Supporting Information). Before gate activation, the latter two peaks form and accumulate rapidly, accompanied by a sharp decline in the new‐field peak (Figure 5E). After gate activation, the new‐field peak reduces gradually, offsetting the permanent drift of online fields (Figure 5E). Meanwhile, the second group declines, aligning with the decelerated accumulation of drifted fields (Figure 5B), while the superstable‐field group gradually accumulates and eventually becomes dominant (Figure 5E). Consequently, the novelty‐related stabilization of place codes at the population level—characterized by initially high drift rates that later decline—emerges as a collective outcome of the novelty‐irrelevant single‐field evolution rule, provided that network‐level mechanisms maintain a stable total number of fields.

Fundamentally, the inherent dual‐fate dynamics of SFER arise from the properties of the two functions, *f* and *g*. These functions may intersect with the diagonal line (where the equality relation *P*_*t* − 1_ = *P_t_* always holds, Figure 5A), and the intersection points are known as *fixed point* (black dots, Figure 5A). For function *f*, without loss of generality, we consider the case in which a place field remains constantly active. In this scenario, its *P_t_* will iteratively converge toward the fixed point of *f*, yielding a super‐stable field. Conversely, for function *g*, if a place field remains inactive after formation, its *P_t_* will iteratively converge toward the fixed point of *g*, producing an almost‐drift field. These two fixed points act as competing attractors, ultimately partitioning place fields into two divergent pools. At the ensemble level, the observed novelty‐related representational stabilization reflects the competition between these two fixed points, gradually approaching a balance point.

### 2.8 SFER Outperforms Selected Factors in the Demonstration of Single‐Field Evolution

In addition to the SFER, several factors—such as the amount of active experience,^[^ ^9^ ^]^ the passage of time,^[^ ^7^, ^29^ ^]^ and intrinsic field properties (e.g., firing rate and within‐session stability)—potentially influence representational stability. To evaluate whether SFER plays a primary role in dictating single‐field evolution, it is essential to quantify the extent to which dCA1 representational evolution can be explained by factors beyond history dependence.

Nine elements that have been identified or are potentially influential in affecting dCA1 representational stability include: 1) behavioral progression, 2) the number of training sessions, 3) intervals between sessions, 4) active experiential time (velocity ≥ 2.5 cm s^−1^) mice spent within the field, 5) peak event rate within the field, 6) within‐field stability within a session, 7) the first lap in which a field appears within a session, 8) maximal calcium transient, and 9) lap‐by‐lap fluctuation of field center (Figure S18A, Supporting Information). Elements 1 to 3 represent global factors related to the overall state of the animal in each session, while elements 4 to 9 focus on field‐specific information.

**Figure 6:**
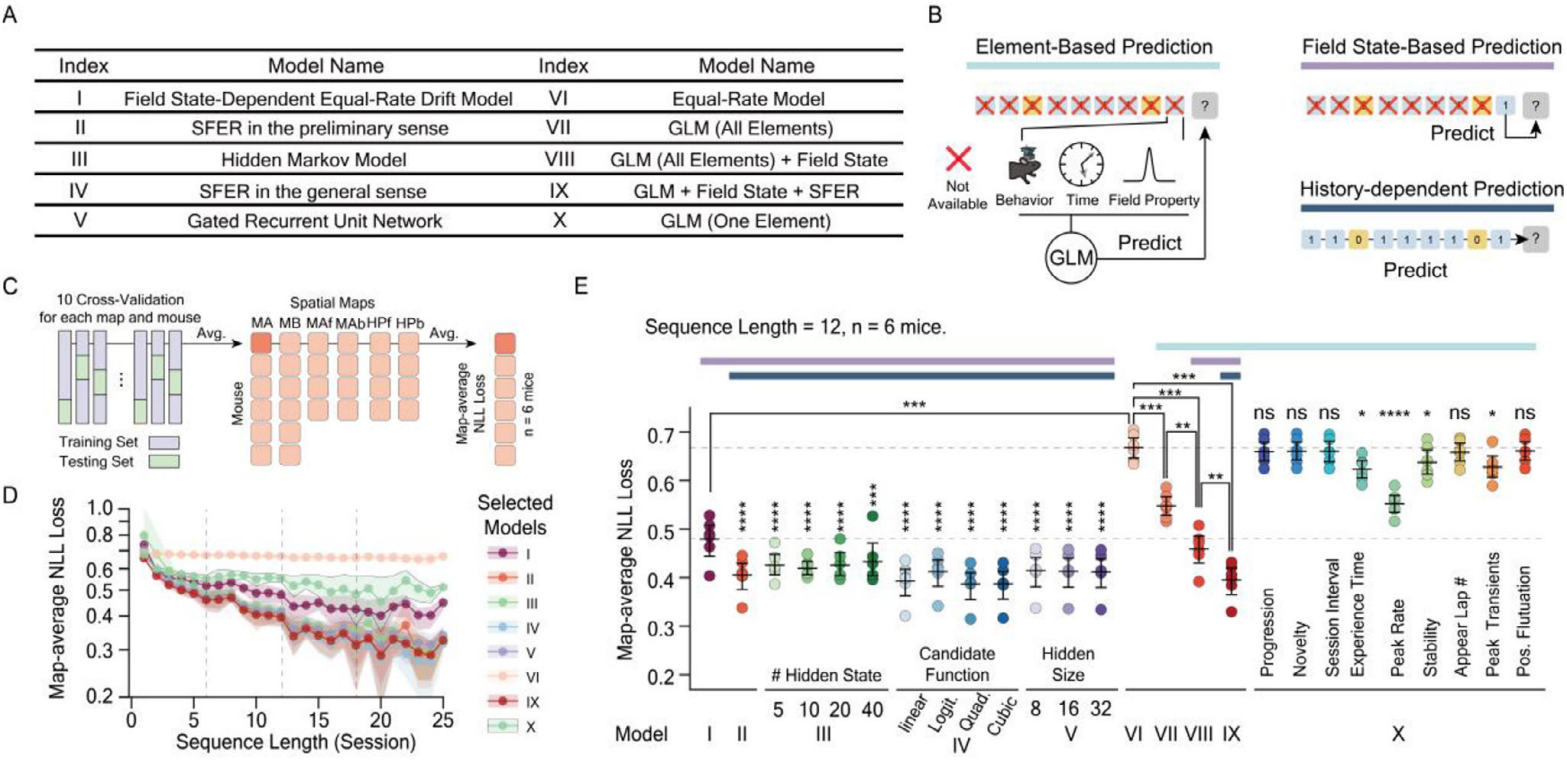
SFER performs better in demonstrating single‐field evolution. A) Names and indices of the models used for comparison. B) Three primary strategies employed by all models: 1) Element‐based models predict *P_t_* based on information beyond field states from the previous session. 2) Field state‐based models predict *P_t_* according to the field state from the previous session. History‐dependent models predict *P_t_* based on the entire preceding field‐state sequence. C) Schematic for the decoding. Briefly, 80% of field‐state sequences were subject to train each model, and testing was conducted over the remaining 20% part. Training set was randomly selected for 10 times for cross‐validations. For each mouse, the average loss from cross‐validation was further averaged across distinct spatial maps and resulted in the map‐average loss. NLL: Negative Log‐likelihood. D) Map‐average NLL loss reduces when sequence length extends for 8 selected models (for Model III (HMM), hidden state is 10; For Model IV, candidate function is linear; For Model V, hidden size is 16; For Model X, the element is peak rate). Shaded error bars indicate 95% confidence intervals. E) Decoding losses of all models at a sequence length of 12. Abbreviations: Logit. = Logistic; Quad. = Quadratic. Stars above Models II to V indicate significance levels compared with Model I, while stars above Model X indicate significance levels compared with Model VI. Two‐sided paired t‐tests with Bonferroni correction were conducted for all statistical comparisons (plus Model I vs IX: P = 1.1 × 10^−3^), n = 6 mice. The top‐colored bands represent the decoding strategies (B) each model employs. Error bars: 95% Confidence Intervals. Significance levels: ns: *P* ≥ 0.005; ^*^: *P* < 0.005; ^**^ *P* < 0.001; ^***^ *P* < 1 × 10^−3^; ^****^ *P* < 1 × 10^−4^.

Subsequently, we investigated whether models relying on this non‐history information could achieve comparable effectiveness in demonstrating single‐field evolution. 10 model types (**Figure** **6**A; see Experimental Section) were considered to predict *P_t_* following element‐based, field‐state‐dependent, or history‐dependent decoding strategies (Figure 6B). Particularly, a generalized linear model (GLM) was constructed to predict *P_t_* ​ from either all elements (Model VII) or individual elements (Model X). The average loss across 10 cross‐validations was used (Figure 6C). Notably, the decoding losses of several representative models gradually decreased as the length of the field‐state sequence increased (Figure 6D). Compared to purely random drift (Model VI), the non‐SFER GLMs (Model VII) exhibited significantly enhanced predictive accuracy, suggesting that the aforementioned factors collectively contribute to single‐field stability across days (Figure 6E; Figure S18B,C, Supporting Information). GLMs utilizing a single element revealed that both peak firing rate and within‐session stability emerged as significant contributors (Figure 6E; Model X vs Model VI). However, these single‐element GLMs could not achieve a decoding loss as low as the models solely reliant on field state (Model I), let alone those depending on history information (Models II to V, Figure 6E). In fact, the integration of field state substantially improved decoding accuracy (Model VIII vs Model VII, Figure 6E), while incorporating SFER further enhanced model performance (Model IX). SFER models become more accurate as the length of field state sequences increases (lengths = 12, 6, and 18 in Figure 6E; Figure S18B,C, Supporting Information). These findings suggest that SFER serves as an effective approximation in elucidating single‐field evolution.

To rule out the possibility of overfitting, we performed additional cross–spatial map (Figure S19A–C, Supporting Information) and cross‐animal validations (Figure S19D–F, Supporting Information). In cross–spatial map validation, each model was trained on data from one spatial map (e.g., MA) and tested on another (e.g., MB; Figure S19A, Supporting Information). Since data from different spatial maps were collected independently without temporal overlap and neurons were tracked separately, sessions from one map can serve as a true testing set for another. The losses of representative models similarly decreased as sequence length increased (Figure S19B, Supporting Information). This validation revealed that the decoding accuracies of HMMs and SFER models with quadratic or cubic functions were not significantly different from controls (Model I; Figure S19C, Supporting Information), indicating overfitting. In contrast, other SFER‐featured models (GRU models, preliminary SFER, and SFER with linear or logistic functions; Figure S19C, Supporting Information) consistently outperformed controls and alternative models, demonstrating robustness against overfitting. Since the absolute probability values of SFER were similar across individual mice, cross‐animal predictions were feasible (Figure S19D, Supporting Information). Results from cross‐animal validation closely mirrored those from cross–spatial map validation (Figure S19E,F, Supporting Information). Collectively, these findings establish that SFER‐featured models outperform alternative models in predicting single‐field evolution.

### 2.9 SFER with Inter‐Field Interactions

**Figure 7:**
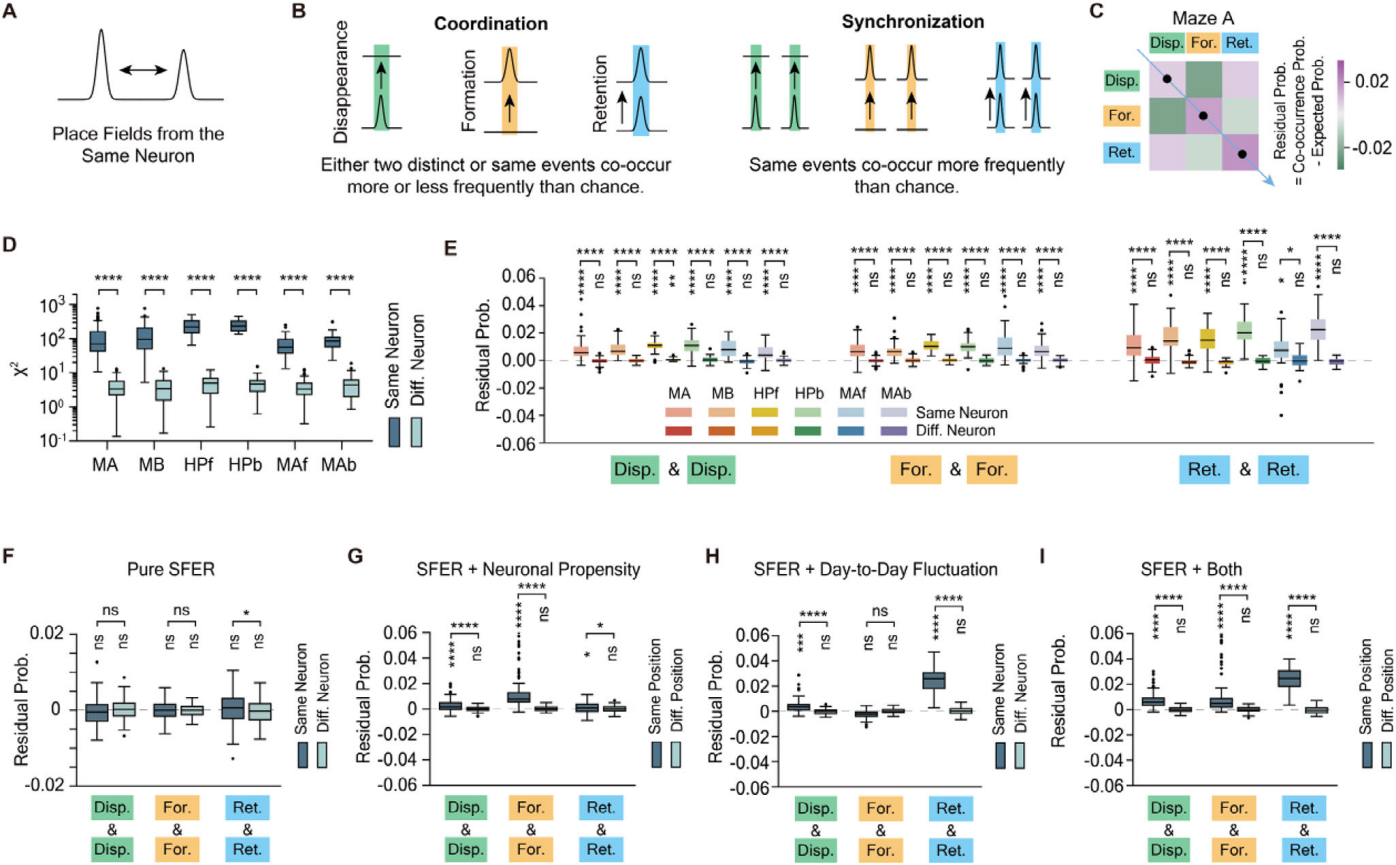
SFER with inter‐field interactions reproduces observed inter‐field synchronization. A) This panel examines the interrelationships in the evolution of place fields within the same neuron. B) Definitions of coordination and synchronization are provided. C) An example residual probability matrix illustrates the degree to which pairwise co‐occurrence probabilities of two evolutionary events exceed expectations. D) The χ^2^ statistic indicates significant coordination within the place fields of the same neuron. E) The co‐occurrence of the same evolutionary events is more frequent than expected. F) Pure SFER fails to reproduce inter‐field coordination. G) SFER integrating neuronal propensity successfully replicates the synchronization of field‐retention events. H) SFER that incorporates day‐to‐day fluctuations effectively reproduces the synchronization of field disappearance and retention events. I) SFER with both corrections replicates the observed inter‐field coordination. Outliers are defined as 1.5 IQR. One‐sided one‐sample t‐test is used to assess if values are greater than 0 (significance levels indicated by stars below the bracket), and two‐sided paired t‐test is used to compare the levels observed within place fields from different neurons. Data from 6 mice, Significance levels: ns: *P* ≥ 0.05; ^*^: *P* < 0.05; ^**^: *P* < 0.01; ^***^ *P* < 0.001; ^****^ *P* < 0.0001.

To this point, we have explored multiple factors that typically influence single‐field stability. However, while the function of place fields from different neurons has long been assumed to be highly independent,^[^ ^66^, ^67^ ^]^ cellular properties^[^ ^51^ ^]^ have also been proposed as critical factors that can affect place fields of the same neuron (sibling fields) simultaneously (**Figure** **7**A). This interdependency among sibling fields warrants careful consideration.

To address this question, we proposed the SFER with inter‐field interactions. Based on the three evolutionary events: the disappearance, formation, and retention of individual fields, we broadly define “coordination” as any significant increment or decrement in the co‐occurrence probability of these events among sibling fields (Figure 7B). In contrast, “synchronization” specifically refers to significant increments in the co‐occurrence probability of the same events, indicating a tendency for two place fields to disappear, emerge, or maintain simultaneously (Figure 7B).

Using the χ^2^ statistic as the metric to assess deviations from chance‐level probabilities, we found that sibling fields exhibited significant coordination across all spatial maps (Figure 7C,D). Further analysis revealed that this coordination is characterized by synchronization, with the same evolutionary events occurring more frequently among sibling fields (i.e., the residual probability is significantly greater than 0) (Figure 7C,E), a pattern not observed in place fields from different neurons. To minimize the influence of slow calcium transient dynamics on sibling field identification, we included only well‐separated sibling fields with inter‐field intervals greater than 50 cm. While SFER primarily focuses on single fields, it faces challenges in accounting for the observed coordination (Figure 7F), necessitating appropriate corrections. We first introduced a mechanism to assign individual place fields to neurons. According to the Gamma‐Poisson model, the dCA1 neural population exhibits heterogeneity in its capacity to form place fields^[^ ^56^ ^]^, which increases the likelihood of simultaneous formation of multiple fields. Indeed, we observed significant synchronization in field‐formation events when incorporating neuronal propensity into SFER predictions (Figure 7G; see Experimental Section). Additionally, day‐to‐day fluctuations can impact the stability of active sibling fields, leading to strong synchronization in disappearance and retention events (Figure 7H). A combination of the field assignment mechanism and day‐to‐day fluctuations has been able to explain, at least in part, the observed synchronization (Figure 7I).

## 3 Conclusion and Discussion

By recording dCA1 pyramidal neurons within complex and hairpin mazes (Figure 1; Figure S1, Supporting Information), we established a dataset rich in place cells exhibiting multiple fields (Figure 1; Figures S3–S7, Supporting Information). Binary representation of place fields’ evolutionary trajectories provides a crucial simplification (Figure 2), allowing us to capture the history dependency of single‐field evolution, characterized by history‐dependent retention and recovery probabilities (Figure 3). This dependency is unlikely to be a technical artifact of one‐photon imaging, not only because of our validations on imaging and tracking (Figures S7–S11, Supporting Information) but also because that a contemporaneous two‐photon study reported similar results.^[^^]^ This dependency is context‐irrelevant (Figure 3), decision‐making‐irrelevant (Figure 3), position‐irrelevant (Figure S13, Supporting Information), and, most importantly, novelty‐irrelevant (Figure S14, Supporting Information). These properties allow us to pool state sequences of place fields formed in different sessions together and suggest that history dependence may be an intrinsic property of dCA1 place fields.

Based on these findings, we proposed a general paradigm for modeling single‐field evolutionary dynamics: decoding the re‐expression probability *P_t_* given a field‐state sequence (Figure 4A). This approach reframes our question as one of sequential learning, leading to the discovery of the single‐field evolution rule in the general sense, or, for short, SFER: active and inactive states serve as opposing factors in determining *P_t_* (Figure 4B–G). SFER consists of two increasing functions—*f* in Equation (1) for the incremental update of *P_t_* and *g* for the decremental update—empirically selected from elementary functions like polynomials (Figure 5; Figures S16 and S17, Supporting Information). Notably, SFER defines a first‐order temporal pattern, establishing a general relationship between *P_t_* and preceding probability *P*_*t* − 1_. While GRU, HMM, and SFER models indirectly incorporate earlier sequential information through recursive updates, higher‐order temporal patterns remain unexplored here.

An important implication of SFER for the long‐term dynamics of dCA1 spatial representation is that any stochastic discrete dynamical systems defined by SFER inherently exhibit a dual‐fate dynamic and a convergently decremental drift rate (Figure 5; Figure S16, Supporting Information). This outcome arises naturally from the properties of *f* and *g*, which collectively display two fixed points—the intersection points between *f* or *g* and the diagonal. An active field‐state sequence drives *P_t_* toward the high fixed point, while an inactive sequence drives it toward the low *P_t_* ​ fixed point. These two attractor‐like structures to which place fields tend to evolve, necessarily determine the dual‐fate dynamics (Figure 5C–E; Figure S17, Supporting Information). This property of SFER explains the rise of representational stability during familiarization,^[^ ^7^, ^11^, ^12^, ^31^, ^32^, ^33^, ^49^ ^]^ the presence of super‐stable fields^[^ ^3^, ^4^ ^]^ (Figure 2A; Figure S10, Supporting Information), and aligns with single‐field stabilization.^[^ ^16^ ^]^ Furthermore, it suggests that dCA1 representational drift dynamics differ from those in the piriform cortex^[^ ^40^ ^]^ (Figure 6, Model II to V vs Model VI) and are unlikely to be driven solely by learning under noisy synaptic inputs.^[^ ^46^ ^]^ Recent theoretical works proposed that dCA1 representational drift results from implicit regularization,^[^ ^68^ ^]^ exhibiting similar convergent dynamics as SFER. Whereas other computational frameworks interpret drift dynamics at the network level and simulate them using artificial networks, SFER offers a biologically plausible implementation—activity‐dependent plasticity that bidirectionally influences the re‐expression probability (*P_t_*) of single fields—thereby generating population‐level drift dynamics.

Researchers have identified various factors beyond history dependency that may influence dCA1 representational stability, including the passage of time,^[^ ^7^, ^9^, ^10^ ^]^ active experience,^[^ ^29^ ^]^ synchronized activity,^[^ ^7^ ^]^ reward expectation,^[^ ^69^, ^70^, ^71^ ^]^ precision of field,^[^ ^72^ ^]^ attention and engagement,^[^ ^73^, ^74^ ^]^ exercises,^[^ ^75^, ^76^ ^]^ behavioral consistency,^[^ ^77^ ^]^ cellular excitability,^[^ ^52^, ^78^ ^]^ fos‐expression levels,^[^ ^51^ ^]^ trajectory stability,^[^ ^77^ ^]^ and branch spike prevalence.^[^ ^20^ ^]^ Some factors previously thought to contribute to drift, such as mild day‐to‐day changes in environmental features, have recently been shown to exert no detectable influence on drift rate.^[^ ^52^ ^]^ Studies in complex mazes reveal a significant number of place fields with lifespans exceeding 35 days (Figure 2B), a rarity in 1.5‐m linear tracks.^[^ ^7^ ^]^ This may result from the increased attention demands^[^ ^73^ ^]^ during complex navigation or the asymmetric, cue‐rich environment. A series of generalized linear models that considered multiple factors beyond SFER (Figure 6). Among these, peak rate (and peak calcium transients) and within‐session stability emerged as the most influential components (Figure 6E), consistent with the fact that neurons with high branch spike prevalence^[^ ^20^ ^]^ or cellular excitability^[^ ^52^ ^]^ display higher stability and within‐session reliability. Notably, the field state itself (Figure 6C, Model I) accounted for much of the single‐field evolution, reflecting the distinct average levels of *P_t_* for active versus inactive fields. Furthermore, SFER models (Model IVs), when fitted with four‐parameter linear functions (abbreviated as SFER‐linear), achieved performance comparable to HMMs and GRU models, despite the latter requiring many more parameters (Figure 6E, Model III to V). Cross‐spatial‐map and cross‐animal validations further demonstrated that models such as SFER‐quadratic, SFER‐cubic, and HMMs are prone to overfitting, whereas SFER‐linear and GLM models remain robust, suggesting that they capture temporal features consistently preserved across independent recordings and individual animals (Figure S19C,F, Supporting Information). Moreover, element‐based models improved significantly when integrating *P_t_* predicted by SFER‐linear, compared with incorporating field states alone (Figure 6E, Model VIII vs IX; Figures S18B,C and S19C,F, Supporting Information), underscoring SFER's effectiveness in predicting single‐field evolution.

Pure SFER does not inherently account for inter‐field relationships among sibling fields (Figure 7F), which restricts its applicability to some extent. Our analysis reveals that these sibling fields tend to evolve in a synchronized manner (Figure 7A–E), likely due to non‐specific cell‐to‐field influences. Two notable challenges arise: 1) Accurately assessing the degree of inter‐field coordination is impeded by limited *z*‐axis resolution and potential signal crosstalk from adjacent regions of interest (ROIs) in one‐photon recordings. This limitation may dilute observed coordination by mixing nearby independent neural signals. Nevertheless, our SFER correction methodology is grounded in the principles of coordination (i.e., synchronization), rather than the degree of coordination, exhibiting robustness across varying coordination intensities. 2) While the Gamma‐Poisson model effectively accounts for place field formation during the first session,^[^ ^8^, ^56^ ^]^ extending its applicability to later sessions remains challenging. Our approach might have offered a feasible alignment of single‐field evolution with the Gamma‐Poisson model, successfully replicating observed coordination in field‐formation events (Figure 7G). Although additional, unaccounted factors may contribute to inter‐field coordination, SFER with inter‐field interactions emerges as a novel framework, striking a balance among model parsimony, interpretability, and efficiency, while remaining a high extensibility.

The specific activity‐dependent synaptic plasticity underlying SFER warrants attention in future studies. When animals enter a novel environment, dCA1 rapidly constructs an initial map of that environment^[^ ^32^ ^]^ through the amplification or transient disinhibition of membrane potentials,^[^ ^16^, ^20^ ^]^ with this process occurring more quickly than in CA3.^[^ ^24^, ^79^ ^]^ Within this initial map, most place fields emerge during the very first lap,^[^ ^20^, ^24^ ^]^ suggesting a strategy that leverages pre‐strengthened synapses for rapid map formation, albeit at the cost of reduced spatial information content and stability.^[^ ^11^ ^]^ These early place fields may accept complex upstream input profiles, and some may transiently fall silent for several sessions due to multiple possible factors: 1) changes in presynaptic inputs from CA3, dentate gyrus, CA2, EC3. As one of the major inputs to dCA1, place codes in CA3^[^ ^5^, ^24^ ^]^ and dentate gyrus^[^ ^5^ ^]^ display greater cross‐day stability than those in dCA1, whereas place codes in CA2 are far more dynamic,^[^ ^80^ ^]^ likely contributing ongoing variability to dCA1 inputs. Moreover, newborn neurons generated through adult neurogenesis in the dentate gyrus,^[^ ^35^, ^36^, ^37^ ^]^ must be integrated into local networks, potentially inducing long‐term remodeling of downstream circuits. 2) Random day‐to‐day fluctuations in neuronal excitability, such that inputs with intensities near the threshold for activating a neuron may yield entirely different outputs despite stable upstream activity. 3) The nonlinearity of dendritic integration, which may prevent fluctuating upstream inputs from consistently generating sufficiently strong postsynaptic potentials to propagate to the soma. Collectively, multiple factors act unpredictably on each dCA1 neuron, introducing stochasticity in the activity of place fields. Accidental inactivity weakens the corresponding dendritic synapses via activity‐dependent synaptic plasticity,^[^ ^55^ ^]^ thereby reducing the probability of future re‐expression, as supported by observations of disappearing dendritic spines.^[^ ^34^ ^]^ Conversely, place fields driven by strong and temporally consistent inputs are more reliably re‐expressed, as activity‐dependent plasticity strengthens their dendritic synapses, likely through NMDA receptor–dependent pathways.^[^ ^54^, ^81^ ^]^ In this way, SFER and its biological implementations function as a selector: fields with unstable inputs or adverse factors against sustained activity are gradually eliminated, while those with stable inputs persist. Over time, the initial map—formed instantly for temporary and “urgent” use—evolves into a super‐stable representation. By implementing activity‐dependent plasticity rules at the single‐field level, population‐level dynamics inherently emerge (Figure 5).

Plasticity‐inducing mechanisms during sleep, particularly those involved in memory consolidation,^[^ ^82^, ^83^, ^84^, ^85^, ^86^ ^]^ may also contribute to the implementation of SFER. Large‐scale neural network reorganization during rest is widely recognized as critical for consolidation, with population activity patterns re‐emerging during sleep.^[^ ^86^ ^]^ Notably, such reactivation has recently been shown to predict future sensory responses in cortical regions.^[^ ^87^ ^]^ Additional evidence suggests that rewards generally enhance representational stability,^[^ ^69^, ^70^ ^]^ potentially via a “tagging” mechanism that prioritizes rewarded experiences for consolidation.^[^ ^88^ ^]^ Together with the coexistence of both destabilization and stabilization processes during reconsolidation,^[^ ^89^, ^90^, ^91^ ^]^ these findings highlight the need to clarify how rest‐phase activity contributes to single‐field evolution. Moreover, mechanisms intrinsic to place‐field expression may also play a role. For example, fields with strong peak rates and calcium transients may arise from rare BTSP events.^[^ ^17^, ^92^ ^]^ Ultimately, multiple plasticity rules—both established and yet to be discovered—likely converge to support the activity‐dependent plasticity underlying SFER, warranting further comprehensive investigation.

Species differences may contribute to diverging representational dynamics. It is important to note that most early studies of hippocampal representational stability were conducted in rats using unitary recordings,^[^ ^58^, ^59^ ^]^ whereas our work—consistent with other population‐level recordings—focuses on mice.^[^ ^6^, ^9^, ^29^ ^]^ Notably, mice and rats differ in dCA1 coding properties, particularly in their propensity to form multi‐field representations. In rats, multiple place fields typically emerge only in large mazes,^[^ ^59^, ^61^ ^]^ whereas CA1 place cells in mice often display multiple fields even in standard enclosures.^[^ ^63^ ^]^ Although a few studies have recently applied two‐photon large‐scale calcium imaging in rats,^[^ ^93^ ^]^ revealing error‐driven drift^[^ ^94^ ^]^ in their hippocampal representations, it remains unclear whether their long‐term dynamics during repeated exposure to the same environment are governed by SFER in a manner comparable to mice. Future cross‐species investigations will therefore be critical to establish the generality of SFER.

## 4 Experimental Section

### Animals

Mice (C57BL/6N, male and female) were group‐housed in a controlled environment under a reverse 12‐h light/dark cycle. Prior to undergoing surgery, they had ad libitum access to standard laboratory chow, water, and a running wheel for enrichment. Surgeries for implanting the necessary devices were conducted at ≈10–14 weeks of age. Post‐surgery, mice were singly housed to prevent implant damage and facilitate recovery. GCaMP6f was expressed in the hippocampal dCA1 region of six mice (IDs: #10209, #10212 (female), #10224, #10227, #10232, and #10234), which were utilized for all behavioral paradigms and imaging studies. Two additional mice (IDs: #11092, #11095) expressing GCaMP6s were trained exclusively for the maze navigation paradigm; their imaging data were used solely to validate the Poisson distribution of field numbers. All procedures were approved by the Institutional Animal Care and Use Committee at Peking University and adhered to ethical guidelines for animal research (ethical approval number: LSC‐MiaoCL‐1).

### Surgery

Virus Injection: Animals were initially anesthetized with 5% isoflurane in air in a chamber and subsequently placed in a stereotactic apparatus (Kopf Instruments) with 1‐2% isoflurane. The fur over the skull was removed, and the head window was carefully cleared. Using a stereotactic drill, holes were made at the designated injection sites (AP: −1.82 mm, ML: −1.25 mm; AP: −2.5 mm, ML: −2.28 mm). rAAV‐hsyn‐GCaMP6f‐SPRE‐hGH‐pA (titer: 1 × 10^12^) was injected via a beveled steel needle, positioned 1.4 mm below the dura, at a rate of 100 nL min^−1^, delivering 200–250 nL at each site. The incision was then sutured, and the mice were allowed to recover for 2–3 weeks to ensure optimal viral expression.

Lens Implant: With similar anesthetic procedures, the skull was re‐exposed, and a craniotomy of ≈2.0 mm in diameter was performed with a hand‐held drill, centered over the previous virus injection sites. The skull was removed, and the cortex was gently aspirated until the stripy corpus callosum became transparent. Three set screws were affixed to the skull for added stability and attachment of the cement. The GrinTech lens was slowly lowered into the optimal viewing position determined under the monitor of the UCLA miniscope connecting to the DAQ box, and the lens was secured in place with Metabond dental cement. A tube cap was placed over the lens for protection. Postoperatively, ceftriaxone sodium (1.25 mg kg^−1^) was administered intraperitoneally to the animals for 3 to 7 days. The animals were then returned to their home cages for a recovery period of 1–2 weeks.

Baseplate: The miniscope was connected to the DAQ software and affixed to the aluminum baseplate at the bottom of the miniscope. The miniscope was then mounted above the lens, and its position and focal length were adjusted to obtain the optimal field of view. The miniscope was secured to the holder, allowing the baseplate to be cemented just above the optimal viewing position. After the cement had dried, the miniscope was detached, leaving the baseplate attached to the animal's head for subsequent reattachment and imaging sessions. Finally, a Lego block was attached as a protective cap for the lens.

### Histology

Mice were sedated intraperitoneally with avertin. Subsequently, they were perfused transcardially with 0.9% saline, followed by 4% paraformaldehyde (PFA). Brains were extracted and postfixed in 4% PFA at 4 °C for 24 h. Afterward, the fixed brains were transferred to 30% sucrose for dehydration. The tissues were rapidly frozen using instant freeze spray and sectioned into 40 µm slices. Brain sections were mounted directly onto slides and washed with PBS three times for 5 min each at room temperature. Sections were blocked for at least 1 h in PBS containing 10% goat serum and 0.1% Triton X‐100. They were then incubated overnight at 4 °C with the primary antibody Chicken‐anti‐GFP IgY (1:1000, Invitrogen, A10262) to label GCaMP6f‐positive neurons. After primary antibody incubation, sections were rinsed three times in PBS and incubated for 2 h with the secondary antibodies Alexa Fluor 488 Goat‐anti‐Chicken IgG (1:1000, Invitrogen, A11039) and DAPI (1:1000, Sigma, D9542) for nuclear staining.

### Behavioral Training

Mice were singly housed with a running wheel and Lego in their home cage post‐surgery, with free access to food and water until three days before training. During training, food was restricted to maintain their weight at ≈80% of their baseline to ensure motivation.

### Open Field

The open field arena was squared (1 m × 1 m × 0.5 m, length × width × height). The environment was marked by distinct black shapes and stripes on four white walls, illuminated by dim white light, with three sides surrounded by black curtains. Recording began when the mice started actively exploring the environment. Crumbs of butter cookies, sometimes mixed with chocolate, were randomly distributed in the open field used as incentives. Sessions lasted ≈30 min or until the mice covered the entire area. Mice were subjected to a pre‐training phase within the open field for 20∼27 30‐min sessions over 3 weeks. During Stage 1 and Stage 2, mice were exposed to this open field for two 30‐min sessions before and after the maze session(s), respectively.

### Maze Navigation Paradigm (MNP)

Both mazes were squared (0.96 m × 0.96 m × 0.5 m, length × width × height). Maze A was constructed as a 12 × 12 lane grid with 8 cm wide lanes, featuring a total path length of 8.88 m and 17 decision points. The walls were adorned with distinct shapes and stripes to serve as distal cues. Maze B, while identical in size and the number of decision points, had a shorter correct path measuring 8.08 m. The environmental setup, including visual cues and lighting, remained consistent for both mazes.

Training was divided into two stages, each comprising 13 blocks spread over 26–29 days. In Stage 1, a block consisted of three successive sessions: open field, Maze A, and open field again. This sequence aimed to familiarize mice with Maze A while reinforcing their acclimation to the open field. In Stage 2, a Maze B session was added after Maze A.

A “lap” in maze navigation was defined as the interval between a mouse entering and exiting the maze. Mice were required to complete at least 5 laps in the first session of both mazes and at least 10 laps in subsequent sessions. Each maze session lasted ≈30 min, except for the initial maze sessions, which extended over an hour to accommodate the time‐consuming process of locating the exit for the first time. Food rewards were present at the exit.

### Reversed‐Maze Paradigm (RMP)

The environmental setup was consistent with previous settings. Mice previously familiarized with forward navigation in Maze A were trained to traverse back and forth between the entry and exit. A lap, whether forward or backward, was defined similarly. Each 30‐min session comprised 21–48 laps in total, with 10–24 laps in each direction. Training lasted for 7 to 12 sessions over 7 to 15 days, with 2 mice completing the shorter duration and 2 mice completing the longer duration.

### Hairpin‐Maze Paradigm (HMP)

A linear navigation task was implemented in a hairpin maze, designed to exclude decision‐making while retaining the characteristics of the complex mazes. This included identical dimensions, material, and wall height, but with a different arrangement of the internal barriers. The total path length within the maze was 11.52 meters. Environmental cues and the setup of the room mirrored those used in the maze training sessions. Rewards were strategically placed at both ends of the track. Each 30‐min training session comprised 15–48 laps, spanning 7 sessions conducted over a week.

The environment setups were cleaned carefully by 75% ethanol every time after ending a recording session.

### Data Analysis‐Behavioral Data

Animal behavior was captured using a top‐view webcam at a frame rate of 20 Hz. The onset and end of each navigational lap were manually labeled. DeepLabCut^[^ ^95^, ^96^ ^]^ was employed to track the mice's positions, with the neck point being annotated to represent the animal's position. Preprocessing behavioral data involves: 1) Removal of all NaN and erroneous values, with errors defined as points with a transient speed exceeding 100 cm s^−1^. 2) Discarding inter‐lap data. 3) Rectification of visual perspectives caused by film angles through an affine transformation. 4) Binning of the transformed trajectory into 2 cm bins for each dimension. 5) Application of a cross‐wall correction to adjust or remove points that cross walls. All mazes have a unique path connecting the entry and exit, referred to as the *correct track*. Neural activity along the correct track could then be visualized based on the fields’ or calcium events’ distances toward the entry.

### Behavioral Indexes

To evaluate behavioral performance, two basic indexes were defined for each lap: 1) the duration spent by the mouse and 2) the mouse's mean speed during the lap. For each session, the correct decision rate was defined as the ratio of correct decisions at decision points to the total number of decision points encountered while moving forward. The behavioral learning progress in each session was determined by dividing the current progress by the total progress. The total progress was calculated by taking the absolute value of the difference between the maximum and minimum values of the animal's behavioral index.

### One‐Photon Calcium Imaging and Signal Preprocessing

The UCLA miniaturized micro‐endoscope (Miniscope V3)^[^ ^97^ ^]^ was custom‐built following a previous design. Calcium imaging data collected by the miniscope were synchronized with behavioral data from a top‐view webcam at 20 Hz using Miniscope DAQ software. Downsampled AVI videos underwent motion correction with NoRMCore.^[^ ^98^ ^]^ CNMF‐E^[^ ^99^ ^]^ was employed to delineate regions of interest (ROIs), extract the dF/F signals of each neuron, and deconvolve calcium signals. Ssub: 1; tsub: 5; gSize: 15 pixels; gSig: 3 pixels; ring_radius of bg_model: 18; smin: ‐5; min_pnr: 8; Data from all sessions were included in the analysis, with the exception of Stage 1, Session 6 in Maze A for mouse #10212 due to the mouse's physical weakness and evident disengagement from the navigation task.

### Signal Processing

Deconvolved signals were binarized, with the deconvolved values greater than three times of the standard deviation being considered as calcium events. Frames corresponding to speeds below 2.5 cm s^−1^ were discarded. Inter‐lap neural activities were excluded, and behavior of wrong directional movements, as well as activity on incorrect tracks, were excluded.

### Signal‐to‐Noise Ratio

Defined as

(2) $$\frac{\max\frac{\Delta F}{F}}{\sigma \left(\frac{\Delta F}{F}\right)}$$

where the $\sigma (\frac{\Delta F}{F})$ is the standard deviation of the lowest 75% of calcium raw traces values, which reflects the level of the baseline.

### Calculation of Spatial Rate Maps

Calcium event rate maps were computed to visualize neural activity. The mazes were divided into 48 × 48 square spatial bins (numbered 1 to 2304, 2 cm × 2 cm). The occupation time spent by mice in each bin was calculated, and the number of events in each bin was counted. Raw event rate was simply a ratio between these two. Events rate in bins with occupation time lower than 50 ms were set as NAN. A Gaussian kernel considered the maze walls (σ = 2) was applied to smooth event rate.

### Spatial Information

The calculation of spatial information (SI) follows the methodology established in previous research.^[^ ^100^ ^]^ Briefly, SI is computed using the formula:

(3) $$SI=\frac{1}{\lambda }\sum_{i}\lambda_{i}\log_{2}\left(\frac{\lambda_{i}}{\lambda }\right)P_{i}$$

where λ is the mean firing rate of a neuron across all bins, λ_*i*_ is the firing rate of the neuron in bin *i*, and *P_i_* represents the fraction of time mice spent in bin *i*.

### Identification of Place Cells

Neurons were classified as place cells when passing all three shuffle tests: 1) Temporal Shuffle Test, which dissociates calcium events' timestamps from the mouse's positions. 2) Inter‐Event Interval Shuffle Test, which rearranges calcium events by shuffling their inter‐event intervals.^[^ ^83^ ^]^ 3) Random Shuffle Test: which randomly rearranges the calcium events. Each test was repeated 1000 times, and a neuron passed if its real SI exceeded 95% of the shuffle test SI values. Stability criteria were not applied to avoid limiting the exploration of dynamic neural representations, yet neurons meeting the criteria showed sufficient stability at the population level within sessions (Figure S2C–E, Supporting Information).

### Within‐Session Stability

To assess neuronal within‐session stability, the Pearson correlation of smoothed event rate maps between the session's first and second half of all laps was computed. Within‐session within‐field stability was computed similarly but only included event rates in the bins within a place field. For fields smaller than 16 bins, surrounding bins were included to ensure a minimum of 16 bins.

### Identification of Place Field

A place field was defined with multiple requirements. 1) Spatial bins with a smoothed event rate above 0.2 Hz were considered as candidates for place fields. 2) A minimum of 5 calcium events were required to be detected within fields. 3) Calcium events must be detected in at least five laps within a maze or during five separate visits in an open field, with a minimum interval of 15 s between each visit. 4) Closely spaced place fields were split into multiple fields if the saddle‐to‐peak ratio was lower than 0.5 (split threshold) (Figure S11A, Supporting Information).

### Naïve Bayesian Classifier

The position of animals was decoded by a modified Bayesian classifier. It uses a distance‐based loss rather than the 0‐1 loss the traditional Naïve Bayesian decoder employs, leading to a decision rule:

(4) $$f\left(s\right)=\underset{\hat{y}\in \left\{1,\text{…},Y\right\}}{\mathrm{argmin}}{\left(Dp_{t}\right)}_{\hat{y}}$$

where $\hat{y}$ is the predicted bin ID, *Y* is the total number of bins, $D\in R^{Y\times Y}$ is a matrix containing the distances between each two bins, and $p_{t}\in R^{Y}$ is a vector at frame *t*:

(5) $$D=\left[\begin{array}{ccc} Dis\left(1,\;1\right) & \text{…} & Dis\left(1,Y\right)\\ \vdots & \ddots & \vdots \\ Dis\left(Y,\;1\right) & \text{…} & Dis\left(Y,\;Y\right) \end{array}\right]$$

(6) $$p_{t}={\left[\prod_{i=1}^{N}\left[P\left(s_{i,t}|y=1\right)\right]*P\left(y=1\right),\;\text{…},\prod_{i=1}^{N}\left[P\left(s_{i,t}|y=Y\right)\right]*P\left(y=Y\right)\;\right]}^{T}$$

where $s_{i,t}\in \left\{0,\;1\right\}$ is whether a neuron *i* emitted a calcium event at a time frame *t*. Therefore, the modified classifier decodes the animal's position for each recording frame. The median absolute error (unit: cm) was used to assess the decoding results for each lap. Data from 60% laps were selected as the training set. The decoding efficiency was cross‐validated by rolling laps one‐by‐one and decoding the rest.

### Cross‐Session Decoding

To assess functional consistency, A Naïve Bayes decoder was employed. For each pair of sessions (*s_i_* and *s_j_*), the registered neurons present in both sessions were randomly downsampled to a subset of 100 neurons, as Naïve Bayes decoding accuracy was sensitive to feature dimensionality (i.e., the number of neurons here^[^ ^66^ ^]^). Activities of these 100 neurons in the session *s_i_* were used for training, and their activities in session *s_j_* for testing. Because downsampling introduces stochasticity into decoding loss, the procedure was repeated 5 times, and the mean loss was taken. Given the large number of possible pairings (tens of thousands), decoding was performed only when the interval between *s_i_* and *s_j_* was fewer than 10 sessions.

### Examination of Statistical Structure

Field sizes, measured by the number of bins, were fitted with either a log‐normal distribution or a gamma distribution as reported in a prior study.^[^ ^57^ ^]^ Field numbers were fitted by a negative binomial distribution.^[^ ^8^, ^56^ ^]^ Lilliefors‐corrected^[^ ^101^ ^]^ Kolmogorov–Smirnov tests were used, together with a bootstrap down‐sampling of the field number to 1621, a level reported in the prior study.^[^ ^57^ ^]^ Field numbers were down‐sampled to 500.

### Cell Registration

Neurons were tracked across days using the CellReg algorithm^[^ ^64^ ^]^ and corrected linear shifts in the field of view (FOV) with MultiStackReg.^[^ ^102^ ^]^ After initial correction, CellReg registered neuron indexes with a P_same threshold of 0.5, micron per pixel set to 2.3, and a maximum distance of 14 microns. To optimize registration, a re‐match algorithm was implemented: 1) CellReg was run with various spatial footprints as references, and 2) results from different references were compared. Optimization was applied only to spatial maps from Maze A and B; MAf, MAb, HPf, and HPb were not optimized. These findings demonstrate consistent results across all data, affirming that optimizing CellReg outputs does not compromise the conclusions. For mice #10209 and #10212, neurons identified during Maze‐A sessions were separately tracking in either Stage 1 or 2, resulting in 2 13‐session tracking for each mouse. For mice #10224 and #10227, neurons in both Stage 1 and 2 were tracked together, resulting in a 26‐session tracking for each mouse).

### Re‐Matching Strategy

CellReg aligns spatial footprints (SFPs) across sessions by applying rotation and translation relative to a chosen reference SFP. Because different reference SFPs can yield different alignment results, a re‐match optimization algorithm was developed to refine CellReg's outputs without introducing new neurons.

Formally, CellReg produces an index map $N\in R^{S\times Q}$, where *S* is the number of sessions and *Q* is the number of registered neurons. For a reference SFP k∈{1,…,S}, the index map is denoted as *N* ^(*k*)^. Each column of *N* ^(*k*)^, $N_{:,j}^{\left(k\right)}$, represents a registered neuron *j*, with entries corresponding to its session‐specific indices (0 if undetected). By selecting *S*′ sessions as references, a set of index maps were obtained:

(7) $$N=\left\{N^{\left(k\right)}:k\in R\right\},\;\;R\in \left\{k_{1},\;k_{2},\text{…},k_{S^{\prime }}\right\},\;\;S^{\prime }\leq S$$

Practically, 5 reference sessions were used for 13‐session stages (1, 3, 7, 10, 13) and 9 for 26‐session stages (1, 3, 7, 10, 13, 17, 20, 23, 26). One map, $N^{\left(k_{opt}\right)}$, was chosen as the main result; others served as supplementary candidates.

For each neuron *j* in $N^{\left(k_{opt}\right)}$, there exists at least one corresponding registered neuron *j* ^(*k*)^ in any other index map *N* ^(*k*)^, such that $N_{:,\;j}^{\left(k_{opt}\right)}$ and $N_{:,\;j^{\left(k\right)}}^{\left(k\right)}$ most entries at the same session positions. Based on this property, a candidate matrix $C\in R^{S\times S^{\prime }}$ was constructed by combining corresponding neurons across supplementary maps:

(8) $$C=\left[N_{:,\;j}^{\left(k_{opt}\right)},\text{…},N_{:,\;j^{\left(k\right)}}^{\left(k\right)},\text{…}\right],\;k\in R,\;k\neq k_{opt}$$

The goal is to identify the optimal registered neuron, $J_{best}={\left[\begin{array}{ccc} I_{1} & \text{…} & I_{S} \end{array}\right]}^{T}$, where each entry $I_{i}\in \left\{N_{i,\;j^{\left(k\right)}}^{\left(k\right)}:k\in R\right\}$ and *T* is the transpose.

The candidate set *S_J_* was defined as all finite combinations of these entries:

(9)

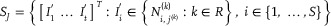




For any $J={\left[\begin{array}{ccc} I_{1}^{\prime } & \text{…} & I_{S}^{\prime } \end{array}\right]}^{T}\in S_{J}$, a uniquely determined likelihood matrix $P_{J}\in R^{S\times S}$ was defined, which specifies the pairwise likelihood that the selected indices correspond to the same neuron:

(10)

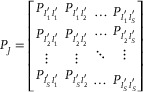

where $P_{I_{i}^{\prime }I_{j}^{\prime }}$ denotes the likelihood that neurons indexed by $I_{i}^{\prime }$ and $I_{j}^{\prime }$ are the same. A registered score, *RS*(*J*; α), was then defined, adapted from Sheintuch et al. (2017) with an additional structural term that favors registering more neurons together:

(11)

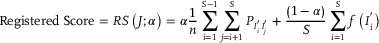

where α∈[0,1] (set to 0.5 in the analysis), *n* is the number of non‐NaN entries in the upper triangle of*P_J_*, and *f*(*x*) indicator function that equals 1 if $I_{i}^{\prime }>0$ is larger than 0 (indicating the neuron was detected in session *i*) and 0 otherwise. This modified registered score balances two objectives: 1) maximizing the likelihood that the registered indices belong to the same neuron, and 2) maximizing the number of sessions in which the neuron was detected, thereby ensuring sufficient samples for computing robust probabilities of place‐field fate.

Because the total number of candidate combinations was prohibitively large, a greedy optimization strategy was employed. The initial registered neuron was set as *J* ^(0)^ = *J_opt_*. At iteration *t*, each of the *S* entries of *J* ^(*t* − 1)^ was updated sequentially, while keeping the remaining entries fixed. For each entry $J_{i}^{\left(t-1\right)},i\in \left\{1,\text{…},S\right\}$, the update was performed by selecting the candidate that maximized the registered score:

(12)

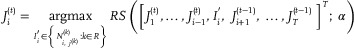




This iterative process continued until convergence (*J* ^(*t* + 1)^ = *J* ^(*t*)^) or *t* ≥ 20or until a maximum of 20 iterations was reached. Optimization was performed only for registered neurons with more than six non‐zero entries. The optimized *J_best_* is then returned for manual inspection, where decisions are made about whether to accept its proposed changes at each session position based on the neuron's spatial map across consecutive sessions.

All candidates were derived from CellReg's outputs, with no new neurons or raw candidates introduced. Optimization was applied only to Maze A and B maps, while RMP and HMP maps (MAf, MAb, HPf, HPb) relied directly on CellReg.

### Registration of Place Field Registration and Preparation of Field‐State Sequences

In RMP and HMP, place fields were considered bidirectional if they overlapped by 60% or more. The chance level was computed by shifting backward spatial maps to dissociate one‐to‐one relationships between forward and backward maps across place cells. To analyze individual place field evolution across multiple days, a multi‐step tracking approach was implemented: 1) Place fields were extracted from each session based on a rate threshold (see Identification of Place Field; Figure S11A, Supporting Information; Step 1). 2) Preliminary tracking was conducted using a 75% overlap threshold (Figure S11A, Supporting Information; Step 2). 3) Exception handling involved normalizing and summing event rates across all sessions. Fields with a saddle‐to‐peak rate ratio exceeding 0.5 were merged, while others remained distinct (Figure S11A, Supporting Information; Step 3). This process ensures clear separation of each tracked place field for precise analysis of evolution. Registered fields were then binarized, labeling active sessions as 1 and inactive sessions as 0 (Figure S11A, Supporting Information; Step 4). Unregistered neurons were assigned NAN values to indicate uncertainty. To determine the chance level of field recovery, the size and number of place fields for each neuron were maintained while randomly relocating their positions. This approach allows us to determine a baseline level, indicating the probability that the registered fields occurred by chance.

Field‐state sequences were prepared as follows: 1) NAN handling involved setting NAN values to 1 if a place field was active before and after a NAN. Fields were split at NAN values to include only continuous segments with clear field states. 2) Field‐state sequences began from the field‐formation session and ended before the first NAN or at the final session. This produced field‐state sequences of varying lengths, exceeding the number of registered fields. Each sequence was represented as ${\left[s_{1},\;s_{2},\;\text{…},\;s_{t}\right]}^{T}$, where $s_{i}\in \left\{0,\;1\right\}$ denotes observable field states, and t is the number of sessions. Field lifespan, as shown in Figure 2B, was defined as the length of each continuously active subsequence; registered fields could be counted multiple times if highly dynamic.

### Continuously Tracked Neuron Pieces

CellReg saves its results as a “cellRegistration.mat”, which contains an object called “cell_to_index_map (*M*)”. Here, *M* is a *N* × *S* matrix, where *N* is the number of registered neurons and *S* is the total number of sessions in which neurons are tracked. For a registered neuron *m*, if it is not detected or successfully registered in session *s_i_*, then the value in row *m*, column *i* is 0; it corresponds to the index of that neuron in session *s_i_*.

By a *continuously tracked neuron piece*, it was refered to a vector in matrix *M* of the following format:

(13) $$\left[\underset{\mathrm{Total}\mathrm{Length}=S(\mathrm{Sessions})}{\underset{︸}{{\overset{︷}{\text{…},0,0,}}^{>Two\;Zeroes}{\overset{︷}{{\overset{︷}{23,34,\;\text{…}\;,\;45\;,}}^{\mathrm{No}\mathrm{Zero}\mathrm{Allowed}}}}^{A\mathrm{continuously}\mathrm{tracked}\mathrm{neuron}\mathrm{piece}}{\overset{︷}{0}}^{\mathrm{End}\mathrm{by}0}{\overset{︷}{\text{…}}}^{\mathrm{Anything}}}}\right]$$

### Calculation of Field‐Retention and Field‐Recovery Probability

The retained duration *t_a_* is defined as the number of sessions a field has been active before *t* in a field‐state sequence. The silent duration *t_i_* represents the number of sessions a previously active field has been inactive before step *t*. The retention and recovery probabilities were empirically fitted using reciprocal function and Kohlrausch‐Williams‐Watts function, respectively:

(14) $$P\left(s_{t}=1|t_{a}\right)=f_{ret.}\left(t_{a}\right)=1-\frac{L_{1}}{t_{a}+b_{1}}$$

(15) $$P\left(s_{t}=1|t_{i}\right)=f_{rec.}\left(t_{b}\right)=ae^{-{\left(\frac{t_{i}}{b}\right)}^{c}}$$

### Models‐Overview

It was assumed there are *M* binary field‐state sequences of varied lengths, and denote the field‐state sequence of field j∈{1,2,…,M} as:

(16) $${\left[s_{1,\;j},\;s_{2,j},\;\text{…},\;s_{l_{j},j}\right]}^{T}$$

where $s_{t,j}\in \left\{0,1\right\}$ represents its observable field states at time step *t* and *l_j_* represents the total length of field *j*, i.e., the longest period this field can be continuously tracked since its formation, regardless of whether it is active or inactive.

All models adopted essentially predict the probability $P_{j}\left(s_{t,j}=1|s_{t-1,j},\;s_{t-2,j},\text{…},s_{1,j}\right)$, or simply, *P*_*t*, *j*_, for field‐state sequence *j* (Figure 5A).

Since *s*_1,*j*_ = 1 by definition, *P*_2,*j*_ is a fixed value for any j∈{1,…,M}:

(17) $$P_{2,j}=\frac{1}{M}\sum_{j\in \left\{1,\;\text{…},\;M\right\}}s_{2,j}$$

The loss was computed as the negative log‐likelihood (NLL). For a given time step *t* in the field‐state sequence (*t* = 12 in Figure 6E, *t* = 6 in Figure S18B, Supporting Information, and *t* = 18 in Figure S18C, Supporting Information), the NLL loss is defined as:

(18) $$\mathrm{NLL}\;\mathrm{Loss}\;≔-\frac{1}{M^{\prime }}\sum_{j\in \left\{1,\text{…},M^{\prime }\right\}}\left(s_{t,j}\log P_{t,j}+\left(1-s_{t,j}\right)\log\left(1-P_{t,j}\right)\right)$$

where *M*′ is the number of field‐state sequences with lengths *l_j_* ≥ *t*. Unless explicitly stated otherwise, all models were trained to minimize the NLL loss. Training used 80% of the sequences, while losses were calculated over the remaining 20%. 10 times of cross‐validations were conducted, and the results were averaged for model.

Each model can predict *P_t_* for 2 ≤ *t* ≤ *T*, where *T* is the maximal length of the sequences. Simulated sequences were generated through the following steps: 1) Initialize *P*_2_ and set *s*_1_ = 1. 2) Generate *s_i_* based on *P_i_* for *i* ≥ 2. 3) Models predict *P*_*i* + 1_. 4) Repeat steps 2 and 3 until *i* = *T*.

I: Field State‐Dependent Equal‐Rate Drift Model

(19) $$P_{t}=\left\{\begin{array}{c} P_{a},\;\;if\;s_{t-1}=1\\ P_{i},\;\;if\;s_{t-1}=0 \end{array}\right.$$

where *P_a_* and *P_i_* are constants.

II: SFER in the preliminary sense

(20) $$P_{t}=\left\{\begin{array}{c} f_{ret.}\left(t_{a}\right),\;\;if\;s_{t-1}=1\\ f_{rec.}\left(t_{i}\right),\;\;if\;s_{t-1}=0 \end{array}\right.$$

Fitting was achieved using the curve_fit function from SciPy.optimize.

III: Hidden Markov Model

The Hidden Markov Model (HMM) posits that an observable sequence was generated by a hidden‐state sequence. Here, the observable state at step *t* is the place field state $s_{t}\in \left\{0,\;1\right\}$, while the hidden state $h_{t}\in \left\{h_{1},\;\text{…},\;h_{N}\right\}$ is predetermined, with *N* varying among 5, 10, 20, and 40. The HMM has three basic parameters: the transition probability matrix $A\in R^{N\times N}$, the emission probability matrix $B\in R^{N\times 2}$, and an initial probability distribution over hidden states $\pi \in R^{N}$. In the modeling, *B* was determined by *N*:

(21) $$B=\left[\;1-{\left[\frac{1}{2N},\;\text{…},\frac{2N-1}{2N}\right]}^{T},{\left[\frac{1}{2N},\;\text{…},\frac{2N-1}{2N}\right]}^{T}\right]$$

For example, with 5 hidden states, the emission probabilities correspond to [0.1,0.3,0.5,0.7,0.9] probability of field re‐expression. The initial hidden state π is determined by the closest emission probability to *P*_2_, and all place fields were initially set in this hidden state.

(22) $$j=\underset{j\in \left\{1,\;2,\text{…},N\right\}}{\mathrm{argmin}}\left|B_{i,\;2}-P_{2}\right|$$

(23) $$\pi_{i}=\left\{\begin{array}{c} 1,\;\;if\;i=j\\ 0,\;\;if\;i\neq j \end{array}\right.$$

After field formation, it initializes with a hidden state *h_j_* at step 2, being either active or inactive. This process repeats for each session: the hidden state switches or maintains its state based on the transition probability, and emissions are determined accordingly. Thus, only the transition probability matrix was fitted to the data using the Baum‐Welch algorithm. The model‐predicted probability *P_t_* was computed through the forward algorithm. The HMM model was custom coded in PyTorch.

IV: SFER in the general sense

(24) $$P_{t}=s_{t-1}f\left(P_{t-1}\right)+\left(1-s_{t-1}\right)g\left(P_{t-1}\right)$$

where, if *s*_*t* − 1_ = 1, it simplifies to *P_t_* =  *f*(*P*_*t* − 1_) to increase *P*_*t* − 1_; Conversely, if *s*_*t* − 1_ = 0, it becomes *P_t_* =  *g*(*P*_*t* − 1_) to decrease *P_t_*. The updated *P_t_* is constrained within the interval [0,  1]. Parameters of candidate functions were optimized using scipy.optimize.minimize based on negative log‐likelihood (NLL) loss. The candidate functions include:

1. Linear function (Figure S16A, Supporting Information):
(25) $$P_{t}=s_{t-1}\left(a_{1}P_{t-1}+b_{1}\right)+\left(1-s_{t-1}\right)\left(a_{2}\left(1-P_{t-1}\right)+b_{2}\right)$$
2. Logistic function (Figure S16B, Supporting Information):
(26) $$\begin{array}{ccc} P_{t} & = & s_{t-1}\left(\frac{1}{1+e^{-a_{1}\left(P_{t-1}-b_{1}\right)}}\right)\\ & & \;+\left(1-s_{t-1}\right)\left(1-\frac{1}{1+e^{-a_{2}\left(1-P_{t-1}-b_{2}\right)}}\right) \end{array}$$
3. Quadratic function (Figure S16C, Supporting Information):
(27) $$\begin{array}{ccc} P_{t} & = & s_{t-1}\left(a_{1}P_{t-1}^{2}+b_{1}P_{t-1}+c_{1}\right)\\ & & \;+\left(1-s_{t-1}\right)\left(a_{2}{\left(1-P_{t-1}\right)}^{2}+b_{2}\left(1-P_{t-1}\right)+c_{2}\right) \end{array}$$
4. Cubic function (Figure S16D, Supporting Information):
(28) $$\begin{array}{ccc} P_{t} & = & s_{t-1}\left(a_{1}P_{t-1}^{3}+b_{1}P_{t-1}^{2}+c_{1}P_{t-1}+d_{1}\right)+\left(1-s_{t-1}\right)\\ & & \times \left(a_{2}{\left(1-P_{t-1}\right)}^{3}+b_{2}{\left(1-P_{t-1}\right)}^{2}+c_{2}\left(1-P_{t-1}\right)+d_{2}\right) \end{array}$$

The fixed points of *f* and *g* are defined as the points where *P_t_* = *f*(*P*_*t* − 1_) or *P_t_* = *g*(*P*_*t* − 1_). These fixed points are stable in the context of SFER properties and are thus able to accumulate place fields with *P_t_* close to the fixed points to form attractor‐like structures.

Numeric simulations were performed as follows: SFER models defined by quadratic functions were trained by real data. Then they were used to generate 10000 simulated sequences over 200 simulated sessions. For each step, a gaussian noise ε∼Normal(μ=0,σ=0.01)

V: Gated Neural Network

The recurrent neural network was implemented using a Gated Recurrent Unit (GRU) architecture. At each time step, the model takes two input features: the previous state *s_t_* (for *t* ≥ 2) and the probability *P_t_*. The model architecture is defined as follows:

1. A GRU layer:
(29) $$h_{t}=\mathrm{GRU}\left(x_{t},\;h_{t-1}\right)$$

1. A linear layer followed by a sigmoid activation function:
(30) $$P_{t+1}=\sigma \left(Wh_{t}+b\right)$$

where *x_t_* = [*s_t_*, *P_t_*] and *h*_*t* − 1_ is the hidden state from the previous step. where σ is the sigmoid function, *W* is the weight matrix, and *b* is the bias. Negative log‐likelihood (NLL) loss was utilized for training, and early stopping was implemented to balance loss minimization and overfitting prevention. The hyperparameters were set as follows: hidden sizes of 8, 16, and 32; batch size of 2048; training epochs of 1000; early stopping epochs of 100; and a learning rate of 0.001. The code was customized using PyTorch.

VI: Equal‐Rate Model

(31) $$P_{t}=\mathrm{constant}$$

VII: GLM (All Elements)

The generalized linear model with a “binomial” family used in this analysis was essentially a form of logistic regression. Let $b=[b_{0},\;b_{1},\;\text{…},\;b_{9}]$ be the coefficients, and $x_{t-1}={\left[1,\;x_{1,\;t-1},\;\text{…},\;x_{9,t-1}\right]}^{T}$ denote the parameters at sequence length *t*. These parameters were standardized to have a mean of 0 and a standard deviation of 1. For place fields that were inactive during certain sessions, their within‐field stability, fluctuation of field centers, and first appearance lap were set, which were not available, to 0, 0, and the last lap, respectively. Other parameters, behavioral progression, the number of training sessions, intervals between sessions, time spent in the field, peak rate, and peak calcium transient can still be computed. *P_t_* is computed as follows:

(32) $$P_{t}=\frac{1}{1+e^{-bx_{t-1}}}$$

GLM fitting and prediction were performed using the statsmodels.api library.

VIII: GLM (All Elements) + Field State

This model builds upon the structure of Model VII, further expanding the input vectors with the additional field state at step *t* − 1: $b=[b_{0},\;b_{1},\;\text{…},\;b_{9},\;b_{10}]$, $x_{t-1}={\left[1,\;x_{1,\;t-1},\;\text{…},\;x_{9,t-1},\;s_{t-1}\right]}^{T}$.

IX: GLM + Field State + SFER

This model builds upon the structure of Model VIII, further expanding the input vectors with the additional *P*_*t* − 1_ at step *t* − 1: $b=[b_{0},\;b_{1},\;\text{…},\;b_{9},\;b_{10},b_{11}]$, $x_{t-1}={\left[1,\;x_{1,\;t-1},\;\text{…},\;x_{9,t-1},\;s_{t-1},\;P_{t-1}\right]}^{T}$. Here, *P*_*t* − 1_ is predicted using Model IV (SFER in the general sense), fitted by a cubic function.

X: GLM (One Element)

This model builds upon the structure of Model VII, but the input vectors are simplified to contain only single elements: $b=\left[b_{0},\;b_{1}\right],\;x_{1,\;t-1}={\left[1,\;x_{1,t-1}\right]}^{T},\text{…},x_{9,\;t-1}={\left[1,\;x_{9,t-1}\right]}^{T}$.

### Cross‐Spatial‐Map Validations

Training and testing sets consisted of field‐state sequences from different spatial maps, generating a map‐wise loss matrix for each mouse. Values from this matrix were averaged to yield a mean cross‐map loss per mouse (Figure S19A, Supporting Information)

### Cross‐Animal Validations

Testing sets were derived from Maze A (MA) data of four mice (#10224, 10227, 10232, 10234). Training sets could come from any spatial map as long as they were not from the same mouse. For each model, six average losses were obtained by training on MA, MB, MAf, MAb, HPf, or HPb and testing on MA.

### SFER with Inter‐field Interactions‐Examination of Inter‐Field Coordination

Disappearance (*e*_1_ = [1, 0]), formation (*e*_2_ = [0, 1]), and retention (*e*_3_ = [1, 1]) of place fields were three evolutionary events. For *N* place fields detected in consecutive sessions, including *N*_1_ disappearances, *N*_2_ formations, and *N* − *N*_1_ − *N*_2_ retention events, the marginal distributions of evolutionary events can be measured as:

(33) $$P_{1}=\frac{N_{1}}{N},\;P_{2}=\frac{N_{2}}{N},P_{3}=1-P_{1}-P_{2}$$

If two place fields were randomly selected, the probability for a co‐occurrence of event *e_i_* and *e_j_* is:

(34) $$P\left(e_{1},e_{2}\right)=P_{1}\times P_{2}$$

This joint probability was computed for sibling fields (place fields of the same neuron) and non‐sibling fields (fields from different neurons). If no inter‐field coordination exists, the joint probability *P*(*e_i_*,*e_j_*) = *P_i_P_j_* should hold for every i,j∈{1,2,3}. The χ^2^ statistic was used to assess how much the joint probabilities *P*(*e_i_*,*e_j_*) deviate from the expected values *P_i_P_j_* (Figure 7D):

(35) $$\chi^{2}=N\sum_{i=1}^{3}\sum_{j=1}^{3}\frac{{\left(P\left(e_{i},e_{j}\right)-P_{i}P_{j}\right)}^{2}}{P\left(e_{i},e_{j}\right)}$$

The residual matrix was also defined (Figure 7C):

(36) $$R=\left[\begin{array}{ccc} P\left(e_{1},e_{1}\right)-P_{1}P_{1} & \;P\left(e_{1},e_{2}\right)-P_{1}P_{2} & \;P\left(e_{1},e_{3}\right)-P_{1}P_{3}\\ P\left(e_{2},e_{1}\right)-P_{2}P_{1} & \;P\left(e_{2},e_{2}\right)-P_{2}P_{2} & \;P\left(e_{2},e_{3}\right)-P_{2}P_{3}\\ P\left(e_{3},e_{1}\right)-P_{3}P_{1} & \;P\left(e_{3},e_{2}\right)-P_{3}P_{2} & \;P\left(e_{3},e_{3}\right)-P_{3}P_{3} \end{array}\right]$$

Particular attention was given to the diagonal entries that represent the synchronization of identical evolutionary events (Figure 7E). Only sibling fields with intervals ≥ 50 cm were included.

### Gamma‐Poisson Model for Hippocampal Multi‐Field Coding

Initially proposed to explain the distribution of field numbers per neuron in multi‐field representations, the Gamma‐Poisson model illustrates that distinct neurons possess unique innate neuronal propensities, denoted as λ_*i*_ (unit: number of place fields per meter) for neuron *i*. The number of fields formed by neuron *i* in the initial session, denoted as *X_i_*, is a random variable governed by a Poisson distribution:

(37) $$X_{i}\;∼\;poisson\left(\lambda_{i}\right)$$

Rich et al.^[^ ^56^ ^]^ assumed that the neuronal propensity λ_*i*_ follows a gamma distribution:

(38) $$\lambda_{i}∼\;\mathrm{Gamma}\left(\alpha ,\;\beta \right)$$

Under this model, *X_i_* is governed by a mixture of Poisson and gamma distributions, resulting in a negative binomial distribution characterized by a shape parameter *r* and a scale parameter *p*:

(39) $$X\;∼\;\mathrm{NegBin}\left(r,\;p\right)$$

### Integration of Field Assignment Mechanism

Pure SFER cannot account for the presence of coordination primarily due to two reasons: 1) it lacks mechanisms to assign place fields to individual neurons, and 2) it does not incorporate mechanisms to determine the probability of forming new fields. While the Gamma‐Poisson model effectively models the formation of fields in the initial session, it faces challenge to explain field numbers in later sessions. This is because the assumption that the number of fields is a random variable, though reasonable for the first session, becomes inappropriate for subsequent sessions. The formation of place fields in later sessions must consider not only neuronal propensity but also the influence of existing fields formed in earlier sessions.

In the SFER with inter‐field interactions, the following steps were taken: 1) a total neuron number of *N* = 500 was determined, and 2) generated neuronal propensity λ_*i*_ for each neuron from a gamma distribution (α: 5.3, β: 9.4). Then 3) the initial field number for each neuron was generated from a Poisson distribution with rate λ_*i*_, resulting in a total field number of *M*_0_ (∼ 2500 fields). For a later session *t*, the number of active fields substantially decreases to *M_t_* < *M*_0_ due to drift following SFER. A mechanism was assumed to compensate for this drift and maintain the total number of active fields at a constant *M*_0_. Consequently, there are *M*_0_ − *M_t_* fields that need to be assigned to neurons at session *t*. It was assumed that neuron *i* has a satiety (*u_i_*) to form new place fields, determined by:

(40) $$u_{i,t}=\left(1-P\left(X_{i,t},\;\lambda_{i}L\right)\right)\times \lambda_{i}$$

where *P* is the cumulative distribution function of the Poisson distribution with rate λ_*i*_ and *L* is the track length (set at 9 m). This formula considers not only the existing place fields but also the neuronal propensity, representing the tendency of a neuron to form new place fields. The assignment of *M*_0_ − *M_t_* fields to *N* neurons follows a multinomial process, where the probability for neuron *i* is given by:

(41) $$\frac{u_{i,t}}{\sum_{j=1}^{N}u_{j,t}}$$

### Integration of Day‐to‐Day Fluctuations

The presence of neuron‐specific day‐to‐day fluctuations was assumed, which coordinately increase or decrease *P_t_* of all place fields of neuron *i*:

(42) $$P_{t}^{*}=P_{t}+\varepsilon_{\left(t,i\right)}\varepsilon_{\left(t,i\right)}∼\mathrm{Gaussian}\left(0,\sigma =0.1\right)$$

$P_{t}^{*}$, the corrected *P_t_*, was clipped within the interval [0,  1]. The introduction of such noise, which may arise from fluctuations in neuronal excitability or upstream inputs, is biologically plausible. Both the Gamma‐Poisson parameters α,  β, as well as the fluctuation parameter σ, can be adjusted to modulate the degree of coordination.

### Statistical Tests

The data were analyzed using custom MATLAB or Python codes. Statistics were calculated by SciPy or GraphPad Prism 8.0. Statistical analysis was performed as described in the figure legends. The exact sample size (n) for each experimental group was indicated in the figure legend or in the main text. If not otherwise indicated, error bars indicate 95% confidence interval.

## Conflict of Interest

The authors declare no conflict of interest.

## Author Contributions

C. C. and S. Y. contributed equally to this work and are co‐first authors. C.M. and Q.C. conceived this project and supervised the full process. C.M., Q.C., C.C., and S.Y. designed the experiments. C.C., S.Y., and S.C. collected the data. S.Y., C.C., J.T., S.C., A.L., Y.Y., Y.L., and Y.W. analyzed the data. S.Y., C.C., and C.M. wrote the paper with input from all authors.

## Supporting information



Supporting Information

## Data Availability

The data that support the findings of this study are available from the corresponding author upon reasonable request.;
