# Supplementary material for: Single‐Field Evolution Rule Governs the Dynamics of Representational Drift in Mouse Hippocampal Dorsal CA1 Region
Source: Adv Sci (Weinh). 2025 Nov 28;13(8):e09532. doi: 10.1002/advs.202509532 (PMC12884766; doi:10.1002/advs.202509532)
Supplement: Supplementary file 1 — Supporting Information [file ADVS-13-e09532-s001.docx]

Supporting Information

Single-field evolution rule governs the dynamics of representational drift in mouse hippocampal dorsal CA1 region.

Cong Chen, Shuyang Yao, Sihui Cheng, Jiayi Tian, Ang Li, Yusen Yan, Xiang Zhang, Yuanjing Liu, Yumeng Wang, Qichen Cao*, Chenglin Miao*


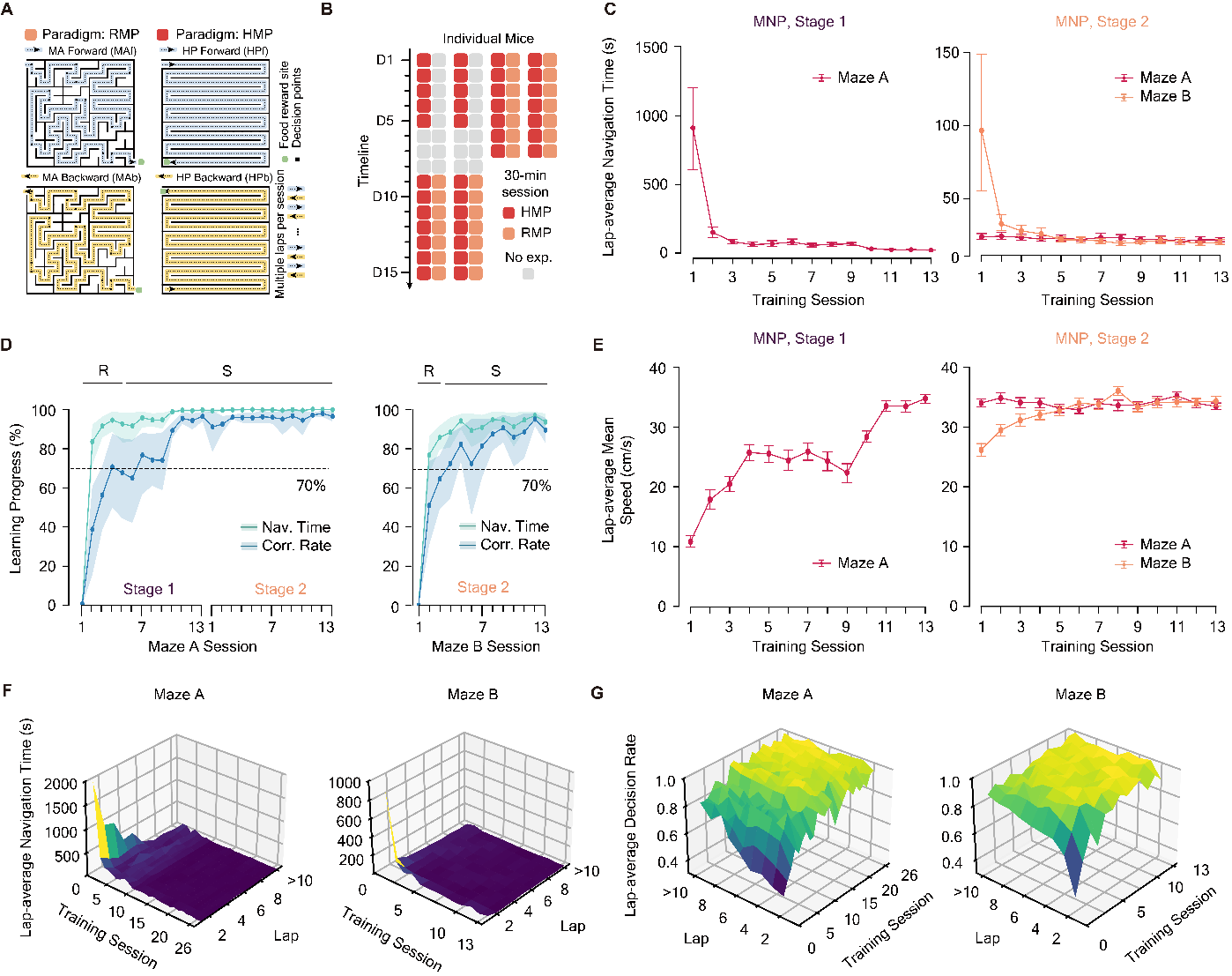


Figure S1. Robust maze learning process demonstrated by multiple behavioral indexes.

(**A**) Schematic of the reversed-maze paradigm (RMP, left column) and hairpin-maze paradigm (HMP, right column), showing moving directions (arrows), decision points (black squares), reward sites (green squares), and the linearized track of Maze A and hairpin maze (dotted line). Light blue represents information related to forward movement, while light yellow represents backward movement. Mice were trained to navigate back and forth between the entry and exit of Maze A for a total of 21-48 laps, with 10-24 laps in each direction. (**B**) The timeline of RMP and HMP training, with 7 HMP sessions for all four mice conducted before the RMP sessions within a day. Each vertical column relates to a mouse. (**C**-**G**) Additional behavioral indexes for maze navigation paradigm. (**C**) Lap-average navigation time for Stage 1 (left column) and Stage 2 (right column). (**D**) Learning progress in Maze A (left) and Maze B (right) assessed by lap-average navigation time (Nav. Time) and decision rate (Corr. Rate). The dashed line marks 70% behavioral progress. (**E**) Lap-average mean speed (cm/s) increased significantly during maze learning. Two-sided paired t-test, n = 6 mice; Maze A, from 10.9 ± 2.3 cm/s (Stage 1 S1) to 33.5 ± 1.1 cm/s (Stage 2 S13), P = 6 × 10^-6^. Maze B, from 25.6 cm/s (Stage 2 S1) to 34.2 ± 3.5 cm/a (Stage 2 S13), P = 0.011. (**F**-**G**) Mice showed robust learning across both laps and sessions, as measured by lap-average navigation time (**F**) and decision rate (**G**) Behavioral training. n = 6 mice; Error bars and bands represent 95% confidence intervals. Significance levels: ns, P ≥ 0.05; *, P < 0.05; **, P < 0.01; ***, P < 0.001; ****, P < 0.0001.


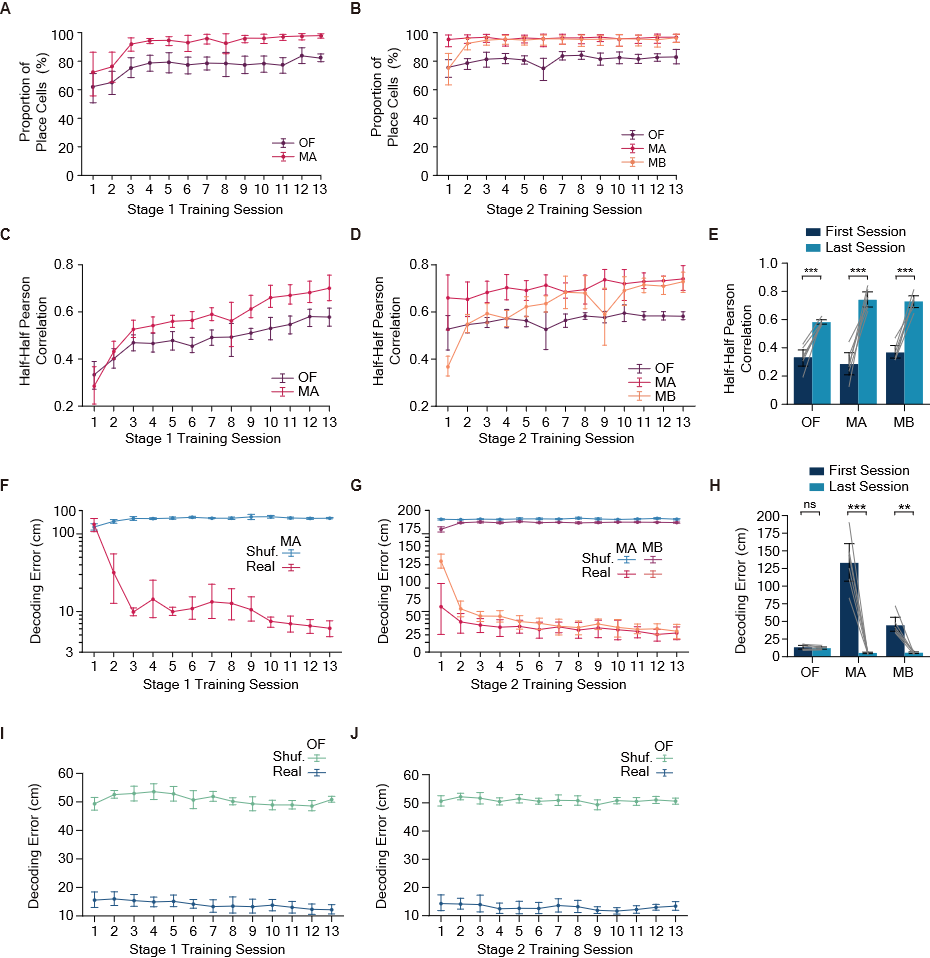


Figure S2. Within-session stability and coding accuracy of hippocampal spatial representations enhanced throughout maze learning process.

(**A**-**B**) Proportion of place cells in the open field (OF), Maze A (MA), and Maze B (MB) during Stage 1 (**A**) and Stage 2 (**B**). Place cell proportions from the two open-field sessions of each day are averaged. Proportions from the two open-field sessions of each day were averaged. Place-cell proportions in mazes were consistently higher than in the open field (n = 6 mice, two-sided paired t-test, 0.039 > P > 6 × 10^-6^ for all maze vs. OF comparisons, except Stage 1 S1 for MA, P = 0.11; and Stage 2 S1 for MB, P = 0.85). (**C**-**D)** Within-session stability of place cells, measured as the half-half session correlation, significantly increased during Stage 1 (**C**) and Stage 2 (**D**) in both complex mazes and the open field. (**E**) Summary of changes (n = 6 mice, two-sided paired t-test: MA, from 0.29 ± 0.10 (Stage 1 S1) to 0.74 ± 0.07 (Stage 2 S13), P = 0.0008; MB, from 0.37 ± 0.05 (Stage 2 S1) to 0.73 ± 0.05 (Stage 2 S13), P = 0.00012; and the open field, from 0.33 ± 0.07 (Stage 1 S1) to 0.58 ± 0.02 (Stage 2 S13), P = 0.0006). Eventually, half–half correlations in mazes exceeded those in OF (two-sided paired t-test with Bonferroni correction, Stage 2 S13: MA vs. OF, P = 0.002; MB vs. OF, P = 0.002; MA vs. MB, P = 0.54. Each correlation was averaged across the place-cell population. (**F**-**J**) Position decoding by a modified Naïve Bayesian Classifier (Methods). Training set: 80% of frames; 5-fold cross-validation applied. Representative decoding results for mazes in Stage 1 (**F**) and Stage 2 (**G**), as well as for OFs (**I**, **J**). Training set size: 80% of all frames in a session. 5-fold cross-validation was performed, and decoding losses were averaged. (**H**) Decoding error (unit: cm) showed a significant decline in complex mazes (n = 6 mice, two-sided paired t-test: MA, 132.9 ± 33.3 cm (Stage 1 S1) to 5.3 ± 1.3 cm (Stage 2 S13), P = 0.0003; MB, 44.5 ± 13.0 cm (Stage 2 S1) to 5.6 ± 1.5 cm (Stage 2 S13), P = 0.0013) but remained unchanged in the open field (two-sided paired t-test: 15.6 ± 4.8 cm (Stage 1 S1) to 13.4 ± 2.7 cm (Stage 2 S13), P = 0.09). Shuf.: Decoding positions over testing data and matching them with shuffled testing labels. Real: Decoding results based on actual data. Error bars represent 95% confidence intervals. Significance levels: ns, P ≥ 0.05; *, P < 0.05; **, P < 0.01; ***, P < 0.001; ****, P < 0.0001. In this panel, data from all within-lap neural activities of place cells were used. For each indicator, the two open field sessions per block were averaged for paired t-tests. Data presented as mean ± std. Bonferroni correction was applied for multiple comparisons across open field (OP), Maze A (MA), and Maze B (MB).

**

**

Figure S3. Example place cells in open field and complex mazes.

**(A-B)** Representative place cells with either single or multiple fields recorded in the open field (**A**) and both mazes (**B**). Each cell is entitled with mouse ID, environment, recorded session (S), cell index, and spatial information (SI; unit: bits/spike). (**A**) For each cell in the open field: **left**, event rate map (Peak: peak event rate in Hz); **right**, spatial distribution of calcium events (black dots) and mouse trajectories (gray lines). (**B**) For each cell: **top left**, event rate map (Peak: peak event rate in Hz); **bottom left**, spatial distribution of calcium events (black dots) and mouse trajectories (gray lines); **top right**, linearized rate map along the correct track; **bottom right**, raster-like plot with calcium events (red bars) aligned to linearized mouse trajectories (gray lines) along the correct track. Shaded areas of the same color mark the ranges of the same place fields. Calcium activities on incorrect tracks and backward movements are excluded.





Figure S4. Multi-field place cells were prevailing in other spatial maps

(**A-E**) Results from the reverse maze paradigm (RMP). (**A**) 3 example place cells. For each cell: **left**, event rate maps for forward (**top left**) and backward (**bottom left**) motions, respectively; middle, spatial distributions of calcium events (black dots) and mouse trajectories (gray lines) for forward (**top middle**) and backward (**bottom middle**) motions, respectively; **top right**, linearized rate map along the correct track; **bottom right**, raster-like plot with calcium events (red bars) aligned to linearized mouse trajectories (gray lines) along the correct track. Shaded areas marked the range of place fields from either forward (blue) or backward (yellow) map. (**B**) The proportion of place cells encoding both directions, with overlapped colored squares indicating that 81.8 ± 10.9% of place cells encode both directional movements. (**C**) The proportion of place cells in backward directional movement (85.7 ± 8.5%) is mildly but significantly lower than that of forward movement (Paired t-test, n = 4 mice, df = 37: P = 7 × 10^-12^), possibly due to distinct levels of familiarization. (**D**) Place cells encoding both directions display multiple place fields at the ensemble level, (**E**) with the average number of fields for backward direction (7.3 ± 1.8) mildly but significantly greater than that for forward direction (6.4 ± 1.7, Paired t-test, n = 4 mice, df = 37: P = 4.4 × 10^-8^). This suggests that the total number of fields to encode both directions remains relatively constant. Calcium events detected on incorrect paths were excluded. (**F**-**K**) Results from the hairpin maze paradigm. (**F**) Three example place cells recorded in the hairpin maze. (**G**) The proportion of place cells encoding both directions, with overlapped colored squares indicating that 62.7 ± 15.4% of place cells encode both directional movements. (**H**) The proportion of place cells encoding both the forward (76.7 ± 9.9%) and backward (73.8 ± 14.8%) movements showed no significant difference (Paired t-test, n = 4 mice, df = 27: P = 0.054). (**I**) The proportion of place cells encoding both directions gradually increased over time (Paired t-test, n = 4 mice, forward: from 65.3 ± 7.8% (S1) to 83.2 ± 4.2% (S7), P = 0.005; backward: from 62.1 ± 10.6% (S1) to 83.2 ± 8.7% (S7), P = 0.013). (**J** to **K**) Place cells in the hairpin maze displayed evident multi-field coding, with the average number of fields for the forward direction (8.6 ± 2.6) being mildly but significantly greater than that for the backward direction (8.0 ± 2.2, Paired t-test, n = 4 mice, df = 27: P = 2.3 × 10^-5^). Error bars: 95% confidence intervals. Significance levels: ns, P ≥ 0.05; *, P < 0.05; **, P < 0.01; ****, P < 0.0001. All open field sessions were included for visualization, but for statistical analysis, the two open field sessions per block were averaged for paired t-tests.


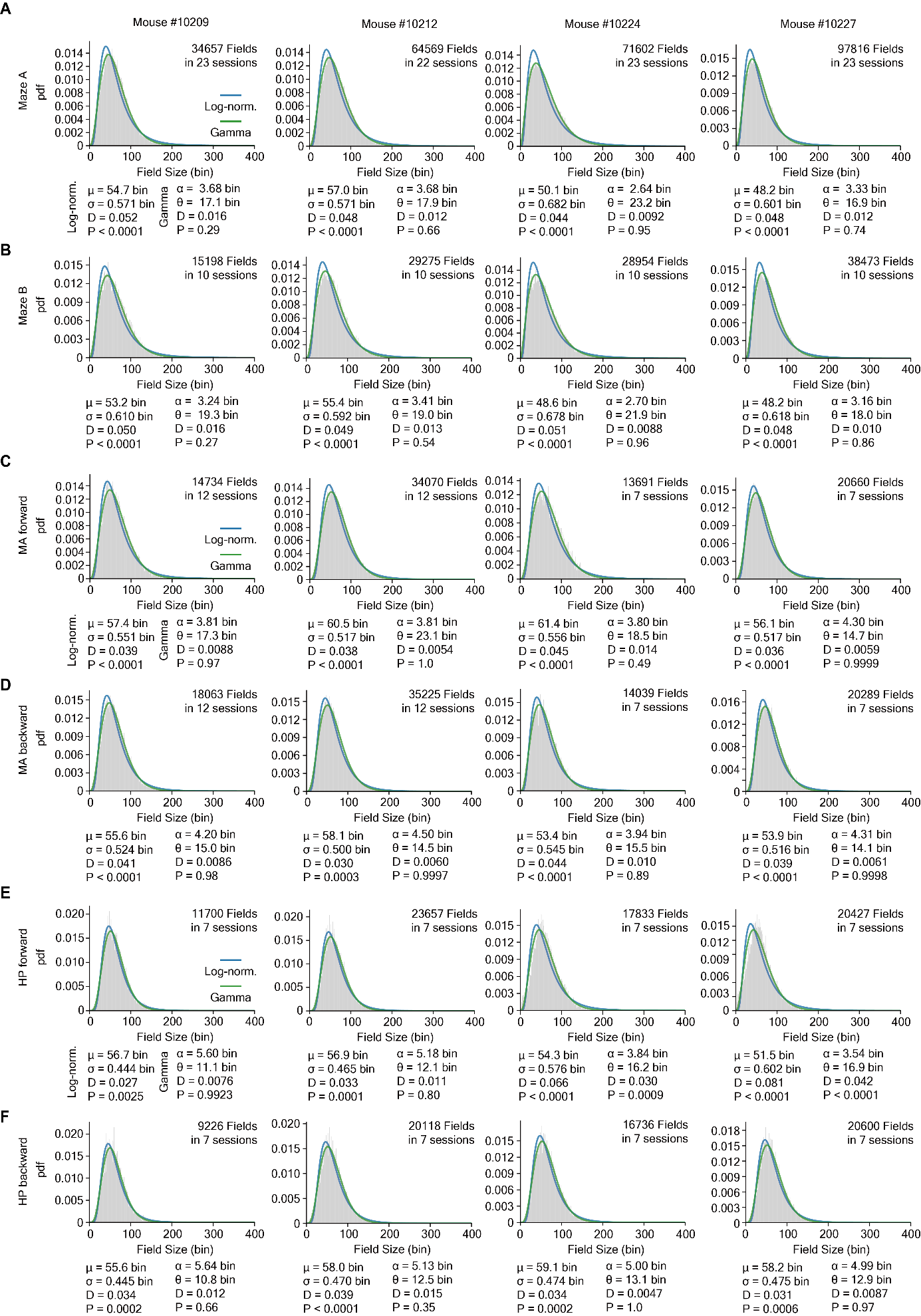


Figure S5. Field sizes of place fields in goal-directed navigation paradigm are well-described by Log-normal and Gamma distributions.

(**A**-**F**) Field sizes (unit: bins, each bin is 4 cm²) recorded in spatial maps MA (**A**), MB (**B**), MAf (**C**), MAb (**D**), HPf (**E**), and HPb (**F**), were well-fitted by skewed distributions (e.g., gamma and gamma distributions). The goodness of fit for log-normal and gamma distributions was assessed by Lilliefors-corrected Kolmogorov-Smirnov tests (see Supporting Information). The location parameters for both distributions were forcibly set to 0. Gamma distribution provides better estimation for most situations (P > 0.27 for 22 of 24 spatial maps, KS statistic D = 0.012± 0.008), compared to Log-normal distribution (P < 0.003 for all 24 spatial maps, KS statistic D = 0.044 ± 0.012, which is significantly greater than that of gamma distribution, Paired t-test P = 4 × 10^-18^). μ and σ represent the mean and standard deviation of the log-normal distribution, respectively. α and θ indicate the shape and scale parameters of the gamma distribution, respectively. D: KS statistic; P: P-values determined by Monte Carlo simulation. Pdf: probability density function of field sizes. Gray bars: histogram of actual field size distribution. Blue and green lines indicate the pdfs of fitted log-normal and gamma distributions, respectively. MA: Maze A; HP: Hairpin maze. Novel sessions of maze navigation paradigm (Maze: Stage 1 S1 to 3; Maze B: Stage 2 S1 to 3) were excluded from analysis.


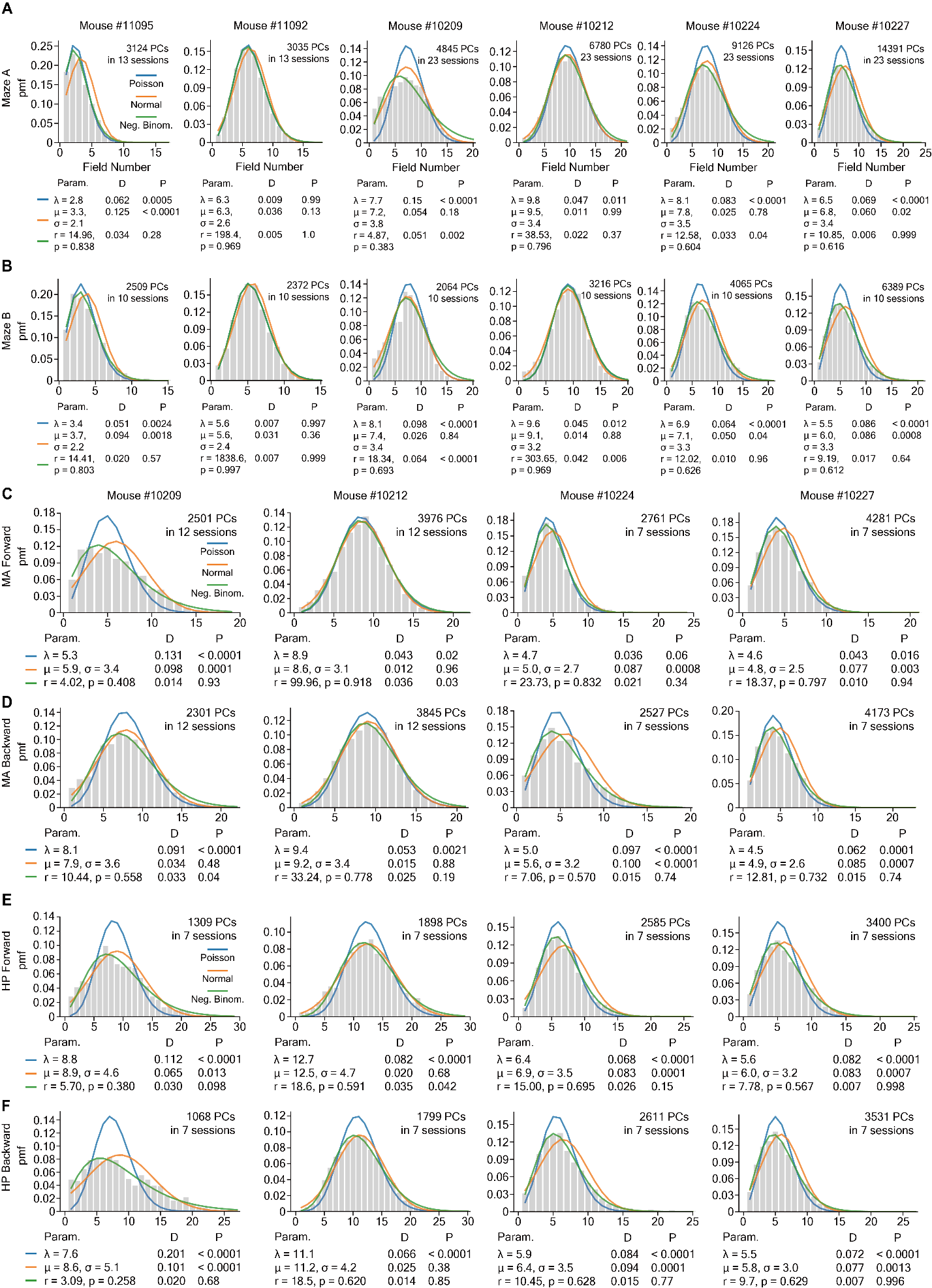


Figure S6. Field number per place cell in goal-directed navigation paradigm are well-described by negative binomial distribution but with a Poisson-like shape.

**(A** to **F**) The number of fields per place cell recorded in spatial maps in spatial maps MA (**A**), MB (**B**), MAf (**C**), MAb (**D**), HPf (**E**), and HPb (**F**), were well-fitted by negative binomial and discrete normal distributions but poorly fitted by the Poisson distribution. The goodness of fit for each distribution was assessed by Lilliefors-corrected Kolmogorov-Smirnov tests (see Supporting Information). The negative binomial distribution provides the best estimation for most situations (P > 0.09 for 19 of 26 spatial maps and P > 0.03 for 24 of 26 spatial maps, KS statistic D = 0.023± 0.014), compared to the normal distribution (P > 0.05 for 12 of 26 spatial maps, KS statistic D = 0.060 ± 0.033, which is significantly greater than that of the negative binomial distribution, Paired t-test with Bonferroni correction: P = 1.2 × 10-4) and the Poisson distribution (P > 0.05 for 3 of 26 spatial maps, KS statistic D = 0.075 ± 0.039, Paired t-test with Bonferroni correction: P = 2.2 × 10-7). λ is the rate parameter of the Poisson distribution; μ and σ represent the mean and standard deviation of the normal distribution, respectively; r and p indicate the shape and scale parameters of the negative binomial distribution, respectively. D: KS statistic; P: P-values determined by Monte Carlo simulation. pmf: probability mass function of the field number per place cell. Gray bars: histogram of the actual field size distribution. Blue, orange, and green lines indicate the pmfs of fitted Poisson, normal, and negative binomial distributions, respectively. Param.: parameters; PC: place cells; MA: Maze A; HP: Hairpin maze. Novel sessions of the maze navigation paradigm (Maze A: Stage 1 S1 to S3; Maze B: Stage 2 S1 to S3) were excluded from analysis.


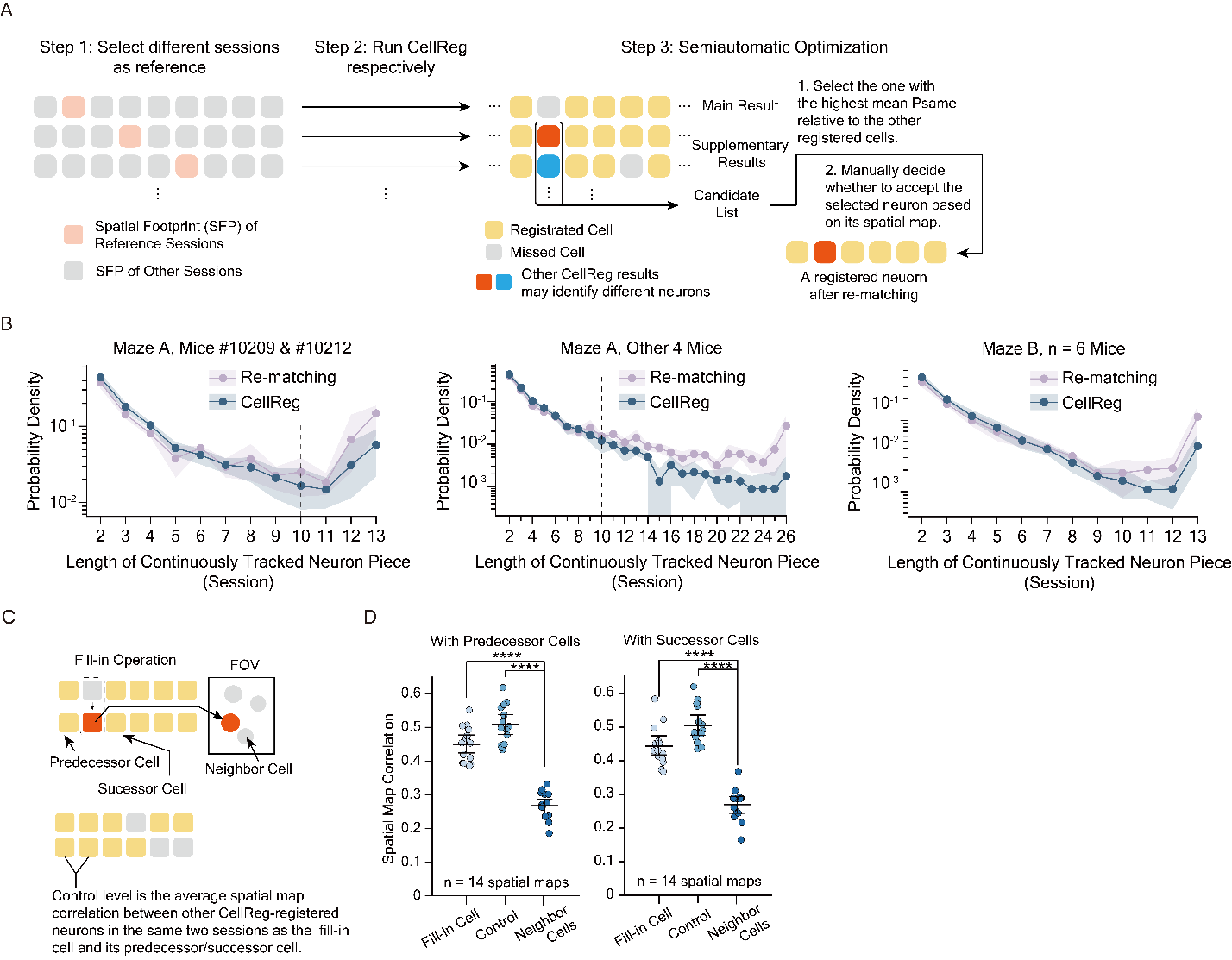


Figure S7. Re-matching strategy increases the number of continuously tracked neuron pieces while maintaining functional accuracy comparable to CellReg.

(**A**) Schematic for re-matching strategy. Briefly, the re-matching process involves selecting different reference SFPs (**Step 1**), running CellReg with each (**Step 2**), and designating one output as the main result while treating the others as supplementary (**Step 3**). For a missed position within a registered neuron from the main result, we searched the supplementary outputs for candidate neurons suitable for filling that position. The best candidate was then automatically selected based on an optimized registration score (**Step 3-1**; Supporting Information) and manually confirmed according to its spatial rate map (**Step 3-2**). (**B**) Re-matching significantly increased the proportion of continuously tracked neuron pieces (defined in Methods) with lengths ≥ 10, from 8.1 ± 3.9% (mean ± s.d.) to 18.1 ± 6.6% (two-sided paired *t*-test, *P* = 4 × 10^-6^, *n* = 14 spatial maps from 6 mice). Only these long pieces were used in the analysis, simulations, and modeling of SFER. (**C**) Schematic of cell classes used to assess the functional accuracy of re-matching. Predecessor cell: the nearest CellReg-registered neuron occurring before the fill-in neuron. Successor cell: the nearest CellReg-registered neuron occurring after the fill-in neuron. Neighbor cell: the nearest cell in the same FOV as the fill-in/place-in cell. If the fill-in cell is in Session $S_{\mathrm{curr}}$, its predecessor and successor cells are in sessions $S_{\mathrm{pre}}$ and $S_{\mathrm{suc}}$, respectively. (**D**) Spatial map correlations confirm the functional accuracy of re-matching. We computed correlations between fill-in cells and their predecessor (**left**) or successor (**right**) neurons. Control levels were defined as the average correlation between CellReg-registered neurons in $S_{\mathrm{curr}}$ and either $S_{\mathrm{pre}}$ (**left**) or $S_{\mathrm{suc}}$ (**right**). Chance levels were determined by correlations between the predecessor/successor cell and the neighbor of the fill-in cell. Two-sample paired *t*-tests assessed significance. Significance levels: ****, P < 0.0001.


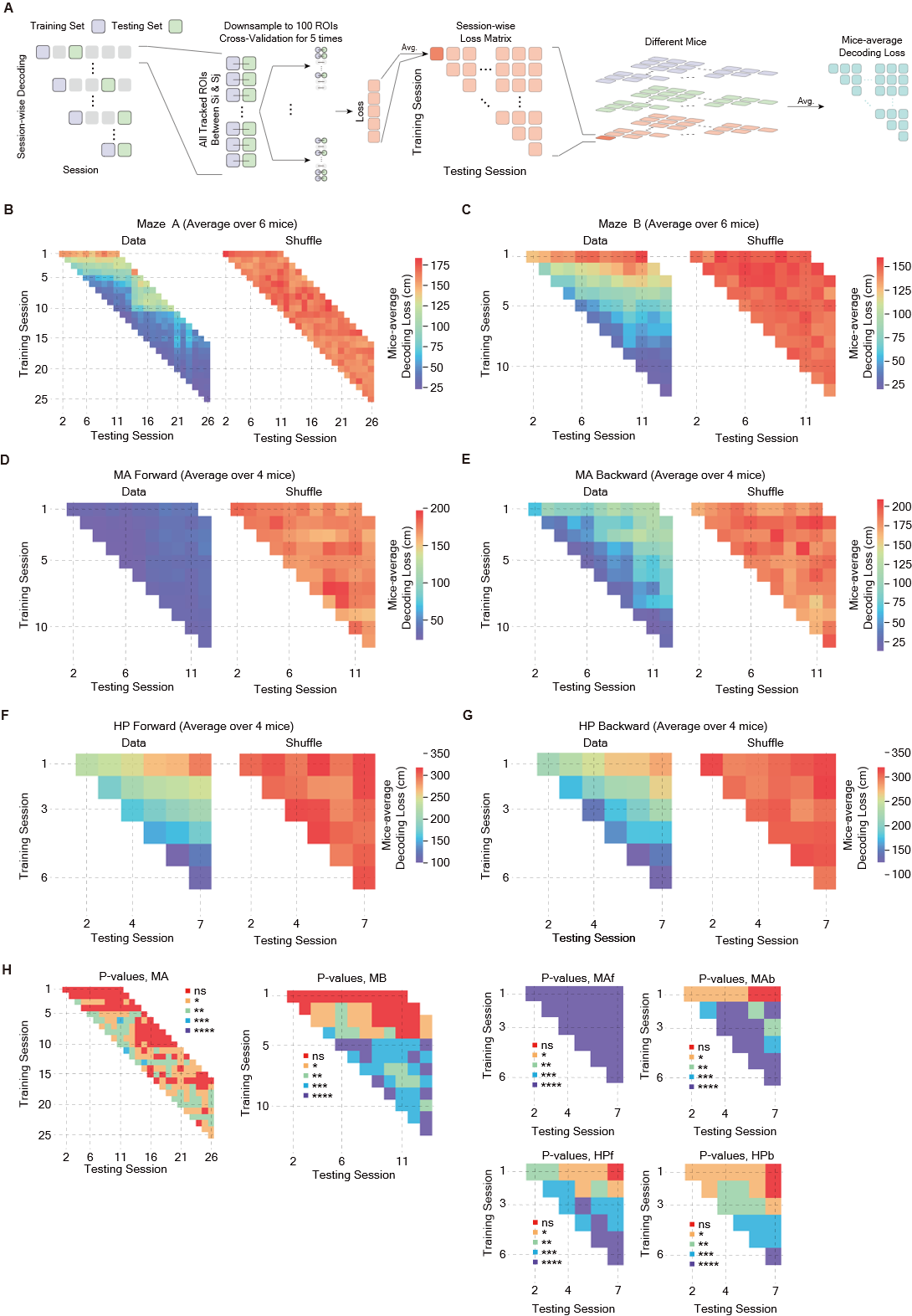


Figure S8. Cross-session decoding validates the functional consistency of cell tracking.

(**A**) Schematic for cross-session decoding. For any two sessions $s_{i}$ and $s_{j}$ separated by fewer than 10 sessions, a naïve Bayes classifier was trained on data from $s_{i}$ and tested on $s_{j}$. Five cross-validations were performed per session pair, with both training and testing sets downsampled to 100 randomly selected neurons. The averaged values generated a session-wise decoding loss matrix for each spatial map, and results across mice were further averaged and visualized in (**B–G**). (**B-G**) Average decoding losses for spatial maps MA (**B**), MB (**C**), MAf (**D**), MAb (**E**), HPf (**F**), and HPb (**G**). For each map: **left**, results from actual data; **right**, results from shuffled data. (**H**) Two-sided two-sample t-tests compared actual and shuffled results, with n = 6 mice for MA and MB, and n = 4 mice for MAf, MAb, HPf, and HPb. Significance levels: ns, P ≥ 0.05; *, P < 0.05; **, P < 0.01; ***, P < 0.001; ****, P < 0.0001.


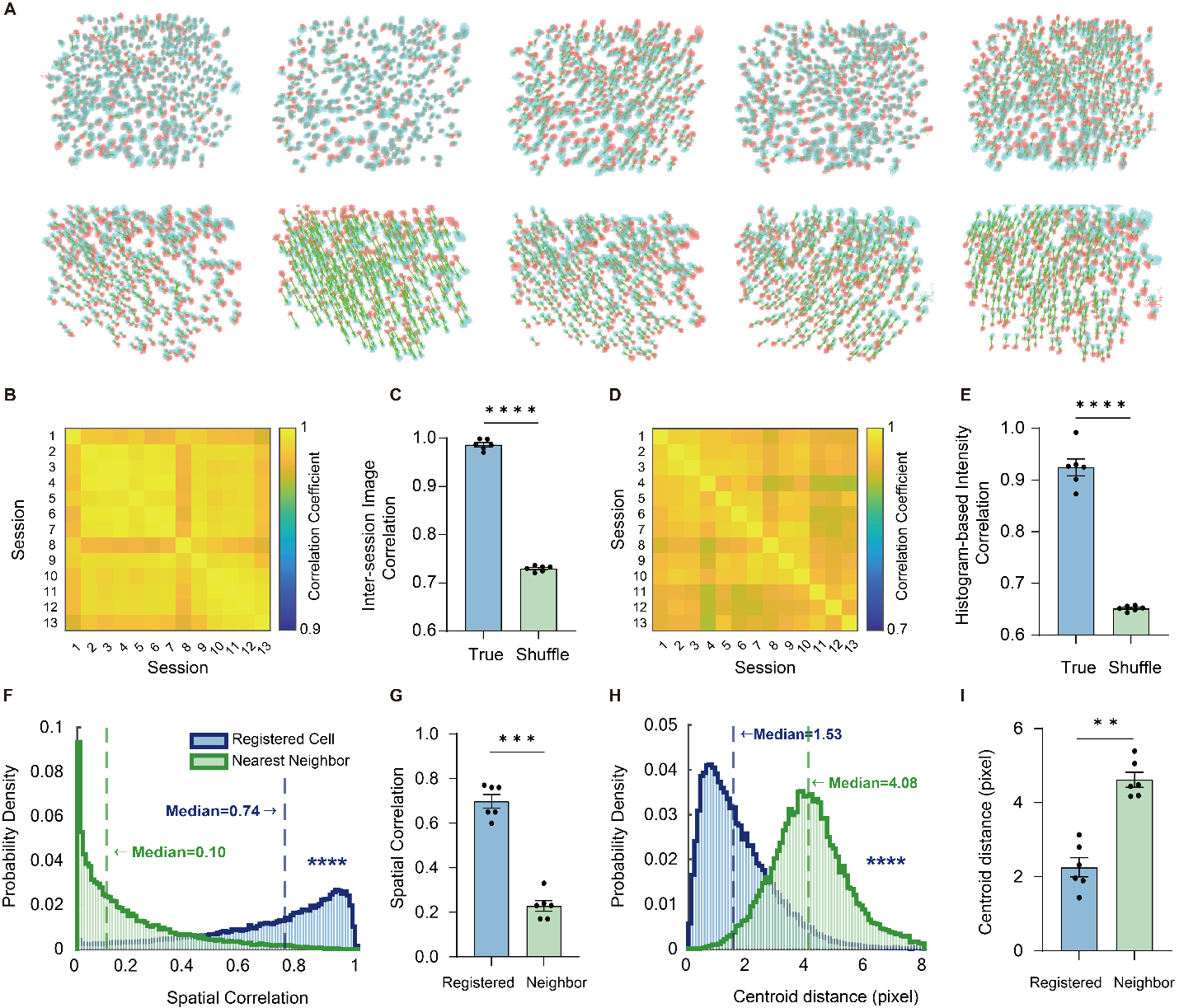


Figure S9. Validation of longitudinal cell registration in Miniscope imaging.

(**A**) Example spatial footprint (SFP) displacement vector fields after CellReg-based manually registration, which shows systematic shifts with stable relative positions. (**B**) Inter-session image correlation matrix for all mice (n=6), indicating consistent imaging focal plane across days. (**C**) The similarity of the cross-day imaging FOV is significantly higher than that of shuffled data. A session-shuffle (permutation) test was performed by randomly reassigning session identities across mice while preserving the number of sessions per mouse. This procedure was repeated 100 times, and the cross-day FOV correlation was calculated to obtain the average value. n=6, paired t-test, **** p < 0.0001. (**D**) Histogram-based intensity correlation matrix for all mice (n=6), further confirming stable imaging plane. (**E**) Histogram-based intensity values are highly similar when compared with shuffled data, using the same shuffle method as in (C). n=6, paired t-test, **** p < 0.0001. (**F**) Example (Mice #10232) distribution of spatial footprint correlations for CellReg-based manually registered cell pairs and nearest neighbors (excluding matched pair). Kolmogorov-Smirnov test, **** p < 0.0001. (**G**) Registered cells exhibited significantly higher spatial correlations than nearest neighbors, serving as controls. n=6, paired t-test, *** p < 0.001. (**H**) Example (Mice #10232) distribution of centroid distances for registered cell pairs and nearest neighbors (excluding matched pair). Kolmogorov-Smirnov test, **** p < 0.0001. (**I**) Registered cells showed significantly smaller centroid distance than nearest neighbors, confirming robust CellReg-based registration combined with manual verification. n=6 mice, paired t-test, ** p < 0.01


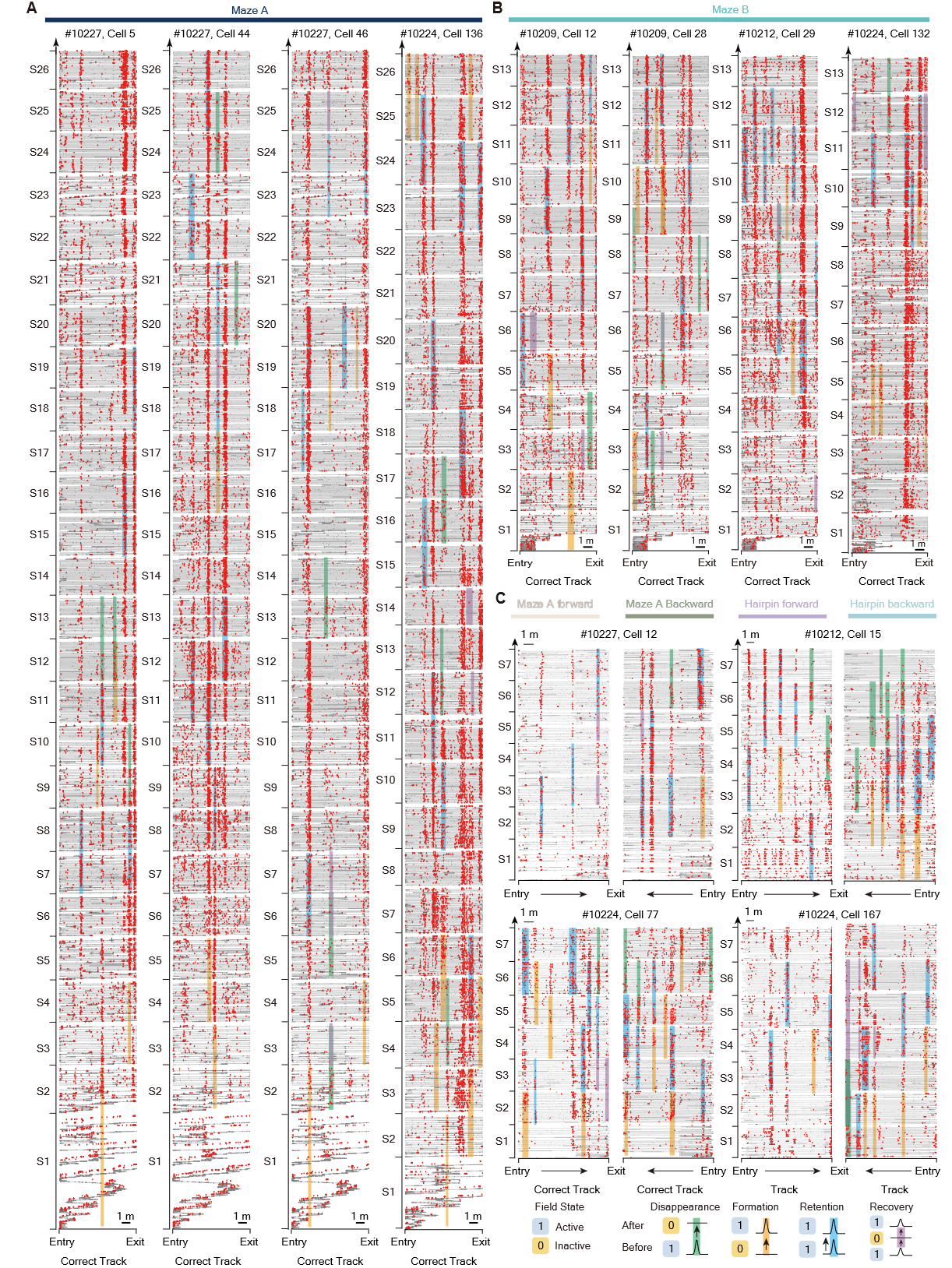


Figure S10. Examples long-term tracked place cells in multiple spatial maps

(**A-C**) 12 exemplary place cells demonstrate a spectrum of complex evolution of place fields in their spatial maps, which were widely recorded across distinct paradigms and spatial maps. Three elementary evolutionary events—disappearance, formation, and retention—are highlighted with green, yellow, and blue shading, respectively. We only selectively marked some evolutionary events. Purple shading indicates continuous dormancy periods of fields that later recovered. Only a proportion of representative events are marked. Red bars, calcium events; gray lines, mouse trajectories on the correct track. Sessions are labeled as S1 to S26 (corresponding to Stage 2 S13).


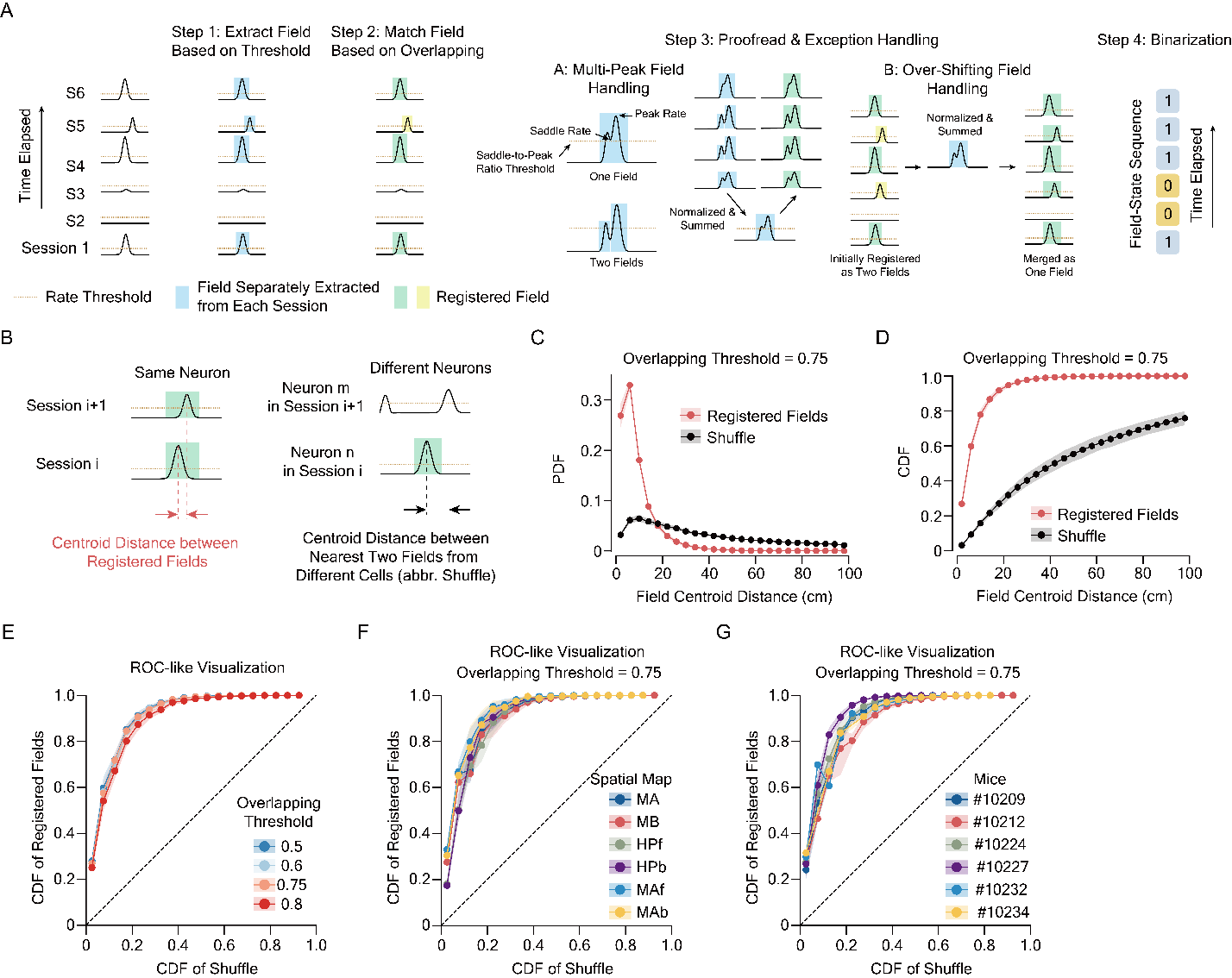


Figure S11. Overlap-based registration of place fields across multiple sessions.

(**A**) Schematic of multi-session field registration (see Methods). Place fields of each neuron were first extracted in individual sessions (**Step 1**). Fields across sessions of the same registered neuron were then matched based on an overlap threshold (default 0.75, i.e., 75% overlap; **Step 2**). Additional steps addressed two-peak fields and determined whether to register shifted fields together based on the all-session–summed event rate (**Step 3**). The final outputs were binarized into field-state sequences (**Step 4**). (**B**) Schematic of centroid distances for registered fields (left) and shuffled controls (right). Shuffled controls were generated by randomly pairing two neurons $(i,j)$ across sessionsand identifying for the nearest fields in Cell $j$ for each field of Cell $i$. (**C-D**) Probability density function (PDF, **C**) and cumulative density function (CDF, **D**) of centroid distances, showing limited distances for registered fields (mean ± s.d. of 95% CI: 23.4 ± 5.0 cm; *n* = 30 spatial maps from 6 mice). (**E**) ROC-like visualization of centroid distances across different overlap thresholds (0.5–0.8), demonstrating robustness of the registration method. (F) ROC-like visualization across spatial maps, showing method robustness across spatial maps. (G) ROC-like visualization across animals, showing robustness across individual mice. Error bands: 95% confidence intervals. *n* = 30 spatial maps from 6 mice.”


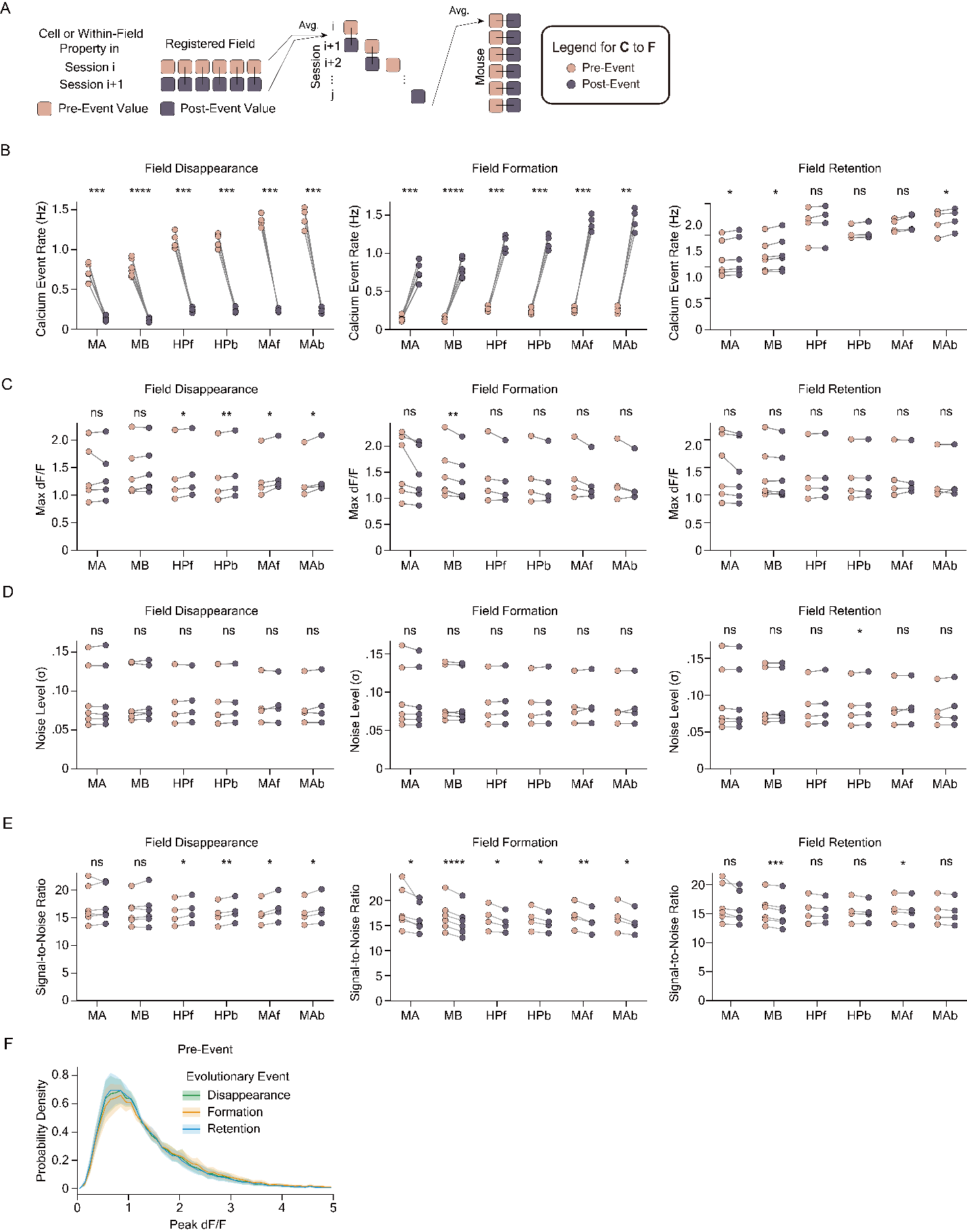


Figure S12. Evolutionary events were not driven by systematic changes in cellular noise level or signal strength.

(**A**) Schematic of the analysis of cellular properties across evolutionary events. For each event, property, spatial map, and pair of consecutive sessions, field-average pre- and post-event values were computed. These were then averaged across sessions to yield a session-average value per mouse per spatial map. This approach ensures statistical inference at the animal level without inflating the degrees of freedom. (**B**) Changes in peak event rate (Hz) within place fields for disappearance (**left**), formation (**middle**), and retention (**right**) events. (**C**) Changes in peak calcium traces (ΔF/F) of cells—including activity detected outside fields and during inter-lap intervals—for disappearance (**left**), formation (**middle**), and retention (**right**) events. (**D**) Changes in noise level for disappearance (**left**), formation (**middle**), and retention (**right**) events. Noise level was measured as the standard deviation of the lowest 97.5% ΔF/F values, reflecting each cell’s baseline activity. (**E**) Changes in signal-to-noise ratio (SNR) for disappearance (**left**), formation (**middle**), and retention (**right**) events. (**F**) Distribution of pre-event peak ΔF/F across the three event types, showing no systematic bias in cellular properties.


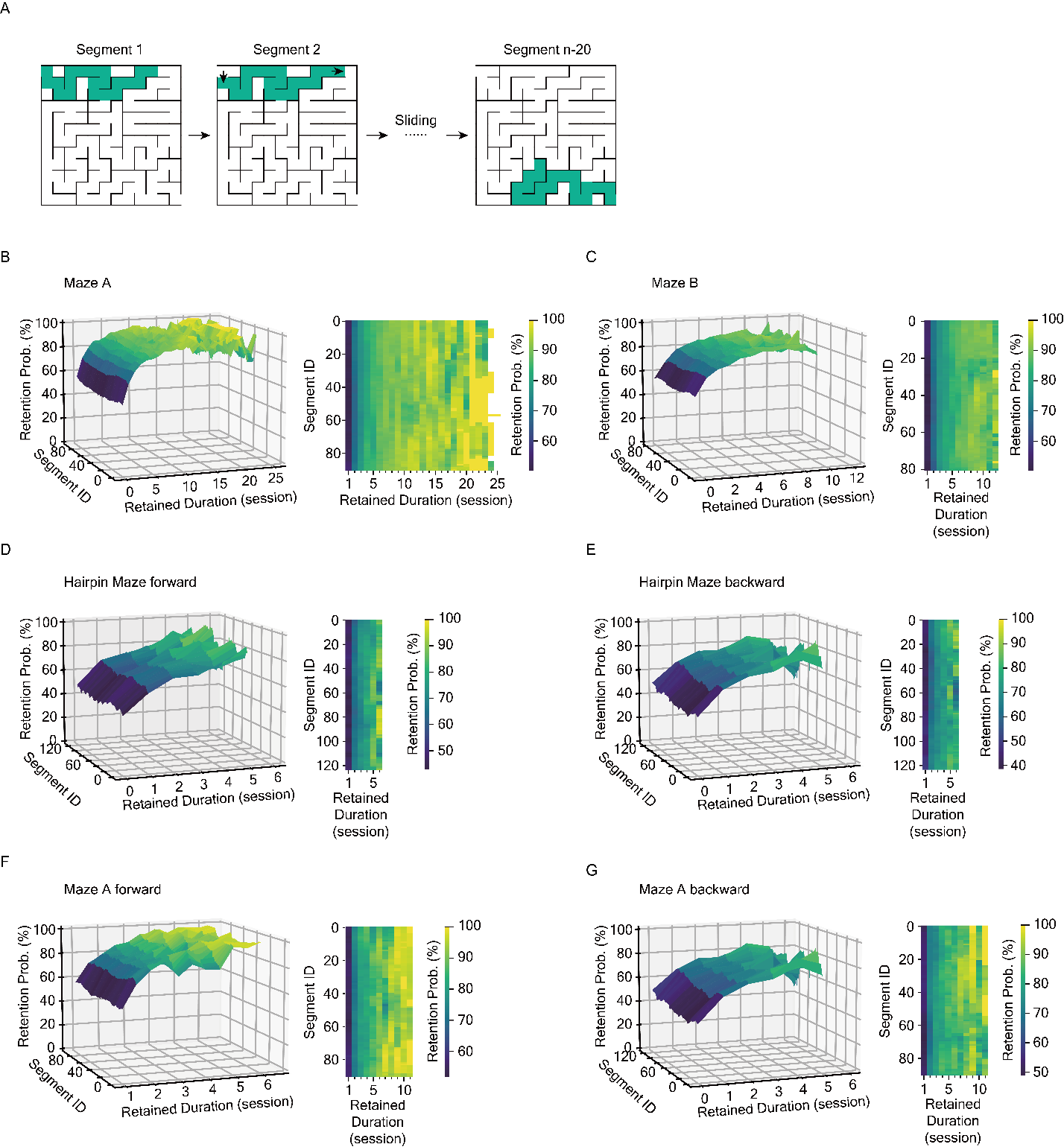


Figure S13. Retention probability is position-irrelevant

(**A**) A 1.6-m spatial window (green region) was slid along the main track of the mazes with a step length of 8 cm. Place fields located within this subregion of the mazes were included for calculating retention probability. (**B**-**G**) The retention probability calculated remains relatively consistent across the maze track. Fluctuations observed may be attributed to the limited number of field samples within a subregion compared to the entire maze.


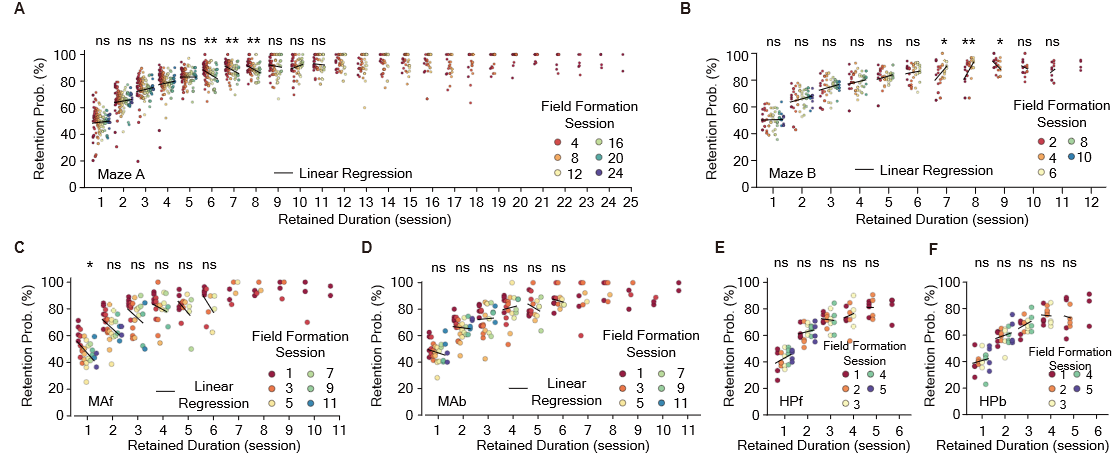


Figure S14. Novelty-irrelevant properties of retention probability.

(**A**-**F**) Retention probability computed for place fields according to their formation session in spatial maps MA (**A**), MB (**B**), MAf (**C**), MAb (**D**), HPf (**E**), and HPb (**F**). Results are shown as circles, with colors corresponding to field formation sessions. For Maze A and B, where dozens of groups exist, only selected colors are displayed in the legend for clarity. Black line: linear regression of retention probability against formation session. Wald’s test was used to determine whether the regression slope significantly deviated from zero. Significance levels: ns, P ≥ 0.05; *, P < 0.05; **, P < 0.01; ***, P < 0.001; ****, P < 0.0001.


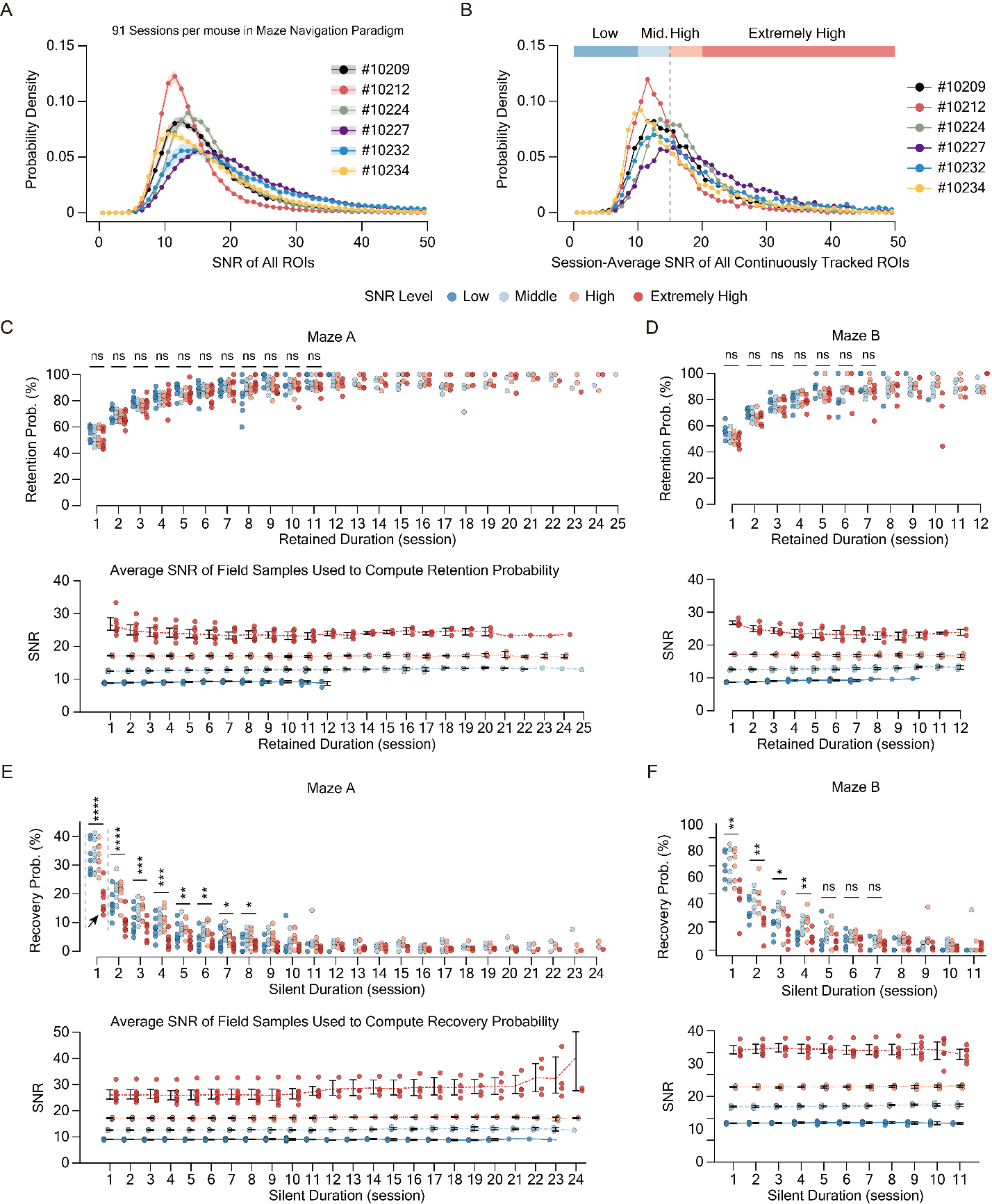


Figure S15. Retention probability is unaffected by neuronal SNR, whereas neurons with extremely high SNR exhibit reduced recovery probability.

(**A**) Distribution of SNR for all ROIs per session (n = 91 sessions per mouse). (**B**) Distribution of average SNR for all registered neurons across spatial maps of individual mice. Top colored bands indicate different SNR levels, ensuring comparable sample sizes across groups. Gray vertical dotted lines: boundaries of SNR levels. (**C,D**) Top: Retention probability in MA (**C**) and MB (**D**), computed separately for neurons at different average SNR levels. Bottom: mean neuronal SNR for each group. (**E,F**) Top: Recovery probability in MA (**E**) and MB (**F**), computed separately for neurons at different average SNR levels. Arrows highlight that neurons with extremely high SNR show significantly lower recovery probability. Bottom: mean neuronal SNR for each group. One-way ANOVA was used to test whether SNR significantly affects either retention or recovery probability. Significance levels: ns, P ≥ 0.05; *, P < 0.05; **, P < 0.01; ***, P < 0.001; ****, P < 0.0001.


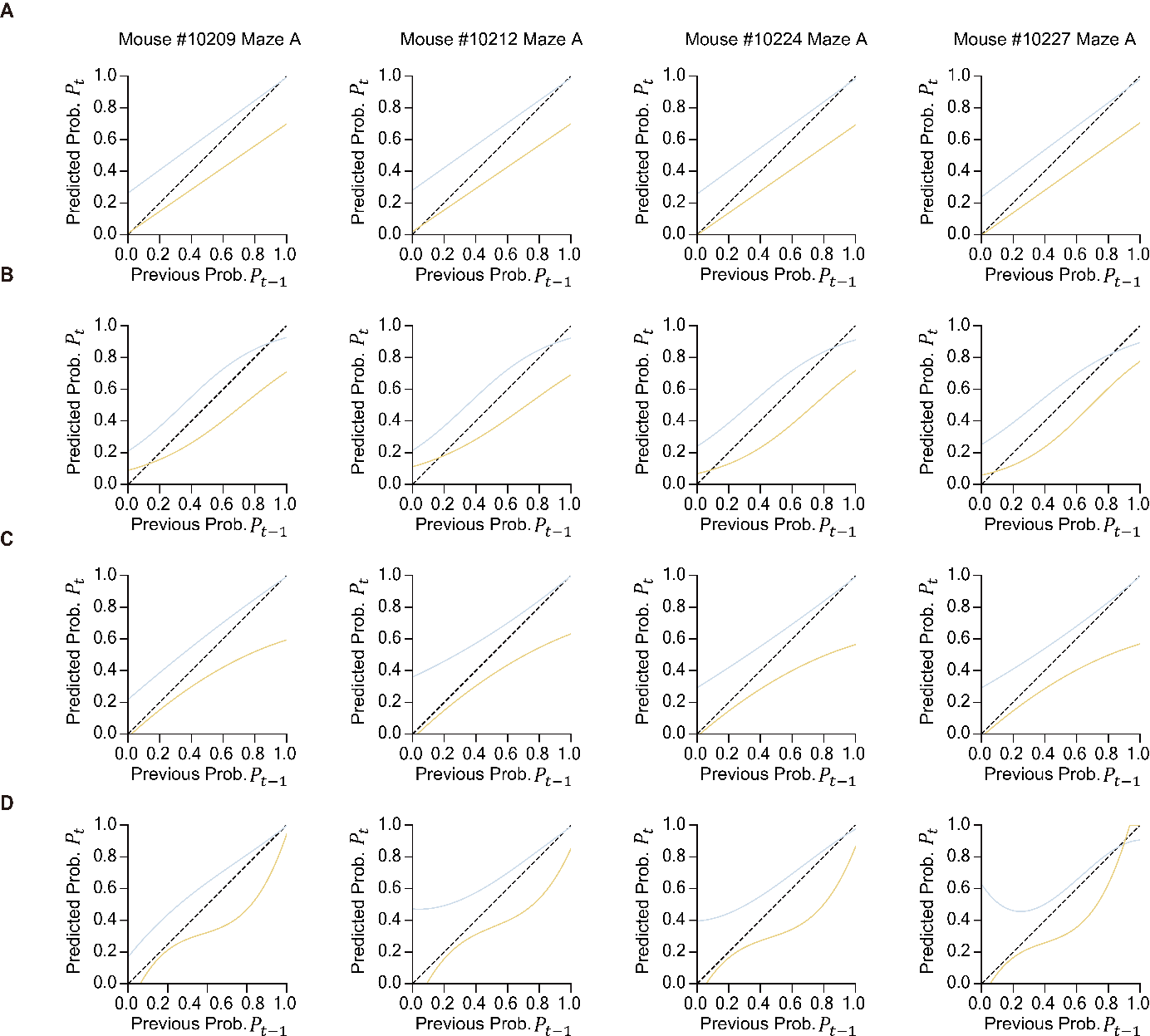


Figure S16. Four types of functions selected as candidates for SFER in the general sense.

The formulas for these functions can be found in the Methods section under “**IV: SFER in the general sense.**” (**A**) Linear function. (**B**) Logistic function. (**C**) Quadratic function. (**D**) Cubic function. Blue lines represent the functions that incrementally update $P_{t}$, while yellow lines depict those that decrease $P_{t}$. Examples from the spatial map MA.


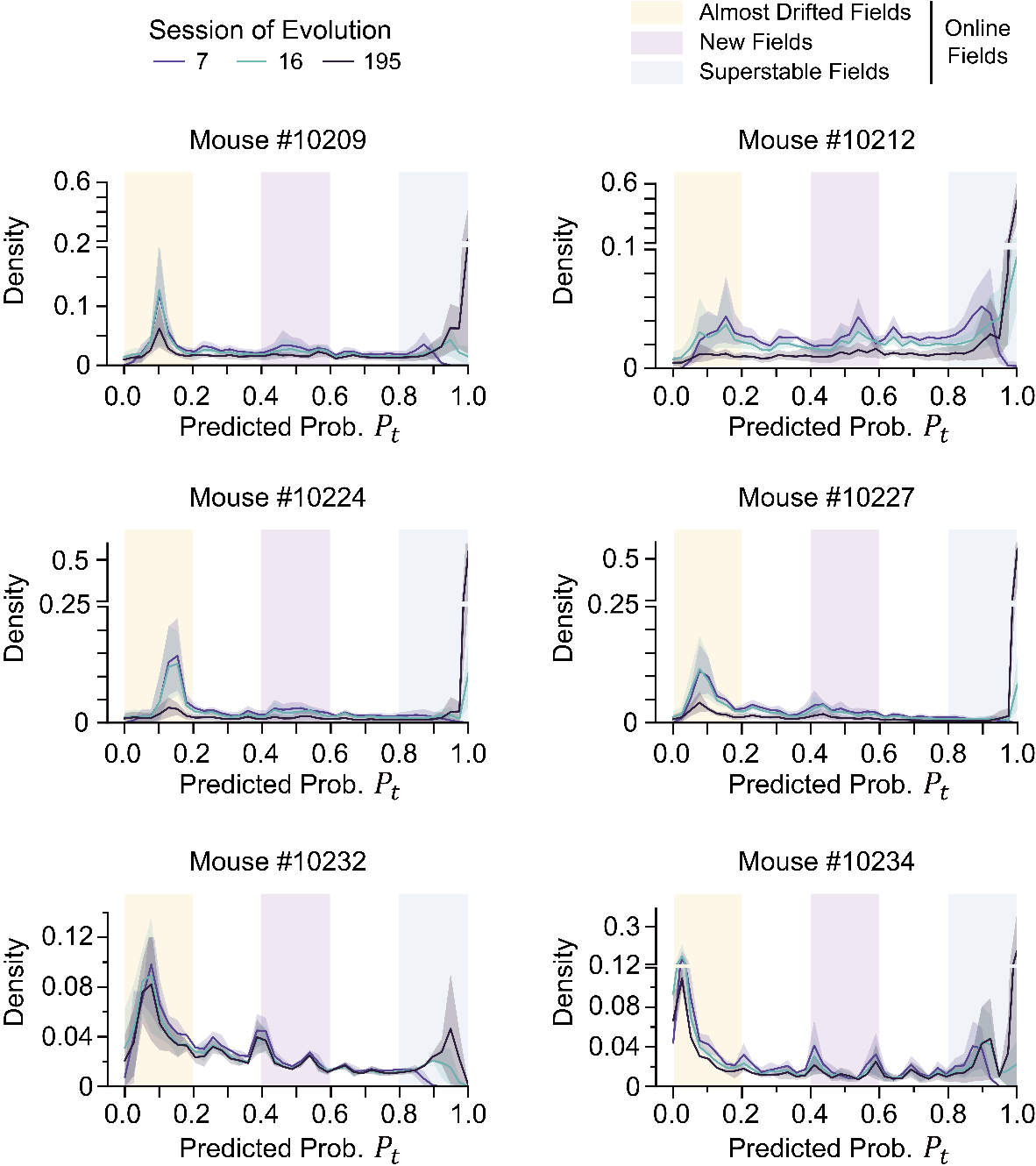


**Figure S17 Dual-fate dynamics for each mouse separately.**

Probability density function of$P_{t}$ in three representative simulated sessions (Sessions 7, 16, and 195). Background colored shading marks the three peaks of the $P_{t}$ distribution, indicative of dual-fate dynamics. Data from six mice were analyzed independently, with SFER models fitted separately to each spatial map (MA and MB for mice #10232 and #10234; MA, MB, MAf, MAb, HPf, and HPb for the remaining mice) and presented collectively. Simulations were performed for 10,000 place fields over 200 sessions.


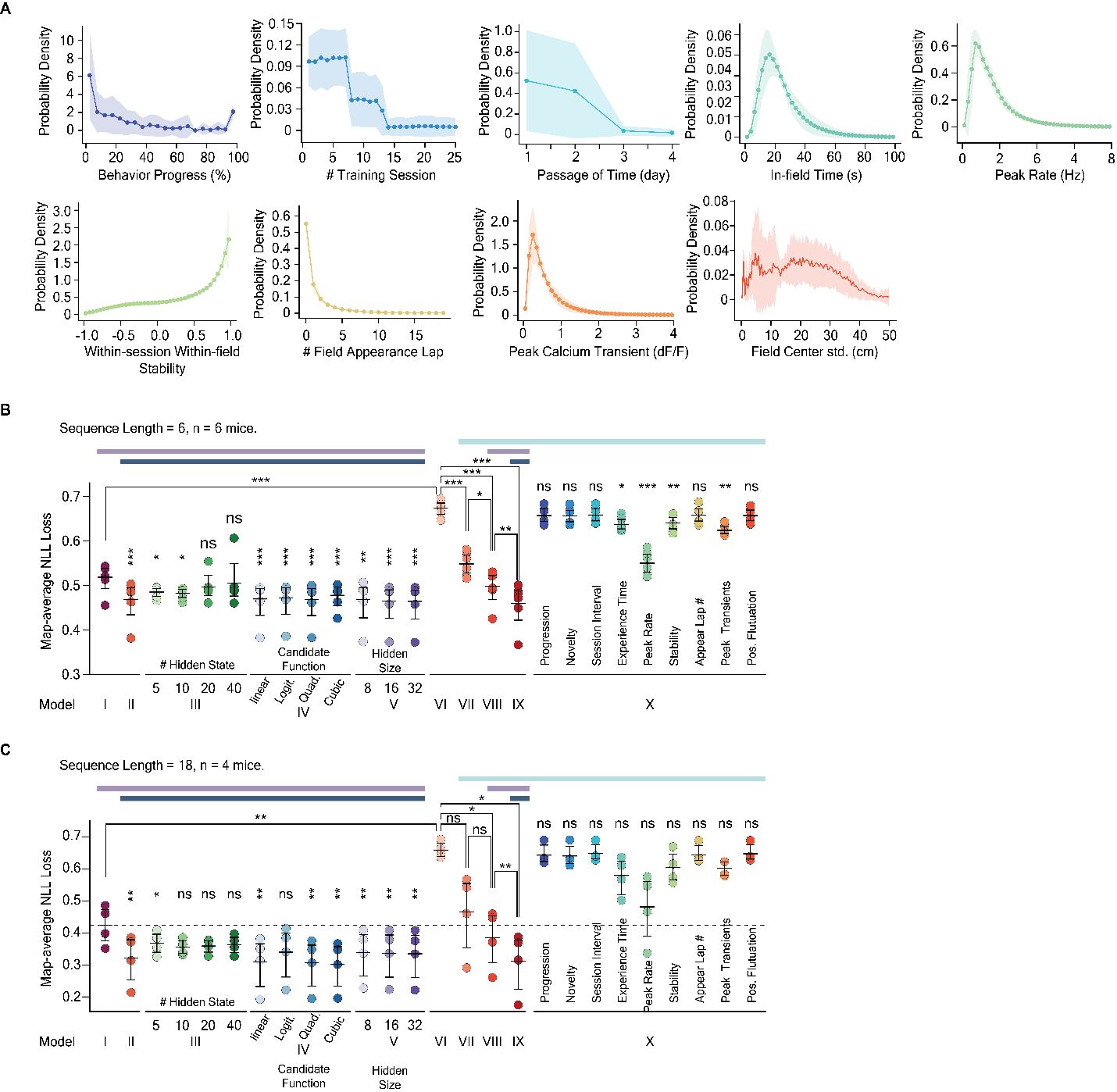


Figure S18. SFER-based models perform better than element-based models

(**A**) Distribution of all 9 elements. n = 30 spatial maps. Error bands: sd. (**B**-**C**) Prediction accuracy of all models at sequence lengths of 6 (**B**, n = 6 mice) and 18 (**C**, n = 4 mice). The top-colored bands represent the decoding strategies (Purple: field state-based prediction; Blue: history-dependent prediction; Light cyan: element-based prediction) each model employed. Abbreviations: Logit. = Logistic function; Quad. = Quadratic function. Stars above Models II to V indicate significance levels compared with Model I, while stars above Model X indicate significance levels compared with Model VI. Two-sided paired t-tests with Bonferroni correction were conducted for all statistical comparisons. NLL: Negative Log-likelihood. Significance levels: ns: P ≥ 0.005; *: P < 0.005; **, P < 0.001; ***, P < 1 × 10^-3^; ****, P < 1 × 10^-4^.


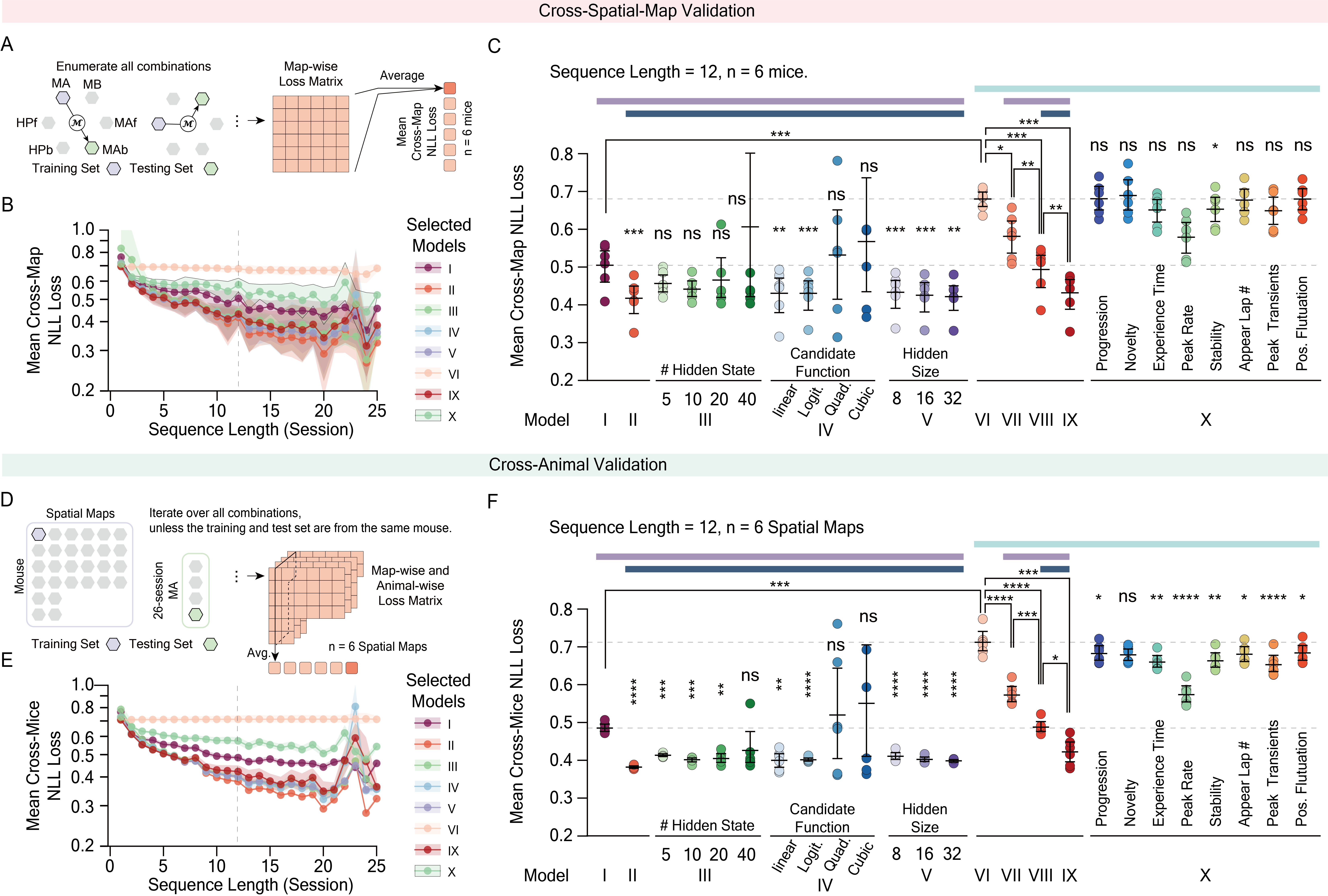


**Figure S19. Cross-spatial-map and cross-animal validations to examine overfitting.**

(**A-C**) Results from Cross-Spatial-Map validations. (**A**) Schematic of cross-spatial-map validation. Training and testing sets consisted of field-state sequences from different spatial maps, generating a map-wise loss matrix for each mouse. Values from this matrix were averaged to yield a mean cross-map loss per mouse. (**B**) Mean cross-map NLL loss decreases as sequence length increases. Eight representative models are shown (Model III: HMM with 10 hidden states; Model IV: SFER with linear function; Model V: GRU with hidden size 16; Model X: element-based model using peak rate). (**C**) Mean cross-map NLL loss of all models at sequence length 12, n = 6 mice. (**D–F**) Cross-animal validations. (**D**) Schematic of cross-animal validation. Testing sets were derived from Maze A (MA) data of four mice (#10224, #10227, #10232, #10234). Training sets could come from any spatial map as long as they were not from the same mouse. For each model, we obtained six average losses by training on MA, MB, MAf, MAb, HPf, or HPb and testing on MA. (**E**) Mean cross-animal NLL loss decreases with sequence length. Same eight models as in (**B**) are shown. (**F**) Mean cross-animal NLL loss of all models at sequence length 12, n = 6 spatial maps. In (**C**) and (**F**), top-colored bands represent decoding strategies: purple, field-state–based prediction; blue, history-dependent prediction; light cyan, element-based prediction. Stars above Models II–V indicate significance levels relative to Model I; stars above Model X indicate significance relative to Model VI. Abbreviations: Logit. = Logistic; Quad. = Quadratic. Two-sided paired t-tests with Bonferroni correction were used for all comparisons. Error bars represent 95% confidence intervals. Significance levels: ns: P ≥ 0.005; *: P < 0.005; **, P < 0.001; ***, P < 1 × 10^-3^; ****, P < 1 × 10^-4^.

**Table S1 Summary of cell and field registration results**

I: the number of registered neurons

| Spatial Map | 10209 | 10212 | 10224 | 10227 | 10232 | 10234 |
| --- | --- | --- | --- | --- | --- | --- |
| MNP, MA | 727 | 920 | 2518 | 3154 | 2994 | 7043 |
| MNP, MB | 651 | 1589 | 1624 | 1502 | 1945 | 3548 |
| RMP | 808 | 846 | 1446 | 1082 |  |  |
| HMP | 865 | 945 | 652 | 916 |  |  |

II: the number of registered neurons tracked across all sessions

| Spatial Map | 10209 | 10212 | 10224 | 10227 | 10232 | 10234 |
| --- | --- | --- | --- | --- | --- | --- |
| MNP, MA | 60/8.3% | 90/9.8% | 36/1.4% | 81/2.6% | 16/0.5% | 0/0 |
| MNP, MB | 58/8.9% | 46/2.9% | 95/5.9% | 154/10.25% | 69/3.5% | 0/0 |
| RMP | 15/1.9% | 69/8.2% | 29/2.0% | 286/26.4% |  |  |
| HMP | 40/4.6% | 56/5.9% | 112/17.2% | 275/30.5% |  |  |

III: percentage of neurons included in the analyses of the SFER in the preliminary sense

(# neurons included in the analysis / # neurons detected in all sessions (percentage))

| Map | 10209 | 10212 | 10224 | 10227 | 10232 | 10234 |
| --- | --- | --- | --- | --- | --- | --- |
| MA | 2000/2923 (68.4%) | 3787/4664 (81.2%) | 7422/10895 (68.1%) | 13054/16822 (77.6%) | 10751/14486 (74.2%) | 4327/11679 (37.0%) |
| MB | 2403/3361 (71.5%) | 2960/4457 (66.4%) | 3844/5466 (70.3%) | 7012/8533 (82.2%) | 4874/6733 (72.4%) | 1984/5590 (35.5%) |
| MAf | 1701/3081 (55.2%) | 3201/4206 (76.1%) | 1289/2845 (45.3%) | 3575/4622 (77.3%) |  |  |
| MAb | 1616/3081 (52.5%) | 3139/4206 (74.6%) | 1214/2845 (42.7%) | 3521/4622 (76.2%) |  |  |
| HPf | 777/2008 (38.7%) | 1381/2354 (58.7%) | 2299/3022 (76.1%) | 3212/4667 (68.8%) |  |  |
| HPb | 693/2008 (34.5%) | 1321/2354 (56.1%) | 2326/3022 (77.0%) | 3476/4622 (75.2%) |  |  |

Average percentage: 63.9 ± 14.7% (mean ± std.)

IV: the number of registered place fields

| Spatial Map | 10209 | 10212 | 10224 | 10227 | 10232 | 10234 |
| --- | --- | --- | --- | --- | --- | --- |
| MA | 6269 | 12064 | 29562 | 38086 | 21614 | 43481 |
| MB | 6874 | 16178 | 15902 | 18797 | 14478 | 18960 |
| MAf | 3827 | 6962 | 6148 | 5799 |  |  |
| MAb | 4765 | 7394 | 6017 | 6039 |  |  |
| HPf | 4400 | 8054 | 6227 | 6956 |  |  |
| HPb | 3539 | 7323 | 5812 | 6909 |  |  |

V: the number of registered fields included in the analyses of SFER in the preliminary sense

| Map | 10209 | 10212 | 10224 | 10227 | 10232 | 10234 |
| --- | --- | --- | --- | --- | --- | --- |
| MA | 3190/50.9% | 6949/57.6% | 10810/36.6% | 17535/46.0% | 10888/50.4% | 6362/14.6% |
| MB | 4062/59.1% | 6241/38.6 | 6888/43.3% | 11855/63.1% | 7019/48.5% | 3297/17.4% |
| MAf | 1763/46.1% | 4100/58.9% | 1661/27.0% | 3739/64.5% |  |  |
| MAb | 2166/45.5% | 4432/59.9% | 1766/29.4% | 3891/64.4% |  |  |
| HPf | 1707/38.8% | 3942/48.9% | 4294/69.0% | 4999/71.9% |  |  |
| HPb | 1430/40.41% | 3463/47.3% | 4004/68.9% | 4903/71.0% |  |  |

Average percentage: 48.7 ± 15.0% (mean ± std.)

VI: the number of registered fields included in the analyses of SFER in the general sense

(i.e., the number of registered fields that provided at least one field-state sequence)

| Map | 10209 | 10212 | 10224 | 10227 | 10232 | 10234 |
| --- | --- | --- | --- | --- | --- | --- |
| MA | 788/12.6% | 1556/12.9% | 3736/12.6% | 6416/16.8% | 2768/12.8% | 890/2.05% |
| MB | 838/12.2% | 1062/6.6% | 1762/11.1% | 2233/11.9% | 891/6.2% | 123/0.65% |
| MAf | 695/18.2% | 2386/34.3% | 261/4.25% | 1660/28.6% |  |  |
| MAb | 831/17.4% | 2542/34.4% | 278/4.62% | 1634/27.1% |  |  |
| HPf | 549/12.5% | 1296/16.1% | 1411/22.7% | 1881/27.0% |  |  |
| HPb | 467/13.2% | 1084/14.8% | 1283/22.1% | 1807/26.2% |  |  |

Average percentage: 16.1 ± 9.2% (mean ± std.)

VII: the percentage of fields used for the analyses of SFER in the preliminary sense

(# fields included in the analysis / # fields identified in all sessions (percentage))

| Map | 10209 | 10212 | 10224 | 10227 | 10232 | 10234 |
| --- | --- | --- | --- | --- | --- | --- |
| MA | 10681/16446 64.9% | 29795/42020 70.9% | 51142/89195 57.3% | 78259/120792 64.8% | 38155/60492 63.1% | 19583/64825  30.2% |
| MB | 14282/21073 67.8% | 22562/39378 57.3% | 24021/39255 61.2% | 35653/51260 69.6% | 18405/30168 61.0% | 7453/25826 28.9% |
| MAf | 4347/8392 51.8% | 14948/22402 66.7% | 3600/9477 38.0% | 9453/13323 71.0% |  |  |
| MAb | 5583/10642 52.5% | 14883/22161 67.2% | 3406/8656 39.3% | 8698/12306 70.7% |  |  |
| HPf | 3408/6883 49.5% | 8802/14404 61.1% | 8515/12069 70.6% | 9282/12528 74.1% |  |  |
| HPb | 2548/5078 50.2% | 7202/12348 58.3% | 8062/11354 71.0% | 9259/12632 73.3% |  |  |

Average percentage: 59.1 ± 12.2% (mean ± std.)

VIII: the percentage of fields used for the analyses of SFER in the preliminary sense

(# fields included in the analysis / # fields identified in all sessions (percentage))

| Map | 10209 | 10212 | 10224 | 10227 | 10232 | 10234 |
| --- | --- | --- | --- | --- | --- | --- |
| MA | 5032/16446 (30.6%) | 12386/42020 (29.5%) | 28969/89195 (32.5%) | 42689/120792 (35.3%) | 42689/120792 (35.3%) | 5783/64825 (8.92%) |
| MB | 5556/21073 (26.4%) | 7722/39378 (19.6%) | 10372/39255 (26.4%) | 10917/51260 (21.3%) | 10917/51260 (21.3%) | 552/25826 (2.14%) |
| MAf | 2468/8392 (29.4%) | 11889/22402 (53.1%) | 994/9477 (10.5%) | 5932/13323 (44.5%) |  |  |
| MAb | 3168/10642 (29.8%) | 11473/22161 (51.8%) | 832/8656 (9.61%) | 5025/12306 (40.8%) |  |  |
| HPf | 1561/6883 (22.7%) | 4227/14404 (29.3%) | 3823/12069 (31.7%) | 4603/12528 (36.7%) |  |  |
| HPb | 1053/5078 (20.7%) | 3231/12348 (26.2%) | 3595/11354 (31.7%) | 4558/12632 (36.1%) |  |  |

Average percentage: 28.6 ± 11.9% (mean ± std.)

**Table S1. Summary of cell-tracking results.**

(**I**) Number of registered neurons. MNP: Maze-navigation paradigm; RMP: reversed-maze paradigm; HMP: hairpin-maze paradigm. MA: Maze A; MB: Maze B. Neurons were shared between forward and backward laps in both RMP and HMP. Note that the number of registered neurons only indirectly reflects recording and registration quality, as it increases with longer recording durations and stricter registration criteria. (**II**) Number and proportion of neurons tracked across all sessions. This metric also indirectly reflects data quality, as the proportion inevitably approaches zero with longer recordings due to both technical limitations and inherent representational drift. It does not imply restricted data inclusion in SFER analyses, as we focused on *continuously tracked neuron pieces*, which include segments extracted from both continuously and intermittently tracked neurons. (**III**) Percentage of neurons included in the analyses of SFER in the preliminary sense. **Denominator**: total neurons detected across all sessions (summed over sessions). **Numerator**: neurons included at least once in the analysis. A continuously tracked neuron piece of length *L* contributes *L* neurons to the analysis. On average, 63.9 ± 14.7% of neurons were included, indicating broad data coverage. (**IV**) Number of registered place fields. (**V**, **VI**) On average, 48.7 ± 15.0% and 16.1 ± 9.2% of all registered place fields were included in the analyses of SFER in the preliminary (**V**) and general (**VI**) senses, respectively. For SFER in the general sense, only field-state sequences of length ≥ 10 (for MA and MB) or ≥ 5 (for MAf, MAb, HPf, and HPb) were included, requiring corresponding neuron pieces to be continuously tracked across the same number of sessions. (**VII**, **VIII**) Proportion of place fields included in SFER analyses (preliminary (**VII**) and general (**VIII**) senses). **Denominator**: total place fields identified across all sessions (summed over sessions). **Numerator**: total place fields included at least once in the analysis.

For mice #10209 and #10212, cells and place fields were tracked across 13 sessions (Stage 2, MA and MB). For mice #10224, #10227, #10232, and #10234, tracking covered 26 sessions in MA and 13 sessions in MB. For MAf, MAb, HPf, and HPb, cells and fields were tracked for 7–12 sessions. Analyses of SFER in the preliminary sense correspond to **Figures 2–3** and **S13–S15**; analyses of SFER in the general sense correspond to **Figures 4–6** and **S16–S19**.
